# How to make land use policy decisions: Integrating science and economics to deliver connected climate, biodiversity, and food objectives

**DOI:** 10.1073/pnas.2407961121

**Published:** 2024-11-13

**Authors:** Ian J. Bateman, Amy Binner, Ethan T. Addicott, Ben Balmford, Frankie H. T. Cho, Gretchen C. Daily, Anthony De-Gol, Sabrina Eisenbarth, Michela Faccioli, Henry Ferguson-Gow, Silvia Ferrini, Carlo Fezzi, Kate Gannon, Ben Groom, Anna B. Harper, Amii Harwood, Jon Hillier, Mark F. Hulme, Christopher F. Lee, Lorena Liuzzo, Andrew Lovett, Mattia C. Mancini, Robert Matthews, James I. L. Morison, Nathan Owen, Richard G. Pearson, Stephen Polasky, Gavin Siriwardena, Pete Smith, Pat Pat Snowdon, Peter Tippett, Sylvia H. Vetter, Shailaja Vinjili, Christian A. Vossler, Robert T. Watson, Daniel Williamson, Brett H. Day

**Affiliations:** ^a^Land, Environment, Economics and Policy Institute, University of Exeter Business School, Exeter EX4 4PU, United Kingdom; ^b^Department of Biology, Stanford University, Stanford, CA 94305; ^c^School of Environmental Sciences, University of East Anglia, Norwich NR4 7TJ, United Kingdom; ^d^Swiss Institute for International Economics and Applied Economic Research, University of St. Gallen, St. Gallen 9000, Switzerland; ^e^Department of Economics and Management, School of International Studies, University of Trento, Trento 38122, Italy; ^f^Centre for Biodiversity and Environment Research, Department of Genetics, Evolution and Environment, University College London, London WC1E 6BT, United Kingdom; ^g^Department of Economics and Management, University of Trento, Trento 38122, Italy; ^h^Grantham Research Institute, London School of Economics and Political Science, London WC2A 2AE, United Kingdom; ^i^Department of Geography, University of Georgia, Athens, GA 30602; ^j^Global Academy of Agriculture and Food Systems, The Royal (Dick) School of Veterinary Studies and The Roslin Institute, Easter Bush Campus, Midlothian EH25 9RG, United Kingdom; ^k^The British Trust for Ornithology, Thetford, Norfolk IP24 2PU, United Kingdom; ^l^Forest Research, Farnham, Surrey GU10 4LH, United Kingdom; ^m^Department of Applied Economics, University of Minnesota, St. Paul, MN 55108; ^n^Institute of Biological and Environmental Sciences, School of Biological Sciences, University of Aberdeen, Aberdeen AB24 3UU, United Kingdom; ^o^Policy and Practice, Scottish Forestry, Edinburgh EH11 3XD, United Kingdom; ^p^Exeter Clinical Trials Unit, University of Exeter, Exeter EX1 2LU, United Kingdom; ^q^Department of Economics and Baker School of Public Policy and Public Affairs, University of Tennessee, Knoxville, TN 37996

**Keywords:** decision-making, land use, natural capital, climate change, biodiversity

## Abstract

Land use change is crucial to addressing the existential threats of climate change and biodiversity loss while enhancing food security [M. Zurek *et al.*, *Science* **376**, 1416–1421 (2022)]. The interconnected and spatially varying nature of the impacts of land use change means that these challenges must be addressed simultaneously [H.-O. Pörtner *et al.*, *Science* **380**, eabl4881 (2023)]. However, governments commonly focus on single issues, incentivizing land use change via “Flat-Rate” subsidies offering constant per hectare payments, uptake of which is determined by the economic circumstances of landowners rather than the integrated environmental outcomes that will be delivered [G. Q. Bull *et al.*, *Forest Policy Econ.* **9**, 13–31 (2006)]. Here, we compare Flat-Rate subsidies to two alternatives: “Land Use Scenario” allocation of subsidies through consultation across stakeholders and interested parties; and a “Natural Capital” approach which targets subsidies according to expected ecosystem service response. This comparison is achieved by developing a comprehensive decision support system, integrating new and existing natural, physical, and economic science models to quantify environmental, agricultural, and economic outcomes. Applying this system to the United Kingdom’s net zero commitment to increase carbon storage via afforestation, we show that the three approaches result in significantly different outcomes in terms of where planting occurs, their environmental consequences, and economic costs and benefits. The Flat-Rate approach actually increases net carbon emissions while Land Use Scenario allocation yields poor economic outcomes. The Natural Capital targeted approach outperforms both alternatives, providing the highest possible social values while satisfying net zero commitments.

## Significance

Policies that alter land use affect food production, wild species, and greenhouse gas emissions simultaneously. However, policy analyses rarely if ever consider these connections and instead typically address climate change, biodiversity loss and food security as separate challenges. To address these “system” connections we integrate natural, physical, and economic knowledge and models to deliver an integrated decision support tool. This is used to assess the performance of common approaches to land use policy and highlight alternatives which simultaneously address all these integrated challenges and deliver improved outcomes.

The choice between different approaches to decision-making is no mere technical issue; if different approaches yield different results, then how we make decisions changes the decisions we make. While decisions on the incentivizing of land use changes to enhance ecosystem services are a matter of government policy (1–8), the allocation of agricultural subsidies is typically left to market forces and the resultant outcomes are shaped by the nature of the incentives which are offered. Subsidies are commonly offered at a constant per hectare (ha) “Flat-Rate” across a region or country, with minimal eligibility criteria. Indeed, over $50 billion per year, nearly a quarter of all agricultural subsidies globally, are offered on a per-area basis (9). This includes the European Union’s Common Agricultural Policy, the United Kingdom’s (UK’s) Environmental Land Management scheme, and China’s Sloping Lands program (10). While such schemes are easy to administer, the uptake of these incentives is determined not by the likely environmental outcomes of land use change, but solely by farmers comparing those payments with any agricultural revenues foregone. This Flat-Rate approach to decisions is a major contributor to policy failure with the UN (11) recently classifying 87% of the more than $800 billion in agricultural subsidies paid by countries worldwide each year (9, 12) as harmful and a major source of negative impacts on food security and the environment (11). An alternative “Land Use Scenario” approach allocates agri-environmental subsidies using the preferences of groups including landowner representatives, affected communities, and officials, often with input from experts in relevant fields (13). Such approaches have grown in influence, being the basis of the UK National Ecosystem Assessment (UK-NEA) (14), and have in some cases been mandated requirements for subsidy allocation (15). More recently, the integration of environmental science with economic analysis provided by the Natural Capital framework (7, 8) has raised the potential for targeting subsidies according to their expected outcomes. This framework has now been adopted as the basis of the UK Government’s 25 Year Environment Plan (16) which framed its 2020 Agriculture and 2021 Environment Acts and has been incorporated into the HM Treasury guidelines for appraising public sector spending (17).

Here, we provide the first systems-wide comparison of the current Flat-Rate approach to subsidy allocation with the alternatives provided by the Land Use Scenario and Natural Capital targeted approaches to land-use policy decision-making. While one may consider that Flat-Rate payments and Land Use Scenario decision-making approaches represent different priorities, often they simply reflect naive “muddling through” policy making (18). The present work builds on previous research exploring how budgets should be targeted to maximize the benefits from one specific environmental service (19–21), and the importance of including costs in the selection of sites for conservation (22). We apply all three decision-making approaches to the same real-world challenge, chosen for its global resonance. Like almost every other country in the world, the UK is a signatory to the United Nations Framework Convention on Climate Change (UNFCCC) Paris Agreement (23). To comply with this, the UK has committed itself to attaining net zero emissions of greenhouse gases (GHGs) by 2050 (24). Analyses have shown that, even if emission reductions pledges are honored in full (25), they will be insufficient to attain net zero and that GHG removal from the atmosphere will also be required (26). Of the options available, land use change is seen as essential (27–29) with afforestation identified as the GHG removal method which combines the highest CO_2_ removal potential with lowest per ton costs and greatest technology readiness level (29); *SI Appendix*. Assessments by the Royal Society have identified that (contingent on emissions reductions being put in place) a 2050 target of 13 MtCO_2_ per annum (pa) of removals via new afforestation is consistent with attaining net zero (29).

To allow for a consistent and systems-based comparison of all three decision approaches, we develop a cutting-edge, integrated, and user-focused decision support system, the Natural Environment Valuation (NEV) tool (30–32). This appraises not only the immediate policy goal of GHG removal but also connected impacts on biodiversity, food security, timber production, and recreation. This networks together new and preexisting models to consider these wider consequences of land-use change from farming to woodland. All impacts are quantified and valued using state-of-the-art economic valuation techniques, with the exception of biodiversity where a lack of robust valuation methods (8) means that quantified impacts are used as a measure of the consequences of each approach for biodiversity. The choice of biodiversity metric is discussed in *SI Appendix* which presents details on all elements of the analysis.

Analysis of the Flat-Rate approach uses economic modeling of agricultural land-use decisions (33, 34) to examine the expected uptake of forestry subsidies up to some set target. The setting of that target is clearly crucial, and one might expect it to be led by the science of GHG removal. However, in practice, targets have typically been determined by the budget allocated or, as in the case of UK policy, through a target for the area of woodland planted. In our analysis, we take the area specified in the UK-NEA (14) of roughly 2 million ha of new woodland. This sets up a further empirical focus of interest as to whether such an approach, focused on area units, will deliver the necessary carbon storage.

In contrast to the purely financial drivers of the Flat-Rate allocation of subsidies, the Land Use Scenario approach engaged a large and diverse group of stakeholders including representatives from Government Departments and Agencies, the business and NGO sector, and the research community (35). Undertaken as part of the UK-NEA, this exercise produced various scenarios for future land use and tree planting of which the Nature@Work scenario was considered the most beneficial (14). This envisaged the creation of roughly 2 million ha of new woodland and is adopted for comparison with the other subsidy allocation methods to cast the Land Use Scenario approach in its most favorable light.

The Natural Capital targeted approach considers both the market (agricultural production of food and timber output) and wider nonmarket (carbon storage, biodiversity, and recreation) consequences of creating new woodland. While accounting for predicted environmental benefits in the prioritization of areas for agri-environmental subsidies is rare, it is not unheard of and is discussed in various reviews (36, 37). Indeed, the United States’ Conservation Reserve Program uses an environmental benefit index to rank projects and distribute funding, targeting areas which offer greater environmental uplift (38). As we envisage the Natural Capital targeted approach, combinatorial optimization techniques (39) are used to find the set of planting locations and areas which maximize the net benefits of land use change subject to a policy objective. Again, setting that area target at approximately 2 million ha of afforestation, and additionally sufficient to meet 13 MtCO_2_ removal.

The integrated NEV models make it possible to undertake comprehensive and consistent comparisons of the impacts of the different geographical distributions of woodland creation generated by each of the three decision-making approaches under consideration. For each, we assess their effects on agricultural and timber output, net GHG emissions, recreation, and biodiversity, accounting for climate change in all cases. Economic valuation of all but the last of these effects provides a cost–benefit analysis which can then be compared to nonmonetized biodiversity outcomes with the trade-off between the two revealing the economic net benefit (or cost) associated with changes in those biodiversity outcomes.

We additionally use the integrated NEV models to undertake one further analysis illustrating the limitations of relying on some predefined target, rather than net benefit maximization. By removing all area and carbon storage constraints, we can find the area and distribution of woodland planting which maximizes unconstrained net benefits and provides a comparator against which to evaluate how well the different allocation mechanisms perform.

## Results

**Fig. 1.**
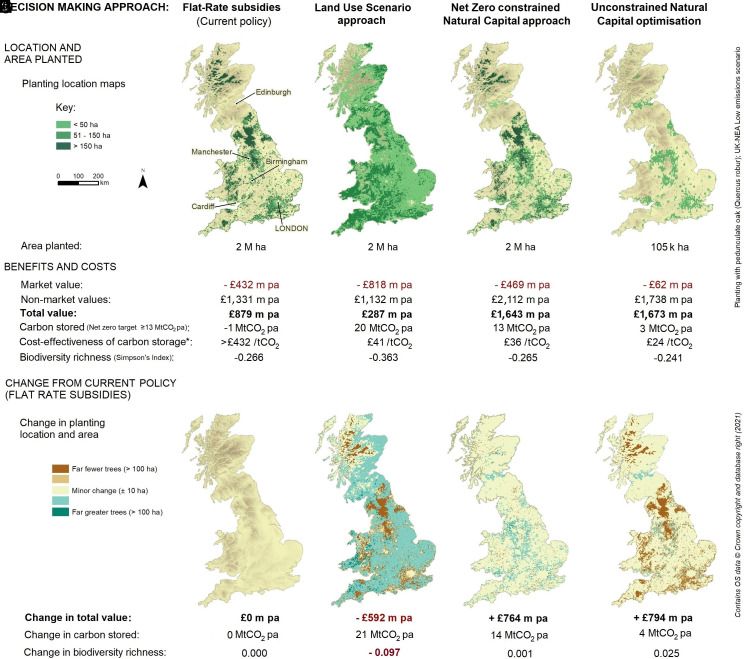
Spatial distribution and resulting benefits and costs from planting new woodland in Great Britain under alternative decision-making approaches (*Upper* row) and changes relative to the currently used Flat-Rate payment approach (*Lower* row). (*A*–*C*) show the location of approximately 2 million ha of new woodland planted under three decision-making approaches: (*A*) Flat-Rate payment (the current approach to decision-making); (*B*) Land Use Scenario allocation (from the UK-NEA); (*C*) Natural Capital targeted approach when constrained to deliver the UK 2050 net zero target of 13 MtCO_2_e per annum removal of greenhouse gases via trees. (*D*) removes the latter constraint and delivers the area and location of planting which maximizes monetized total values. (*E*–*H*) the change in planting location between each approach (*A*–*D*) and the current method for determining planting locations (Flat-Rate payment; *A*). * The cost of carbon calculation only considers the direct policy cost of payments to landowners and farms and does not include either any global emissions associated with carbon leakage via increased food imports or the nonmarket benefits arising under each option. The calculation for the flat rate approach is purely indicative as this policy generates net emissions of carbon. All values are in GBP(£) base year 2013. All analyses control for climate change (see text).

Results are illustrated in Fig. 1 where maps 1a-c show the spatial distribution of new afforestation as determined using the Flat-Rate payment, Land Use Scenario, and Natural Capital targeted approaches, respectively. Here, the Flat-Rate payment map (Fig. 1*A*, which also indicates major British cities) shows that forest planting is clustered into those areas where the financial returns to farming are lowest such that planting subsidies are relatively more attractive. These are predominately upland areas such as the Scottish Highlands, the Cambrian Mountains of Wales, and the uplands of England such as the Pennines and Lake District (topographic shading is given in Fig. 1*E*). Subsidies are required to compensate farmers for the market value losses of converting from agriculture to forestry and this annual subsidy cost is substantial, totaling more than £430 million pa. However, the nonmarket, ecosystem service benefits generated by this planting are substantial with considerable recreation values created by new woodlands near to cities such as London and Manchester. Overall net benefits are nearly £900 million pa, giving a benefit cost ratio which exceeds much public spending (40). In the absence of any further comparison, results from this analysis of the standard Flat-Rate payment approach to afforestation, as compared to a business-as-usual scenario with no afforestation, might well lead the decision-maker to consider such a planting scheme to be a value-for-money investment, well worth proceeding with.

However, a major caveat to the Flat-Rate payment approach arises when we consider its contribution to net zero goals. Here, subsidy uptake is determined according to the difference between subsidies and current agricultural values, irrespective of carbon storage. Low agricultural productivity farms are frequently located on poor quality but carbon rich soils (e.g., in upland locations). As a result, tree growth is poor, and planting acts to dry out peaty soils resulting in GHG emissions rather than sequestration such that the net consequence of planting trees according to the Flat-Rate payment approach is not GHG removal but rather carbon release. Indeed, taken together, this result shows the Flat-Rate payment approach provides relatively poor cost-effectiveness against the policy’s overarching objective of carbon sequestration; in short, the approach to policy implementation is a major determinant of policy effectiveness.

Results from the Flat-Rate payment approach highlight a more general problem affecting all approaches concerning biodiversity. Climate change (which is consistently incorporated in all analyses; *SI Appendix*) is a significant [if far from sole (41, 42)] driver of biodiversity loss over this period, extending long term declines in UK wild species (43). All approaches reveal biodiversity losses relative to the present day showing that planned afforestation, even with the native species, as we assume here (44), is insufficient to conserve Britain’s wild species and highlights the need for separate measures to address this problem. To control for climate change effects, the impacts of afforestation on biodiversity (and indeed other ecosystem services) are therefore best considered as relative comparisons across the different decision-making methods. These relative comparisons are shown in the lower panel of Fig. 1 *E*–*H* which take the current Flat-Rate payment approach as a baseline and present differences from that in terms of planting distribution, net value, carbon storage, and biodiversity.

The Fig. 1*B* shows that the Land Use Scenario approach results in a radically different distribution of the same 2 million ha of tree planting. This is now spread at relatively low intensity across almost all areas, except for the Scottish Highlands where explicit avoidance of planting on peatlands results in carbon storage substantially exceeding target levels. As set out in the UK-NEA (14), one of the objectives of the Land Use Scenario exercise was to promote equity across regions. It has long been recognized that multiple objectives cannot be maximized with a single policy instrument and that trade-offs are inevitable (45); this is the so-called Tinbergen Rule (46). The comparison of the Land Use Scenario with other approaches (which do not adopt this procedure) provides an interesting perspective on such trade-offs. The difference from the Flat-Rate payment approach is mapped in Fig. 1*G*. This shows that the Land Use Scenario approach includes considerable planting across the rural lowlands of England. This is the most agriculturally productive area of Britain and the region for which the costs of compensating for forgone farm output are highest. Indeed, the results show that the costs of this approach are nearly double those under the current Flat-Rate payment method resulting in a marked fall in overall net benefits relative to current practice. Planting on such high-quality land results in substantial improvements in tree growth and carbon storage. Evaluating this against the direct financial costs of the scheme yields an improvement in the cost-effectiveness of carbon storage. However, such a planting scheme also adds to existing challenges to farmland birds [which have declined by nearly 60% over the past half century (43)] and biodiversity declines even further than under the current Flat-Rate payment approach.

Considering results from the Natural Capital targeted approach (Fig. 1*C*), an initial observation is that this avoids the very diffuse planting dictated by the Land Use Scenario allocation and, at first glance, appears to provide a distribution of afforestation similar to the Flat-Rate payment approach. However, the difference from the latter is highlighted in Fig. 1*H* which shows that the Natural Capital targeted approach results in a greater concentration of planting in and around major urban areas, which generates improved recreational access to high quality environments for these large urban populations. As some of these locations border high productivity agricultural areas, subsidy costs exceed those of the Flat-Rate payment approach but are nearly one-quarter lower than under the Land Use Scenario method which planted across a wide swathe of highly productive agricultural land. However, the major distinguishing feature of the Natural Capital targeted approach is the very high level of nonmarket benefits it generates, roughly 50% and 75% higher than the Flat-Rate payment and Land Use Scenario approaches, respectively. Its greater use of productive farmland than the former (though not as extreme as the latter) also results in higher and more cost-effective carbon storage, and reaches the net zero requirement of removing 13 MtCO_2_ annually by 2050. In addition to satisfying this requirement, the Natural Capital targeted approach delivers a much greater level of net benefits, being well over four times as valuable as the Land Use Scenario approach and 50% greater than the Flat-Rate payment approach. Biodiversity is also significantly greater than the former and similar to that under the latter approach.

The three comparisons undertaken so far apply different decision-making approaches to the same overarching objective to plant approximately 2 million ha of new woodland in Great Britain (GB). As can be seen, switching between these alternative approaches results in massively different outcomes in terms of the location of planting, the costs incurred and benefits generated, carbon storage, and biodiversity. This is an important result given that the Flat-Rate payment approach is the most commonly applied method for distributing subsidies while Land Use Scenario allocations are increasingly popular. Only the integration of science and economics offered by the Natural Capital targeted approach provides the ability to satisfy the multiple objectives we have for land-use change. So, can this approach be used with impunity?

The integrated nature of the environmental science and economic models incorporated within the NEV decision support system readily allow us to consider alternative policy questions. One obvious question is what are the consequences of applying a simple cost–benefit rule where “unconstrained” net benefit maximization is used to direct land use change, devoid of either planting area or carbon storage constraints. Fig. 1*D* shows the somewhat striking result that arises when we plant purely to maximize the total value of land use change as estimated using economic methods. The net benefits of afforestation are maximized by planting just over 100,000 ha of additional woodland; roughly 5% of that envisioned under the previous analyses. The resulting pattern of planting determined by this approach retains periurban locations with high recreational values but avoids peatlands and other areas where afforestation would result in net GHG emissions, as well as locations where the opportunity cost of foregone agriculture outweighs the corresponding increases in benefits. Interestingly this approach delivers the best outcome for biodiversity (42) as well as the most cost-effective carbon storage.

Moreover, unconstrained Natural Capital optimization reduces total subsidies to one-tenth of those using Natural Capital targeting to select areas for the 2 million ha of afforestation. Despite costs falling by over 85%, nonmarket benefits decline by only 18% resulting in £1,673 million pa of net benefits. While these far exceed net benefits derived from the Flat-Rate and Land Use Scenario approaches, this is achieved at the sacrifice of the GHG removal targets maintained by the constrained Natural Capital approach and necessary to deliver net zero. In short, the unconstrained optimization of net benefit values does not deliver a sustainable solution here and underscores that to deliver against multiple objectives requires multiple policy instruments (46, 47). Ensuring those objectives through the net zero constrained Natural Capital approach delivers over 98% of the maximum possible net benefits, and achieves societal objectives in a highly efficient manner.

## Discussion and Conclusions

The Flat-Rate payment approach to the implementation of policy objectives is so globally commonplace that its relative inefficiency goes unnoticed. The present analysis lays these failings bare and shows that the decision-making approach adopted to implement policy can have a very substantial impact on the effectiveness of that policy. Similarly, our comparison with the Land Use Scenario approach shows that, in its commonly applied form, the Flat-Rate payment approach can also deliver poor value for money to the taxpayers funding land use subsidies. Arguably, the approach can be defended due to its focus on spatial equity but the significant trade-offs in terms of reduced food security and lower net benefits suggest that there might well be scope for more efficient delivery of equity objectives.

In contrast, by uniting the natural, physical, economic, and social sciences, the integrated Natural Capital targeted approach permits a significantly more efficient allocation of scarce resources allowing multiple land use objectives to be addressed. Indeed, the Natural Capital targeted approach also highlights the need for all policy aims to be explicitly agreed upon, and direct decision-making, rather than allow for heuristics to guide how policies are implemented.

As a final caveat, we do not see the Natural Capital targeted approach as a replacement for the involvement of policymakers or stakeholders in decision-making. Issues such as competing demands upon tax revenues (e.g., health services, education, etc.) and the democratic involvement of communities will always require such incorporation if decisions are to be made acceptable (48). Rather we see cutting-edge decision support systems, such as the NEV modeling suite, as tools for bringing a greater understanding of the implications of alternative policies into the decision-making process. Future research might usefully test the impact of such tools on stakeholder analyses, as well as policy making outcomes.

## Materials and Methods

This section summarizes the models and approaches considered in the paper. Full details regarding all models, their data, and resolution are provided in *SI Appendix*. Together, this clarifies the methodological aspects of this contribution, providing a decision support system capable of accepting multiple user inputs in terms of scenarios of land use futures (31), changes in policy, or optimizing for user defined objectives such as net zero (the latter contributions being extensions to our prior research).

### Models.

#### Agriculture.

Full data, model development, and analysis details are provided in *SI Appendix*. In summary, agricultural data include the amount of farmland in use, areas of the all the major crops (e.g., cereals, oilseed rape, root crops, temporary grassland, permanent grassland, rough grazing, etc.), and head-counts for livestock (e.g., dairy cows, beef cows, and sheep). This information was taken from a combination of Defra Agricultural Census (49) 2 km square grid resolution across GB, and Farm Business Survey (FBS) records, which every year collects detailed production information for a panel of about 2000 farms located in England (50).

Other land use data were obtained from the CEH Land Cover Map (51, 52), the National Inventory for Woodland and Trees (53), and the UK-NEA (14). Elevation and slope data at 50 m resolution were taken from the CEH Integrated Hydrological Digital Terrain Model (54, 55). Soil data were taken from the Harmonised World Soil Database (HWSD) (56), while the climate data and climate change predictions were provided by the Met Office (57) and Hadley Centre for Climate Prediction and Research (58). Data were analyzed by extending spatially explicit, climate-sensitive, structural econometric modeling of GB agriculture (31, 33, 34, 59–61). Out of sample, actual versus predicted value testing showed that the resultant model provided highly accurate estimates of prior agricultural land use. These models were then applied dynamically to predict annual farm profitability and land use under each of the three analyses, allowing for climate change, out to the year 2064. This annual time step and assessment period was applied consistently across all analyses. While environmental and other shocks may increase real (i.e., inflation adjusted) farmgate prices, technological change tends to produce the opposite effect. In the absence of clear evidence to the contrary, the model assumes constant real prices though these can be adjusted in light of new information.

#### Tree growth and timber production.

Potential timber yields for afforestation with either representative conifer (*Sitka spruce*, although nonnative the most commonly planted commercial species in the UK) or native broadleaf (*Pedunculate oak*) species on each 250 m grid cell across GB were estimated using the Forest Research Ecological Site Classification (ESC) model (62) using data on site soil and other physical environment characteristics and historic climate data. By utilizing the substantial spatiotemporal variation in climate across the country, the ESC growth rate data were used to develop a new, highly flexible, nonlinear, climate sensitive model which was used to predict future spatial and temporal variation in yield under specified climate change estimates.

**Fig. 2.**
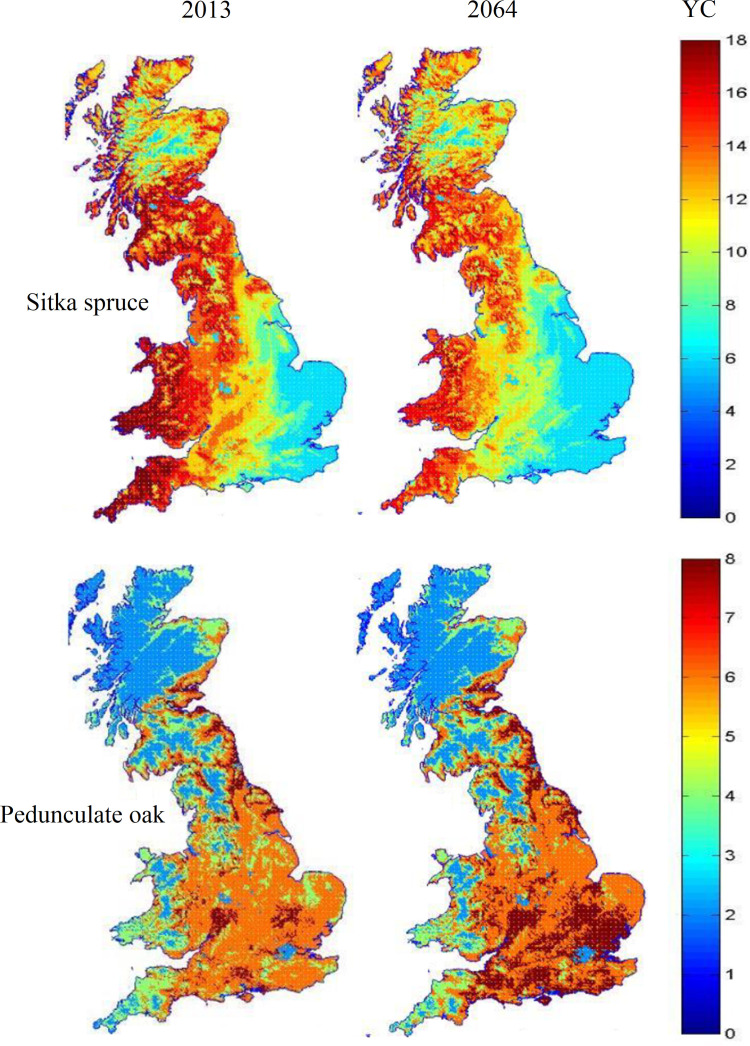
Expected tree growth rates (measured as yield class; YC, average m^3^/ha/y) across GB for two species, Sitka spruce (*Upper* row) and pedunculate oak (*Lower* row) under current climate conditions (*Left* hand column) and those predicted for 2060 (*Right* hand column). Source: Adapted from (37).

Fig. 2 presents the spatial distribution of estimated tree growth for Sitka spruce (*Upper* row maps) and pedunculate oak (*Lower* row maps), under climatic conditions in 2013 (*Left* hand maps) and expected climate change to 2064 (*Right* hand maps). Reviewing growth rates in 2013, we see that *Sitka spruce* grows fastest in the cooler, damper upland areas to the west and north although growth rates decline in the very highest parts of the central Scottish highlands. Conversely pedunculate oak prefers the warm lowlands of south-eastern England. Moving to consider the estimates for 2064 it is remarkable to note that, while this is just a few short decades away, the impact of intervening climate change is highly significant. Warmer weather and lower summer rainfall adversely affects Sitka spruce growth right across the country. In effect, the optimal conditions for the species are disappearing at the edge of the sea. Given that this is the most commonly planted commercial crop species in GB, this is of considerable financial concern. Conversely, the warmer weather increases pedunculate oak yield class noticeably, particularly in its favored lowland locations. Given the very short period under consideration here compared to the rotation length of even fast-growing conifers, the scale of these changes is both remarkable and worrying.

The Forest Research CARBINE model (63–65) was used to estimate GHG balances and timber volumes from the yield and species information, and timber revenues were calculated using the FC Forest Investment Appraisal Package (FIAP) (66, 67) taking into account the nonlinear relationship between timber volumes and price. The standard UK public sector discount rate (17) was applied to this and other time delayed benefits and costs; again this can be adjusted as desired.

#### Agricultural greenhouse gases.

Agriculture is a substantial emitter of GHGs through, for example, machinery use, mineral and organic fertilizer use, and ruminant livestock. Major carbon pools on land persist in living biomass (forests, perennials, and tree-cropping systems), in addition to soil carbon. To capture this, the spatially and temporally sensitive Cool Farm Tool (CFT) (68, 69) models of the major agricultural GHGs (CO_2_, N_2_O, NO, and CH_4_) were linked to the farmland use and livestock intensity model within NEV. This allows GHG flows to be calculated as a function of land-use (crop type or livestock type), intensity, and management (including fertiliser type and application) from the NEV agricultural model, and a range of soil parameters [soil texture, moisture, drainage, pH, bulk density, and SOM; data taken from the HWSD (56)]. CFT incorporates the life cycle of agricultural emissions and its programming within NEV allows the analysis of the GHG consequences of land use change between farming and forestry.

#### Forestry greenhouse gases.

The CARBINE model (63–65) relates the spatially and climate sensitive model of tree growth described above to estimates of the annual GHG flows arising from woodland and the afforestation of land. The model accounts for the emissions and sequestration associated with standing trees, deadwood and forest litter, roots and soil carbon change, and harvested wood products (HWP), and adjusts for management regime. All GHG measures were expressed as tons of CO_2_ equivalent (tCO_2_e) and calculated for both the representative conifer (*Sitka spruce*) and broadleaf (*Pedunculate oak*) species taking account of the variation in end-uses associated with these different species.

All analyses were extended well into the future to allow for the long time periods associated with the consequences of planting trees. Forest GHG balances are relatively sensitive to the choice of time period considered in decision-making, both because of the slow growth rate of trees and because of the extended periods over which soil carbon changes equilibrate. All of these factors were incorporated into the analysis to permit inspection of the impacts of changing the period under consideration.

#### Recreation.

Recreational behavior data were taken from the Monitor of Engagement with the Natural Environment (MENE) survey (70), a mass sample survey in which randomly selected households complete diary entries concerning their recreational activities. This was supplemented with spatially referenced information regarding the environmental characteristics and qualities of sites which are, or are not, visited by households (25). Estimates of the costs of visits, including direct expenditures, travel time, and foregone alternatives, are also calculated and take into account variation in travel infrastructure. Small area census information provides further data on the socioeconomic characteristics of potential visitors.

Econometric modeling techniques are then applied to relate individuals’ recreational choices (including the choice to not visit a site) to site, cost, and personal characteristics. This reveals the impacts of change in those characteristics (e.g., a change of a site from farmland to woodland; the establishment of woodland at different distances from a population) upon visitation behavior and the implicit value which those changes generate. Importantly, this analysis captures the strong spatial dependence that exists in recreational behavior. Visits to a site are determined not only by the characteristics of that site and its accessibility to populations but also the other sites around that potential destination and the location of substitute sites relative to outset locations.

By linking the recreational analysis to the land use model within the NEV tool, we quantify the recreational value of any proposed land use change.

#### Biodiversity.

The choice of biodiversity metric is a focus of ongoing scientific debate and switches between metrics can have significant impacts on analyses (71–73). Given this, we adopt a commonly used species diversity measure [Simpson’s Index (74)] applied to breeding birds. The use of birds to measure and monitor biodiversity is well supported by data (75) and the literature (76), with the decline in bird numbers being of longstanding concern in the United Kingdom (77).

Data were taken from the Breeding Bird Survey (75) and were collected at a 1 km square resolution during the period 1999–2011 from across GB (*SI Appendix* for details). Multiple biodiversity metrics were generated from these data (78) and we report the most general of these in the paper. By relating these data to corresponding spatial and temporal physical characteristic and land use data (see *SI Appendix, Table 1.1* subsequently), we develop a model of the impact of land use and land use change on the diversity of breeding birds across GB. This is integrated into the overall NEV modellng suite and used to examine the impact of land use change upon measures of biodiversity.

#### The NEV integrated model.

The individual models are programmed together through our custom-built NEV decision support system. As discussed above, the land use model is acted upon by three sets of drivers: i) policy drivers such as land use subsidies, regulations on permitted uses, etc.; ii) market drivers, such as the price of crops, the costs of fuel and other inputs, etc.; and iii) environment drivers including spatially variable factors such as soil type and temporal variable drivers such as climate change. The systems nature of the environment means that this land use change induces responses in all connected systems and these effects are captured in the NEV modules. A shift in agricultural land use causes change in other land uses, either directly (e.g., though afforestation of previous farmed land) or indirectly (e.g., through responses in GHG emissions or storage, changes in wild species habitat and biodiversity and changes in recreational behavior). The programmed linkages within the NEV system yield rapid estimates of all these responses assessed as quantities and, where robust valuation is possible, as economic values (all but the biodiversity effects).

All modules within the NEV decision support suite are programmed together to ensure real time analysis of the consequences of alternative decisions. This combined with the spatial and temporal nature of all modules permits the use of a variety of optimisation techniques which can be applied to maximize some objective.

To address the dynamics inevitably triggered when a huge area of land is afforested over a long period, the NEV tool utilizes the processing speed of its interlinked models to employ combinatorial optimization techniques (39). These take the user-defined planting horizon and area (here 2 million ha), and optimize over all possible spatial and temporal combinations of this planting while allowing for the spatial dependence relevant to some benefits (e.g., recreation). Optimization can then be conducted for the desired objective; for the Flat-Rate payment analysis, this is restricted to the financial sum of foregone agricultural value, timber revenue, and minimized subsidies; for the Natural Capital targeted approach, this is extended to also consider GHGs and recreation, with biodiversity impacts assessed purely in quantity terms. Further dynamics, in particular, the effect of land use change on food production, imports, and hence the potential leakage of carbon emissions and biodiversity loss overseas, are the subject of ongoing research and extension of the NEV model including integration with computable general equilibrium trade models.

### Decision-Making Approaches.

#### Flat-rate payment.

The Flat-Rate payment analysis assesses uptake of conventional government subsidies for afforestation under the assumption that they are available to all farms. Uptake of subsidies is determined purely by their financial value to the farmer, which is based on the size of those subsidies, the discounted financial benefits of timber production value, and the costs to farmers of foregone agricultural profits.

The financial value of planting trees on existing farmland is simply equal to the benefits of timber revenues minus the costs of foregone agricultural production. Our analysis shows that in almost all locations, this value is negative, which explains both the low level of afforestation in the UK and the necessity of subsidies to induce the planting needed to attain GHG removal targets and satisfy the 2050 net zero commitment. When subsidies are provided, farmers who face the lowest opportunity costs (i.e., those for whom agricultural profits are lowest) will take up those subsidies. The NEV model links the agriculture and timber models to reveal which locations convert from agriculture to woodland as subsidies are increased. In effect, the analysis is equivalent to minimizing the total cost of subsidizing the roughly 2 million ha planting target.

#### Land use scenario approach.

The Land Use Scenario analysis was conducted as part of the UK-NEA project that engaged with a large and diverse group of stakeholders, as detailed in *SI Appendix* where we also summarize methods [full details described in detail in the UK-NEA main report and supplementary papers (14, 35)]. The UK-NEA produced six scenarios for future land use and tree planting of which the Nature@Work scenario was identified as delivering the greatest level of ecosystem services (14). We adopt this scenario for comparison with the Flat-Rate payment and Natural Capital targeted approaches to cast the Land Use Scenario approach in its most favorable light.

As noted elsewhere (79), the Land Use Scenario approach lacks a clear implementation mechanism and relies upon an unspecified planning approach which we see as one of its drawbacks.

#### Natural capital targeted approach.

The Natural Capital targeted approach uses the same models of agricultural decision-making and timber production as described above for the Flat-Rate payment method. However, our implementation of the Natural Capital targeted approach through the NEV decision support suite extends the appraisal to also consider impacts upon biodiversity, GHGs (both from agriculture and forestry), and recreation, the modeling for which is summarized below and discussed in detail subsequently within *SI Appendix*.

Implementation of the Natural Capital targeted approach is achieved by targeting subsidies according to benefits estimated by the analysis. The efficiency of this approach can be further improved by implementation through an auction mechanism which invites bids from land managers for land use change and compares these with expected benefits to maximize value for money (80–82).

## Supplementary Material

Appendix 01 (PDF)

## Data Availability

Outputs from the Natural Environment Valuation (NEV) tool quantifying different aspects of market and non-market value for each of the decision-making approaches (Flat-Rate payment, Land Use Scenario and Natural Capital targeted) are available for download at the Harvard Dataverse (https://doi.org/10.7910/DVN/POGPP2) (83).
