# Supplementary material for: How to make land use policy decisions: Integrating science and economics to deliver connected climate, biodiversity, and food objectives
Source: Proc Natl Acad Sci U S A. 2024 Nov 13;121(49):e2407961121. doi: 10.1073/pnas.2407961121 (PMC11626137; doi:10.1073/pnas.2407961121)
Supplement: Supplementary file 1 — Appendix 01 (PDF) [file pnas.2407961121.sapp.pdf]

PNAS MS#2024-07961RR

Supporting Appendix

How to make land use policy decisions: Integrating science and economics to deliver connected climate, biodiversity and food objectives

Ian J. Bateman[1], Amy Binner, Ethan Addicott, Ben Balmford, Frankie H.T. Cho, Gretchen C. Daily, Anthony De-Gol, Sabrina Eisenbarth, Michela Faccioli, Henry Ferguson-Gow, Silvia Ferrini, Carlo Fezzi, Kate Gannon, Ben Groom, Anna B. Harper, Amii Harwood, Jon Hillier, Mark F. Hulme, Christopher F. Lee, Andrew Lovett, Lorena Liuzzo, Robert Matthews, Mattia C. Mancini, James I.L. Morison, Nathan Owen, Richard Pearson, Stephen Polasky, S., Gavin Siriwardena, Pete Smith, Pat Snowdon, Peter Tippett, Sylvia Vetter, Shailaja Vinjili, Christian A. Vossler, Robert T. Watson, Daniel Williamson and Brett H. Day.

1.1: The Natural Environmental Valuation (NEV) modelling suite: Introduction and overview

The Natural Environmental Valuation (NEV) modelling suite (1) is a modular, integrated system of natural science to socio-economic behaviour models designed to provide support for decisions regarding land-use in Great Britain. Combining environmental science, econometric and process modelling, the NEV modelling suite provides two principal outputs:

(i) Land use at a high level of spatial resolution and temporally out to the end of the analysis period, a detailed and quantified understanding of the environmental, economic and policy drivers of that land use, and estimates of how land use will respond to changes in those drivers (e.g. ongoing climate change, shifts in the prices and costs of land use related products such as different food outputs, changes in agricultural, forestry, environmental and other land use related policy);

(ii) The ecosystem service related goods and services which arise from land use and how they change in response to land use change. Within the present analysis we quantify the impacts of land use and land use change on food production, timber output, storage and emissions of key land use related greenhouse gases (CO_2, CH_4, N_2O), biodiversity and recreation[2]. In addition to quantification, all of these ecosystem services, with the exception of biodiversity, are also assessed in terms of their economic value (i.e. their contribution to welfare, irrespective of whether they have market prices or not). Biodiversity is not expressed in monetary terms due to the lack of robust economic valuation methods and so is protected using no-loss rules applied to potential decisions.

[1]Land, Environment, Economics and Policy Institute (LEEP), University of Exeter Business School (UEBS), Exeter, UK. Corresponding author email: i.bateman@exeter.ac.uk. ORCiD ID: 0000-0002-2791-6137

[2] Ongoing extensions consider the impact of land use and land use change on water quality and flood risk. For discussion see (1).

The NEV modelling suite is a spatially and temporally explicit decision support tool which shows the user where and when land use change arises in response to shifts in driver and how, where and when ecosystem service benefits and losses are accrued. Both land use change and its consequences are assessed and displayed via maps, quantities and values. A number of interacting spatial scales are considered, including a grid of $2km^2$ (400 hectare) cells used for the agricultural production and associated farm greenhouse gas emissions or storage modelling (with information held on the number of hectares of each production types within each cell but their precise locations within that cell withheld for data protection reasons); tree growth, timber production, and associated greenhouse gas storage and emission similar resolution for greenhouse gas storage and emission and, at much higher resolution, recreational parks and paths and predicted visitation rates[3]. In terms of the temporal scale, the NEV modelling suite predicts ecosystem service flows and values into the future. Assessments are produced at an annual timestep. All outputs are provided from at least 2020 and 2060 with certain analyses extended further into the future[4].

The remainder of this section provides an overview of the data used in this analysis. Subsequent sections provide a technical summary of each of the individual modules within the NEV decision support system. The modular nature of NEV is designed to permit ready and continual updating of individual models as they are improved, ensuring that the suite does not become ossified.

1.2: Data

Table 1.1 provides information on the various data incorporated within the NEV decision support suite. Each data layer is described in some detail including sources (including URLs), additional references where helpful, and notes on the processing of data prior to its use within NEV.

One of the challenges of an exercise such as this arises where data collected at different points in time utilise differing definitions of variables. This arose with respect to the land use data given in the UK Centre for Ecology & Hydrology (UKCEH) Land Cover Maps (LCMs) and so Table 1.2 provides additional notes regarding the definition of land use classes. Further information and definitions of the variables used in the NEV land use analysis are provided subsequently.

---

[3] Ongoing extensions of NEV incorporate hydrological sub-catchments and basins connected by a river network.

[4] For example, valuations association with forestry and tree-related carbon storage require much longer timescales to be considered. Current work extends most modules to at least 2100.

Table 1.1: Data incorporated within the NEV decision support suite; for further information see (2).

Layer [Ms reference]	Description [appropriate year(s)]	Processing notes	Caveats or limitations to use	Dependencies (input data); URL(s) (if appropriate) - last accessed April 2022; Extra references pertinent to processing (if appropriate)
Farm_slope [TERRAIN]	Proportion of land that is farmland AND greater than six degrees inclination [2012]	Derived from the 50 m resolution IHDTM (obtained as an ASCII raster and manipulated in a GIS). Average elevation for a 2 km cell was simply the aggregate of all 1,600 elevation values in the corresponding IHDTM grid divided by the sum of cells. Slope (degrees inclination) was calculated from the 50 m IHDTM as the maximum rate of change in value from a cell to its eight neighbours. An average slope value was then taken for an entire cell. Further to these two standard average-per-2 km-cell variables (slope and elevation), farmland-specific variables (here, farmland is inclusive and defined as all crops, grasses and other land on farms) were calculated. Average elevation on farmland was calculated as a weighted average from a 25 m resolution base definition of farmland (from LCUAP2 2010); in practice, this operation was: sum for 2 km cell the following: (elevation × (area farmland/area of land)). The approach was similar for slope. A final terrain variable was the proportion of land that is farmland AND greater than six degrees inclination.	0.1 m vertical resolution, was originally derived (by CEH) from Ordnance Survey 1:50,000 mapping and vector data. This dataset was selected for its high quality and anticipated hydrological consistency.	Integrated Hydrological Digital Terrain Model (IHDTM), licensed from the Centre for Ecology and Hydrology, Wallingford. Version 2002. Accessed 2012. See Morris and Flavin, 1990; 1994; https://www.ceh.ac.uk/services/integrated-hydrological-digital-terrain-model; Relies on the definition of farmland also derived herein. See LCUAP2 2010. Refs: Morris D. G., and Flavin, R. W., 1990. A digital terrain model for hydrology. Proc. 4th International Symposium on Spatial Data Handling. Vol 1 Jul 23-27 Zurich, 250-262; Morris D. G., and Flavin, R. W., 1994. Sub-set of UK 560 m by 50 m hydrological digital terrain model grids. NERC, Institute of Hydrology, Wallingford.
Soil [SOIL]	Various soil variables [2012]	Categorisation of variables as follows: topsoil texture class (coarse, medium, fine, none); broad soil types (clay, loam, loamy sand, sandy loam, clay/loam, sand, silt loam, urban/lake); management-related phase (stony, lithic, fragipan, saline, gravelly, no-phase); pH (<4.5, 4.5-5.5, 5.5-7.2, 7.2-8.5, >8.5); total organic carbon (<0.2, 0.2-0.6, 0.6-1.2, 1.2-2.0 2.0-25, >25); gravel; texture (adjusted %weight sand per cell, adjusted %wt silt per cell, adjusted %wt clay per cell - adjusted for area of land in cell); reference depth (0, 30, 100); obstacles to roots (class 0 -4); impermeable layer (class 0-4); FAO drainage (class 0-6); available water storage capacity	When using these variables for model estimation, be careful not to use 'overlapping' categories (e.g. soil type and wt fractions).<br>Percentage area values are per cell, not land area nor agricultural area. Values have been rounded to two decimal places.<br>Particularly relevant for a UK-based study, the areas covered	Harmonized World Soil Database (HWSD), FAO/IIASA/ISRIC/ISSCAS/JRC. Version 2009. Accessed 2012. See: https://www.fao.org/3/aq361e/aq361e.pdf<br>Latest data URL: https://www.fao.org/soils-portal/soil-survey/soil-maps-and-databases/harmonized-world-soil-database-v12/en/

		(class 0-6); dominant annual average soil water regime (class 1-4). Derived from HWSD and pertains to the topsoil (0-30cm) unless stated otherwise. The source raster data (approx. 1 km resolution) was converted into vector format to allow the addition of an attribute table and the intersection of the 2 km grid. Percentage totals are in each class of interest in the 2 km cell was then taken, or area-weighted averages were taken if more appropriate.	by SOTER, including Central and Eastern Europe, are considered to have the highest reliability in the HWSD (SOTER = World SOil and TERrain Digital Database project, which has an intended 1: 1,000,000 scale).	
Climate [CLIMATE]	Rainfall and temp. [baseline 1960-1990] and predictions [to 2099]	Accumulated annual rainfall and mean temperature in the crop growing season (April to September) were calculated by simply averaging or summing the 30 monthly or annual 5 km gridded data sets for each variable. These data were then interpolated to values for the central points of 2 km cells using bilinear interpolation. Where necessary (boundaries of grid with no climate data), the value of the adjacent cell was used.	Long-term average data (30 years from 1961-1990) at 5 km input resolution.	Gridded Observation Data, UKCP09, Met Office, available from: http://www.metoffice.gov.uk/climatechange/science/monitoring/ukcp09/index.html<br>Full report: (5).<br>Latest data URL: https://www.metoffice.gov.uk/research/climate/maps-and-data/data/haduk-grid/datasets
Greenbelt [GREENBELT_EW]	Percentage area of greenbelt in England and Wales [2012]	Defra provided a file for greenbelt in England. Welsh greenbelt was digitised to clip to road and county boundaries using information found in local plans. The 2 km grid was then overlain and the percentage area of greenbelt in the cell was calculated from the intersection of the two datasets.	Temporarily variable data obtained where relevant (new designations or changes to boundaries). Welsh data digitised so some generalisation inherent.	GLG Greenbelt, England spatial data. Licensed for use on the UK NEA_FO project from Defra (NR0150). Latest data URL: https://data.gov.uk/dataset/ccb505e0-67a8-4ace-b294-19a3cbff4861/english-local-authority-green-belt-dataset<br>Newport Unitary Development Plan (1996-2011): https://www.newport.gov.uk/documents/Planning-Documents/LDP-2011-2026/adopted-UDP.pdf
Greenbelt [GREENBELT_S]	Percentage area of greenbelt in Scotland [2012]	Scottish greenbelt PDFs were georeferenced and digitised. The 2 km grid was then overlain and the percentage area of greenbelt in the cell was calculated from the intersection of the two datasets.	At the time of processing, there was no national digital spatial boundary dataset for Scottish greenbelt. Each council was contacted for spatial information and PDF maps or ESRI shapefiles were received for all areas of Scottish greenbelt (present and historic). Digitised so some generalisation inherent. Input scales typically ranging from 1:8000 to 1:25000	Boundary data interpreted from PDF documents retrieved from individual councils in Scotland.

Designated [DESIG]	Percentage area of designated land under classes of National Park, Nitrate Vulnerable Zones and Environmentally Sensitive Areas. [2012]	National Parks are protected areas of the countryside and, although the land is often privately owned and worked, National Parks welcome visitors. Formal designation of land into National Parks has been staggered since the first Parks in the 1950s. At the time of processing, there were 15 National Parks across Great Britain. Spatial boundary data for National Parks were downloaded from MAGIC, Countryside Council for Wales and Scottish Government Spatial Data File Download website. These data sources were also used to download digital spatial data for Environmentally Sensitive Areas (ESA) (zones, not agreements, which run up to 2014) and Nitrate Vulnerable Zone (NVZ)	Temporarily variable data obtained where relevant (new designations or changes to boundaries).	Nitrate Vulnerable Zones Designations (England), Environment Agency accessed via MAGIC.gov.uk in 2012; National Parks (England), Natural England accessed via MAGIC.gov.uk in 2012; Environmentally Sensitive Areas (England), Natural England accessed via https://magic.defra.gov.uk/ Welsh GIS data equivalents downloaded from Countryside Council for Wales (now Natural Resources Wales); http://lle.gov.wales/home?lang=en Scottish data equivalents downloaded in 2012 from Scottish Government Spatial Data (http://crtb.sedsh.gov.uk); https://spatialdata.gov.scot/geonetwork/srv/eng/catalog.search#/home
Market_distance [TRAVEL_CITY]	Travel time to market [2012]	Travel time to nearest urban area with total population > 300,000 was calculated as follows. First, urban areas with large populations were identified from the 2001 Census (KS01). There were 12 urban areas with populations exceeding 300 thousand people in 2001. These names were then matched to DLUA spatial data boundaries. Travel times are calculated from the centroid of a 2 km to the nearest urban border (DLUA). Travel is via the GB road network (see below). Travel time calculations were undertaken using the 'Cost Distance' (impedance surface) command in ESRI ArcGIS. First, the Meridian 2 road network (Motorway, A-road, B-road and minor roads) was converted into a regular grid of 100 × 100 m cells, with each cell containing a value corresponding to travel-time-per-unit distance. Road speeds were taken from (3) and allowances were made for locations off the regular road grid (adjustments for walking speed). The resultant travel time map is used to calculate the minimum travel time between any outset location and any destination.	Assumes nearest market is an urban area with a resident population > 300 000 people.	Ordnance Survey Meridian 2 road network (Motorway, A/B/minor roads) and Developed Land Use Area. Updated 2009. Polyline and Polygon files. OS Open Spatial data accessed 2013 and downloaded via: https://osdatahub.os.uk/downloads/open; https://www.ordnancesurvey.co.uk/opendatadownload/products.html 2001 Census (variable KS01). Accessed in 2012 via CASWEB UK Data Service Census support; https://casweb.ukdataservice.ac.uk/ Ref: Sen, A., Harwood, A., Bateman I.J., Munday P., Crowe A., Haines-Young R., Brander L., Provins, A., Raychaudhuri, J., Lovett, A., and Foden J., (2014) Economic Assessment of the Recreational Value of Ecosystems in Great Britain, Environmental and Resource Economics, Volume 57, Issue 2, pp 233-249, DOI 10.1007/s10640-013-9666-7
Landcover_2000 [LCUP1]	Percentage area under ten	The 25 m resolution raster product for LCM2000 (4) was used as raw land cover data for 2000. Ten land cover categories, consistent with habitat mapping as part of the	Remotely sensed data were acquired between November 1996 and May 2001 to	Forestry Commission. National Inventory for Woodland and Trees. Polygon data. Updated 2002. https://www.forestresearch.gov.uk/tools-

	landcover classes. [2000]	first phase of UK-NEA (106), were created from combining subclasses of land cover. Land cover classes: deciduous; coniferous; enclosed farmland; improved grassland; semi-natural grassland; mountains, moors and heaths; coastal margins; freshwater; marine; urban and developed land (see further notes in Table 0.2). Next, a simple cross-tabulation was performed to look at land cover change on a cell-by-cell basis across the two time periods (2007 below). Reasonable correlation with small changes in land cover were expected, e.g. due to development and small differences in the methodology between LCM2000 and LCM2007. However, the results of the comparison did not always perform as anticipated and there was considerable movement across many classes. These reclassified data were thus augmented with Forestry Commission boundaries of existing woodland, Ordnance Survey data on Roads and Railways and Developed Land Use Areas. These updates enabled a more reliable indication of non-agricultural land use extent.	generate the input dataset Land Cover Map 2000 (4)	and-resources/national-forest-inventory/national-inventory-of-woodland-and-trees/<br>Land Cover Map 2000 (LCM2000) 25 m raster grid, https://eip.ceh.ac.uk/lcm/lcmdata/previousversions/lcm2000 Ref: Fuller, R. M., Smith, G. M., Sanderson, J. M., Hill, R. A., Thomson, A. G., Cox, R., Brown, N. J., Clarke, R. T., Rothery, P., and Gerard, F. F., 2002. Countryside Survey 2000 Module 7: Land Cover Map 2000 final report. NERC/Centre for Ecology and Hydrology 100pp. (CEH Project Number: C00878); UK-NEA (2011).<br>Ordnance Survey Meridian 2 road network (Motorway, A-road, B-road and minor roads) and Developed Land Use Area. Polyline and Polygon files. Updated 2009. OS Open Spatial data accessed 2013 and downloaded via: https://www.ordnancesurvey.co.uk/opendatadownload/products.html; https://osdatahub.os.uk/downloads/open Ref: UK-NEA (2011) UK National Ecosystem Assessment: Technical Report. UNEP-WCMC, Cambridge, UK. Available via: http://uknea.unep-wcmc.org/
Landcover_2007 [LCUP2]	Percentage area under ten landcover classes. [2007]	The 25 m resolution raster product for LCM2007 (6) was used as raw land cover data for 2007. Ten land cover categories, consistent with habitat mapping as part of the first phase of UK-NEA (106), were created from combining subclasses of land cover. Land cover classes: deciduous; coniferous; enclosed farmland; improved grassland; semi-natural grassland; mountains, moors and heaths; coastal margins; freshwater; marine; urban and developed land. Further notes: Add notes for landcover def. Next, a simple cross-tabulation was performed to look at land cover change on a cell-by-cell basis across the two time periods (2000 above). Reasonable correlation with small changes in land cover were expected, e.g. due to development and small differences	Remotely sensed data were acquired between September 2005 and July 2008 to generate the input dataset Land Cover Map 2007 (6)	Forestry Commission. National Inventory for Woodland and Trees. Polygon data. Updated 2002. https://www.forestresearch.gov.uk/tools-and-resources/national-forest-inventory/national-inventory-of-woodland-and-trees/ Ref: UK-NEA (2011) UK National Ecosystem Assessment: Technical Report. UNEP-WCMC, Cambridge, UK. Available via: http://uknea.unep-wcmc.org/<br>Land Cover Map 2007 (LCM2007) 25 m raster grid.<br>https://eip.ceh.ac.uk/lcm/lcmdata/previousversions/lcm2007 Ref: Morton, D., Rowland, C., Wood, C., Meek, L., Marston, C., Smith, G., Wadsworth, R., Simpson, I.C., (2011). Final Report for

		in the methodology between LCM2000 and LCM2007. However, the results of the comparison did not always perform as anticipated and there was considerable movement across many classes. These reclassified data were thus augmented with Forestry Commission boundaries of existing woodland, Ordnance Survey data on Roads and Railways and Developed Land Use Areas. These updates enabled a more reliable indication of non-agricultural land use extent.		LCM2007 - the new UK Land Cover Map. Countryside Survey Technical Report No. 11/07 NERC/Centre for Ecology and Hydrology 112pp. (CEH Project Number: C03259). http://www.ceh.ac.uk/documents/LCM2007FinalReport.pdf<br>Ordnance Survey Meridian 2 road network (Motorway, A-road, B-road and minor roads) and Developed Land Use Area. Polyline and Polygon files. Updated 2009. OS Open Spatial data accessed 2013 and downloaded via: https://www.ordnancesurvey.co.uk/opendatadownload/products.html also https://osdatahub.os.uk/downloads/open
Landuse_2000 [LCUPAP1]	Percentage area under twenty five land use classes. [2000]	Overview: Satellite-derived land cover data and ancillary spatial data were used to locate areas that are likely to be functional e.g. used for agricultural production or urban activities. Results from agricultural survey data were used to refine the spatial distribution of arable and grassland and subdivide categorisation where appropriate. A Geographical Information System (GIS) was used to interrogate and integrate data to a base resolution of a 2 by 2 km cell.<br>In some cases land cover classes may be synonymous with land use. Often, however, variability of land use is greater than the variability of land cover because one land cover can fulfil different functions, i.e. the relationship is not one-to-one (9). Nevertheless, land cover data can provide a useful framework within which to map agricultural land use e.g. (7). Initially, relevant land areas from land cover derived data were compared with national-level June Survey statistics for agriculture (110). Considerable disparities in total areas were observed; from the agCensus product, it is possible for observations of agricultural land to exceed the physical area of zones (see discussion in 8; 7). Our testing found particular problems in Scotland and Wales. Subsequent results and analyses informed the following decisions: The 2 km level agCensus data could be used to subdivide	Rather than a complete land use definition, the resultant dataset is more adequately described as a high resolution database depicting potential land cover or land use area across Great Britain. Due to uncertainties with input data*, there is greater confidence in relative magnitudes of areas (i.e. shares of land types) than absolute totals. However, as the level of spatial aggregation increases, the absolute area totals become more accurate. Also, as the timeframe of study increases, to say three to five years, data become more representative of that period, rather than a single target year.<br><br>*Satellite-derived land cover data are aggregated from several years (see above). The June Survey of Agricultural	June Agricultural Survey. Agricultural region statistics. Version 2001. ERSA; June Agricultural Survey. County-level statistics. Version 2000. Defra; June Agricultural Survey. Small Area Statistics. Version 2003. National Assembly for Wales; https://data.gov.uk/dataset/332b5dfc-9616-47b2-81ee-4fcd407196ca/june-survey-of-agriculture-and-horticulture-england<br>Office for National Statistics (2011). Agriculture in the United Kingdom 2011. Office for National Statistics, Newport, UK. https://www.gov.uk/government/statistics/agriculture-in-the-united-kingdom-2011<br>June Agricultural Census (agCensus). 2km resolution table. GB extent. Version 2004. See: https://agCensus.edina.ac.uk/ and http://edina.ac.uk/agCensus/agcen2.pdf<br>Land Cover Map 2000 (see 4) https://eip.ceh.ac.uk/lcm/lcmdata/previousversions/lcm2000<br>OS county and region boundaries. Polygon file. Updated 2011. OS Open Data https://osdatahub.os.uk/downloads/open<br>Small area boundaries. Polygon file. Updated 2001. National Assembly for Wales.

		total arable land in a corresponding 2 km cell into different types of crops (fine resolution data were used to maintain local cropping patterns); Higher level geographies (i.e. administrative-level) were needed to define the total arable land in a 2 km cell and refine the distribution of types of grassland and grazing. Greater confidence was given to the administrative-level statistics as although these are aggregated for farms within an area, they are not subject to redistribution algorithms used in the production of the agCensus. County- and Unitary Authority-level June Survey data for 2000 were downloaded as a spreadsheet for England. Similar summaries were obtained for Welsh Agricultural Regions. Scottish regional data were obtained as PDF files from the Economic Report on Scottish Agriculture (ERSA). These administrative-level data were amalgamated into one dataset of 81 zones, each with six broad land use categories compatible in definition across time and for each country: Arable, horticulture & fallow; Temporary grassland; Permanent grassland; Sole-right rough grazing; Farm woodland; All other land on farm. Next, these tabulated data were joined to spatial boundary data in a GIS. At this stage, the implicit assumption was that the variables of interest (land use types) had a homogenous spatial distribution across source zones (administrative areas). It was then necessary to redistribute the above source zone data within the locations constrained by appropriate land cover classes. In other words, the high resolution (25 m × 25 m grid) reclassified land cover data (used to create e.g. LCUP1) were used to restrict probable locations for agricultural land use within each administrative area. Geographic boundaries for the administrative areas were overlain on the land cover grid. Given that the area of land use in each source zone was known, we satisfied these observations by scaling the 25 m resolution land cover-derived classes. Then, each broad land use type (at 25 m resolution) was summed for a set of final target zones – a regular grid of 2 km cells. Target zones of 1	and Horticultural Activity is a source of high quality land use data with national coverage. The June Survey is undertaken as a full census every ten years and as a sample survey in intervening years. The June Survey is undertaken independently in England, Scotland and Wales and results are released in aggregated spatial units. These data can either be obtained in the form of a regular grid known as the 'agCensus' (available at 2 km, 5 km and 10 km resolutions) or for administrative boundaries such as counties and regions. Due to protection against the disclosure of information on individual holdings, there are caveats associated with the use of these 'ready-made' datasets for spatially explicit research. Broadly speaking, agCensus data can be inaccurate at fine resolutions due to spatial reworking and re-distribution of holding data, and while statistics for administrative boundaries are more accurate, many data are suppressed to preserve anonymity or released at a higher level geography where the resolution is too coarse. To combat these shortfalls, both data formats were used.	https://lle.gov.wales/catalogue/item/AgriculturalSmallAreaStatistics/?lang=en<br>SEERAD (2001). Economic Report on Scottish Agriculture: 2001 Edition. Scottish Executive Environment and Rural Affairs Department, UK. https://www.gov.scot/collections/economic-report-on-scottish-agriculture/<br>Refs: Comber, A., Proctor, C., and Anthony, S., (2008). The creation of a national agricultural land use dataset: combining pycnophylactic interpolation with dasymetric mapping techniques. Transactions in GIS, 12, 775-791; Fuller, R. M., Smith, G. M., Sanderson, J. M., Hill, R. A., Thomson, A. G., Cox, R., Brown, N. J., Clarke, R. T., Rothery, P., and Gerard, F. F., 2002. Countryside Survey 2000 Module 7: Land Cover Map 2000 final report. NERC/Centre for Ecology and Hydrology 100pp. (CEH Project Number: C00878); Posen, P., Hutchins, M., Lovett, A., Davies, H., (2011). Identifying the catchment size at which robust estimations of agricultural land use can be made, and implications for diffuse pollution modelling. Applied Geography, 31, 919-929; SEERAD (2001). Economic Report on Scottish Agriculture: 2001 Edition. Scottish Executive Environment and Rural Affairs Department, UK.

		km were used for estimation of models. In the final step of processing, relevant crop types were extracted from the 2004 and 2010 agCensus (2 km resolution) datasets. Total Arable, horticulture & fallow land in the 2 km target zones were refined into different crop types using overlying agCensus data (by apply corresponding areal proportions). Therefore, the final dataset could be aggregated thematically or spatially to suit different research applications.		
Landuse_2010 [LCUPAP2]	Percentage area under twenty five land use classes. [2010]	Overview: Satellite-derived land cover data and ancillary spatial data were used to locate areas that are likely to be functional e.g. used for agricultural production or urban activities. Results from agricultural survey data were used to refine the spatial distribution of arable and grassland and subdivide categorisation where appropriate. A Geographical Information System (GIS) was used to interrogate and integrate data to a base resolution of a 2 by 2 km cell.<br>In some cases land cover classes may be synonymous with land use. Often, however, variability of land use is greater than the variability of land cover because one land cover can fulfil different functions, i.e. the relationship is not one-to-one (9). Nevertheless, land cover data can provide a useful framework within which to map agricultural land use (e.g. 7). Initially, relevant land areas from land cover derived data were compared with national-level June Survey statistics for agriculture (10). Considerable disparities in total areas were observed; from the agCensus product, it is possible for observations of agricultural land to exceed the physical area of zones (see discussion in 8; 7). Our testing found particular problems in Scotland and Wales. Subsequent results and analyses informed the following decisions: The 2 km level agCensus data could be used to subdivide total arable land in a corresponding 2 km cell into different types of crops (fine resolution data were used to maintain local cropping patterns); Higher level geographies (i.e. administrative-level) were needed to define the total arable land in a 2 km cell and refine the	Rather than a complete land use definition, the resultant dataset is more adequately described as a high resolution database depicting potential land cover or land use area across Great Britain. Due to uncertainties with input data*, there is greater confidence in relative magnitudes of areas (i.e. shares of land types) than absolute totals. However, as the level of spatial aggregation increases, the absolute area totals become more accurate. Also, as the timeframe of study increases, to say three to five years, data become more representative of that period, rather than a single target year.<br><br>*Satellite-derived land cover data are aggregated from several years (see above). The June Survey of Agricultural and Horticultural Activity is a source of high quality land use data with national coverage. The June Survey is undertaken as a full census every ten years	June Agricultural Survey. Agricultural region statistics. Version 2010. ERSA; June Agricultural Survey. County-level statistics. Version 2010. Defra; June Agricultural Survey. Small Area Statistics. Version 2010. National Assembly for Wales; https://data.gov.uk/dataset/332b5dfc-9616-47b2-81ee-4fcd407196ca/june-survey-of-agriculture-and-horticulture-england<br>Office for National Statistics (2011). Agriculture in the United Kingdom 2011. Office for National Statistics, Newport, UK; https://www.gov.uk/government/statistics/agriculture-in-the-united-kingdom-2011<br>June Agricultural Census (agCensus). 2km resolution table. GB extent. Version 2004. See: http://edina.ac.uk/agCensus/agcen2.pdf and https://agCensus.edina.ac.uk/<br>Land Cover Map 2007 (LCM2007) 25 m raster grid; https://eip.ceh.ac.uk/lcm/lcmdata/previousversions/lcm2007 (see 6).<br>OS county and region boundaries. Polygon file. Updated 2011. OS Open Data; https://osdatahub.os.uk/downloads/open<br>Small area boundaries. Polygon file. Updated 2001. National Assembly for Wales; https://lle.gov.wales/catalogue/item/AgriculturalSmallAreaStatistics/?lang=en;<br>(10). Economic Report on Scottish Agriculture: 2011 Edition. Scottish Government Rural

		distribution of types of grassland and grazing. Greater confidence was given to the administrative-level statistics as although these are aggregated for farms within an area, they are not subject to redistribution algorithms used in the production of the agCensus. County- and Unitary Authority-level June Survey data for 2010 were downloaded as a spreadsheet for England. Similar summaries were obtained for Welsh Agricultural Regions. Scottish regional data were obtained as PDF files from the Economic Report on Scottish Agriculture (ERSA). These administrative-level data were amalgamated into one dataset of 81 zones, each with six broad land use categories compatible in definition across time and for each country: Arable, horticulture & fallow; Temporary grassland; Permanent grassland; Sole-right rough grazing; Farm woodland; All other land on farm. Next, these tabulated data were joined to spatial boundary data in a GIS. At this stage, the implicit assumption was that the variables of interest (land use types) had a homogenous spatial distribution across source zones (administrative areas). It was then necessary to redistribute the above source zone data within the locations constrained by appropriate land cover classes. In other words, the high resolution (25 m × 25 m grid) reclassified land cover data (used to create e.g. LCUP2) were used to restrict probable locations for agricultural land use within each administrative area. Geographic boundaries for the administrative areas were overlain on the land cover grid. Given that the area of land use in each source zone was known, we satisfied these observations by scaling the 25 m resolution land cover-derived classes. Then, each broad land use type (at 25 m resolution) was summed for a set of final target zones – a regular grid of 2 km cells. Target zones of 1 km were used for estimation of models. In the final step of processing, relevant crop types were extracted from the 2004 and 2010 agCensus (2 km resolution) datasets. Total Arable, horticulture & fallow land in the 2 km target zones were refined into different crop types using	and as a sample survey in intervening years. The June Survey is undertaken independently in England, Scotland and Wales and results are released in aggregated spatial units. These data can either be obtained in the form of a regular grid known as the ‘agCensus’ (available at 2 km, 5 km and 10 km resolutions) or for administrative boundaries such as counties and regions. Due to protection against the disclosure of information on individual holdings, there are caveats associated with the use of these ‘ready-made’ datasets for spatially explicit research. Broadly speaking, agCensus data can be inaccurate at fine resolutions due to spatial reworking and re-distribution of holding data, and while statistics for administrative boundaries are more accurate, many data are suppressed to preserve anonymity or released at a higher level geography where the resolution is too coarse. To combat these shortfalls, both data formats were used. Specific caveats at the level of individual land use are given in Table 0.2<br>.	Payments and Inspections Directorate, UK; https://www.gov.scot/collections/economic-report-on-scottish-agriculture/<br>Ref: Morton, D., Rowland, C., Wood, C., Meek, L., Marston, C., Smith, G., Wadsworth, R., Simpson, I.C., (2011). Final Report for LCM2007 - the new UK Land Cover Map. Countryside Survey Technical Report No. 11/07 NERC/Centre for Ecology and Hydrology 112pp. (CEH Project Number: C03259). http://www.ceh.ac.uk/documents/LCM2007FinalReport.pdf

		overlying agCensus data (by apply corresponding areal proportions). Therefore, the final dataset could be aggregated thematically or spatially to suit different research applications.		
Livestock_2000 [Livestock1]	Headcount under four categories. Proxy for the distribution of animal excreta and manures. [2000]	Livestock were distributed over agricultural land (LCUPAP1) using stocking densities at administrative-level. Initial analysis and a review of literature (e.g. see 11; 7) informed the following rules: (1) Cattle were distributed at administrative-level across grassland (Temporary and Permanent); (2) Sheep were distributed at administrative-level across grassland (Temporary and Permanent) and Sole-right rough grazing; (3) Pigs and poultry were distributed at administrative-level across intensive agriculture (Arable, horticulture & fallow; and All other land on farm). Then, each livestock type (at 25 m resolution) was summed for the set of target zones – a regular grid of 2 km cells.	Caveats as above for the location of agricultural land (LCUPAP1). Poultry datasets were prepared to aid the estimation of nutrient export coefficients; however, the agricultural model did not predict poultry numbers due to lack of temporal data. Indoor or outdoor distinction of pigs and poultry is important (e.g. for water quality, see 7), but this was not possible due to a lack of spatial and temporal data.	Data as per LCUPAP1. Refs: Lyons, H. 2010. Methodology for the development of the ADAS land use database 2010. ADAS, UK. Available at: http://edina.ac.uk/agCensus/support/Methodology_1km_2010.pdf; Posen, P., Hutchins, M., Lovett, A., Davies, H., (2011). Identifying the catchment size at which robust estimations of agricultural land use can be made, and implications for diffuse pollution modelling. Applied Geography, 31, 919-929.
Livestock_2010 [Livestock2]	Headcount under four categories. Proxy for the distribution of animal excreta and manures. [2010]	Livestock were distributed over agricultural land (LCUPAP2) using stocking densities at administrative-level. Initial analysis and a review of literature (e.g. see 11; 7) informed the following rules: (1) Cattle were distributed at administrative-level across grassland (Temporary and Permanent); (2) Sheep were distributed at administrative-level across grassland (Temporary and Permanent) and Sole-right rough grazing; (3) Pigs and poultry were distributed at administrative-level across intensive agriculture (Arable, horticulture & fallow; and All other land on farm). Then, each livestock type (at 25 m resolution) was summed for the set of target zones – a regular grid of 2 km cells.	Caveats as above for the location of agricultural land (LCUPAP2). Poultry datasets were prepared to aid the estimation of nutrient export coefficients; however, the agricultural model did not predict poultry numbers due to lack of temporal data. Indoor or outdoor distinction of pigs and poultry is important (e.g. for water quality, see 7), but this was not possible due to a lack of spatial and temporal data.	Data as per LCUPAP2. Refs: Lyons, H. 2010. Methodology for the development of the ADAS land use database 2010. ADAS, UK. Available at: http://edina.ac.uk/agCensus/support/Methodology_1km_2010.pdf; Posen, P., Hutchins, M., Lovett, A., Davies, H., (2011). Identifying the catchment size at which robust estimations of agricultural land use can be made, and implications for diffuse pollution modelling. Applied Geography, 31, 919-929.

Notes: All data are spatially referenced tables permitting ready mapping. Spatial resolution is to 400ha (2km x 2km) cells. All data were processed by Amii Harwood.

Acknowledgements: Bespoke data were extracted by the Centre for Ecology and Hydrology, Department for Environment, Food and Rural Affairs, Environment Agency, Forestry Commission, Scottish Environment and Protection Agency, and devolved councils of the Scottish Government. All other sources are credited in the text. Data

are derived from digital spatial data licensed from the Centre for Ecology & Hydrology © NERC, contain Ordnance Survey data © Crown copyright and database right 2013, and may be based upon 1:625 000 geology data, used with the permission of the British Geological Survey. The UK Climate Projections data have been made available by the Department for Environment, Food and Rural Affairs (Defra) and Department for Energy and Climate Change (DECC) under licence from the Met Office, Newcastle University, University of East Anglia and Proudman Oceanographic Laboratory . These organisations accept no responsibility for any inaccuracies or omissions in the data, nor for any loss or damage directly or indirectly caused to any person or body by reason of, or arising out of, any use of this data. Furthermore, through the use of quality control procedures every effort is made to maintain and improve the quality and consistency of the river flow data. However, the Natural Environment Research Council, the Environment Agency, the Scottish Environment Protection Agency and the Rivers Agency (NI) accept no liability for any loss or damage, cost or claims arising directly or indirectly from their use. Contact for dataset queries: amii.harwood@uea.ac.uk

Table 1.2: Additional notes for defining classes of land cover

Broad land cover class	LCM2000 subclass	code	LCM2007 subclass	code
Deciduous	Broad-leaved / mixed woodland	1.1	Broadleaved woodland	1
Coniferous	Coniferous woodland	2.1	Coniferous woodland	2
Enclosed Farmland	Arable cereals<br>Arable horticulture<br>Arable non-rotational<br>Setaside grassland	4.1<br>4.2<br>4.3<br>5.2	Arable and Horticultural Land	3
Improved Grassland	Improved Grassland	5.1	Improved Grassland	4
Semi-natural Grass	Acid grassland<br>Neutral grassland<br>Calcareous grassland<br>Fen, marsh, swamp (rush pasture)	8.1<br>6.1<br>7.1<br>11.1	Acid Grassland (Bracken)<br>Neutral Grassland<br>Calcareous Grassland<br>Fen / swamp<br>Rough Grassland	8<br>6<br>7<br>9<br>5
Mountains, moors and heaths	Bog (deep peat)<br>Montane habitats<br>Inland bare ground<br>Dense dwarf shrub heath<br>Open dwarf shrub heath<br>Bracken	12.1<br>15.1<br>16.1<br>10.1<br>10.2<br>9.1	Bog<br>Montane habitats<br>Inland rock<br>Heather<br>Heather grassland	12<br>13<br>14<br>10<br>11
Coastal Margins	Saltmarsh<br>Littoral rock<br>Littoral sediment<br>Supra-littoral rock<br>Supra-littoral sediment	21.2<br>20.1<br>21.1<br>18.1<br>19.1	Saltmarsh<br>Littoral rock<br>Littoral sediment<br>Supra-littoral rock<br>Supra-littoral sediment	21<br>19<br>20<br>17<br>18
Freshwater, Wetlands	Water (inland)	13.1	Freshwater	16
Marine	Sea / Estuary	22.1	Saltwater	15
Urban and developed land	Continuous urban<br>Suburban / rural developed	17.2<br>17.1	Urban<br>Suburban	22<br>23

Note: LCM = Land Cover Maps compiled by UKCEH for the year's shown; drawn from (4) and (6)

1.3: A note on assumptions and limitations:

The assessment of outcomes for all three approaches follows consistent, standard cost-benefit analysis rules. This is appropriate as these are incorporated within the HM Treasury rules

guiding government appraisal and evaluation of policies (12). We therefore adopt the same rules as used by policymakers.

Cost benefit rules require that values should reflect underlying willingness to pay. For goods delivered in competitive markets (e.g. food or timber production) it is assumed that these are reflect in market prices. For non-market goods prices are unavailable and have to be directly estimated. As detailed subsequently, our recreation analysis employs random utility modelling to estimate values. The valuation of greenhouse gas emissions and carbon storage touches upon a substantial literature with the majority of academic studies focussing on marginal abatement costs while many policy analyses utilise officially sanctioned carbon prices. The NEV model is programmed to accept any of the above approaches but, given the policy focus of our study we adopt the latter approach to ensure consistency of application across all three approaches. As we argue elsewhere (14) we feel that the present understanding of the relationship between biodiversity and related ecosystem functioning (e.g. biogeochemical cycles such as the water or carbon cycles), together with debates regarding non-use values mean that there is not an adequate basis for economic valuation. Consequently we argue that the biodiversity impacts of any investment or public funding should be assessed and a net-gain constraint applied to ensure that wild species are both incorporated into decision making and biodiversity losses reversed as per numerous national and international agreements.

Other assumptions used in the present paper include the incorporation of climate change through the UK NEA low emission scenario (13) and the adopting of the UK public spending discount rate throughout (12). Note that both of these assumptions can be readily altered within the NEV decision support system.

While the NEV system explicitly incorporates food, greenhouse gases, biodiversity and recreation into its decision support there are other consequences of land use change which are currently omitted. At the time of writing the effects of land use change upon the water environment (including water quality, quantity and flood risk) is under active incorporation within NEV alongside considerations of risk, uncertainty and their management through portfolio analysis while assessment of the dynamic interactions between housing demand and land use change is under investigation.

While the NEV system explicitly incorporates food, greenhouse gases, biodiversity and recreation into its decision support there are other consequences of land use change which are currently omitted. At the time of writing the effects of land use change upon the water environment (including water quality, quantity and flood risk) is under active incorporation within NEV alongside considerations of risk, uncertainty and their management through portfolio analysis while assessment of the dynamic interactions between housing demand and land use change is under investigation. Other values such as those concerning cultural and heritage benefits are the subject of primary research.

Annex A1: Land use variables

new2kid	= unique identifier for 2km cell
Easting	= British National Grid Easting (x coordinate) for centre of 2km cell
Northing	= British National Grid Northing (y coordinate) for centre of 2km cell
Area_ha	= Area of a 2km by 2km cell in hectares (400ha for all - i.e. includes non-land in some coastal areas)

The following are % of cell area:

COAST	= % area coverage by coastal margins
FWATER	= % area coverage by freshwater
MARINE	= % area coverage by sea and estuary
URBAN	= % area coverage by urban and other developed land
PERMG	= % area coverage by permanent grassland (>5yrs)
TEMPG	= % area coverage by temporary grassland (<5yrs)
RGRAZ	= % area coverage by rough grazing
GRSNFRM	= % area coverage by semi-natural grass, mountains, moors and heaths where NOT used for farming
FWOOD	= % area coverage by farm woodland
NFWOOD	= % area coverage by woodland NOT used for farming
WHEAT	= % area coverage by wheat
WBARLEY	= % area coverage by winter barley (England and Scotland)
SBARLEY	= % area coverage by spring barley (England and Scotland)
OTHCER	= % area coverage by other cereals (including oats and other for combining)
POTS	= % area coverage by potatoes
WOSR	= % area coverage by winter oilseed rape (where available)
SOSR	= % area coverage by spring oilseed rape (where available)
MAIZE	= % area coverage by maize (Scotland 2004 this is within 'othcrps')
HORT	= % area coverage by total horticulture
TBARLEY	= % area coverage by total barley (Wales only)
TOSR	= % area coverage by total oilseed rape (where seasonal data unavailable)
OTHFRM	= % area coverage by other farmland (roads, buildings, yards, ponds and setaside)
SUGARBEET	= % area coverage by sugar beet not for stockfeed (England only)
OTHCRPS_N	= % area coverage by other crops (includes bare fallow; includes OSR for Wales; includes maize for Scotland 2004)
OCEAN	= % area that is not covered by any 1 km cells and is therefore given 'ocean' by default

2. The land use and livestock module

2.1. Summary

This section presents a spatially explicit, structural econometric model of UK land use. As urban areas are generally unavailable for land use change to non-urban usage, and forests are both protected from conversion to non-woodland use and set to rise in area under net-zero policy, the land use module is effectively a model of agricultural land use embracing the three quarters of the UK which is farmed (2).

The agricultural land use model embraces the market, policy and environmental drivers of land use decisions related to crop and livestock production, and estimates resultant land use and livestock intensity. The structural nature of the model is important as this guides the model to capture those factors behind land use decisions that are likely to provide a firm basis for predicting responses to future change. So, for example, the availability of government subsidies and changes in the climate are likely to continue to affect future decision making, and the model shows the decision maker the likely magnitude of that responsiveness to change.

The model is developed on long term, spatially disaggregated data and is exhaustively validated using out-of-sample, actual versus predicted testing (15). This provides a robust understanding of the land use response to changes in environmental, economic and policy drivers.

The relationship between food production and food security is a complex and multi-dimensional (16) which in the aggregate concerns issues of food availability, accessibility, utilization and stability (17). Given this, quantity-based metrics, particularly those concerned with land use and agricultural production, can be misleading indicators of food security. For example, tonnage of output or calorific measures combines a highly diverse set of food types of different nutritional value. Reservations regarding quantity measures are particularly relevant in a highly developed country such as the UK where, with average per capita production of calories at over 3,300 kcal per day (18; 19), food output considerably exceeds that required for healthy consumption levels (2,000-2,500 kcal per day (20). In the context of our study, assessment of food security is simplified by the fact that we are looking at changes in food production, rather than total output. To avoid the problems of quantification and allow comparison with other potential land uses we assess changes in food security in terms of the change in the economic value of agricultural output arising under each option. This can then be directly compared with other potential land use outputs, such as timber, greenhouse gas storage and recreation which are also assessed in terms of economic values.

2.2. Objectives

- To model historic land use data and agricultural profitability and understand the relationship between the two
- To relate this land use and profitability data to environmental, economic and policy determinants, thereby understanding how changes in those drivers affect land use
- To conduct this analysis so as to project, at a 2km (400ha) grid square resolution for the entirety of Great Britain:
    - shares (down to fractions of a hectare) within each grid cell of the seven major agricultural land uses: cereals; oilseed rape; root crops; temporary grassland; permanent grassland; rough grazing; and other agricultural land. Note that while

each share is expressed at a resolution down to fractions of a hectare the data reference is not available at sub-2km grid square resolution such that we might know that 42.2ha of the 400ha grid is oilseed rape, we do not know where within that grid square this crop is located.

and;

- stocking intensities for the major livestock types: dairy cows, beef cows and sheep, again known down to 2km (400ha) grid square resolution;

- To use this analysis to understand the influence of expected climate change upon land use and profitability;
- To feed that analysis of future land use into life cycle assessment of agriculture related greenhouse gas (GHG) storage and emissions (see subsequent discussion).
- To conduct the above analyses in ways that readily allow changes in forest yield and profitability (see below) to be incorporated within estimates of future land use and related combined (agricultural and forest) GHG emissions and storage.

2.3. Methodology

Figure 1 provides a diagram representing the overall structure of our modelling framework. Here we present just an overview of our approach, all technical details are presented in (21). While some of these models are estimated on June Agricultural Census (JAC) data and some other on Farm Business Survey (FBS) data, all the models are used for predictions at the 2x2 km level, matching the resolution of the other NEV modules.

At the first step, the model separates all the available agricultural land (with the exception of farmed woodland, which is not modelled here) between grassland and arable. This model is estimated as a quasi-maximum likelihood (QML) Logit model on 2x2 km grid JAC data from 1972 to 2010 (11 unevenly spaced years) coupled with climate, environmental, price and policy information described above.

In the second step, arable land use is allocated among wheat, barley, oilseed rape, root crops and other land (including other arable, fallow, uncropped etc.). Grassland land use, on the other hand, is allocated among temporary grassland, permanent grassland and rough grazing. Both models are derived from a normalized quadratic multi-output profit function and estimated using use the multivariate Tobit quasi-maximum likelihood (QML) approach developed by (15). However, the arable model is estimated on 2005-2011 FBS data, which contains rich information on prices, while the grassland model is based on JAC data, in order to include also data for Wales and Scotland, which host a significant share of UK grassland and rough grazing.

The livestock models for dairy cattle, beef cattle and sheep are based on the same multi-output profit function, but uses a QML Poisson estimator in place of the Tobit one. We compared both approaches and the Poisson provides a better fit and much lower prediction errors. Like the grassland equations, also the livestock models are ì estimated on JAC data. The reason is that livestock data in the FBS is too volatile and not necessarily correlated with prices and livestock heads. For example, in one year the revenues from cattle can be high and the number of cattle low because the farmer sold many livestock at the beginning of the year without replacing them, and vice versa, revenues can be even negative if the farmer has bought young cattle to be raised and sold in the following years for profit. In addition, there is no information on the

weight of the animals, which strongly influence prices. For this reason, it is not possible to calculate meaningful farm-level price indexes with this data. JAC data coupled with regional price indexes smooth away this confounding source of variability, thereby allowing the estimation of our livestock and grassland models.

Figure 1: Modelling approach

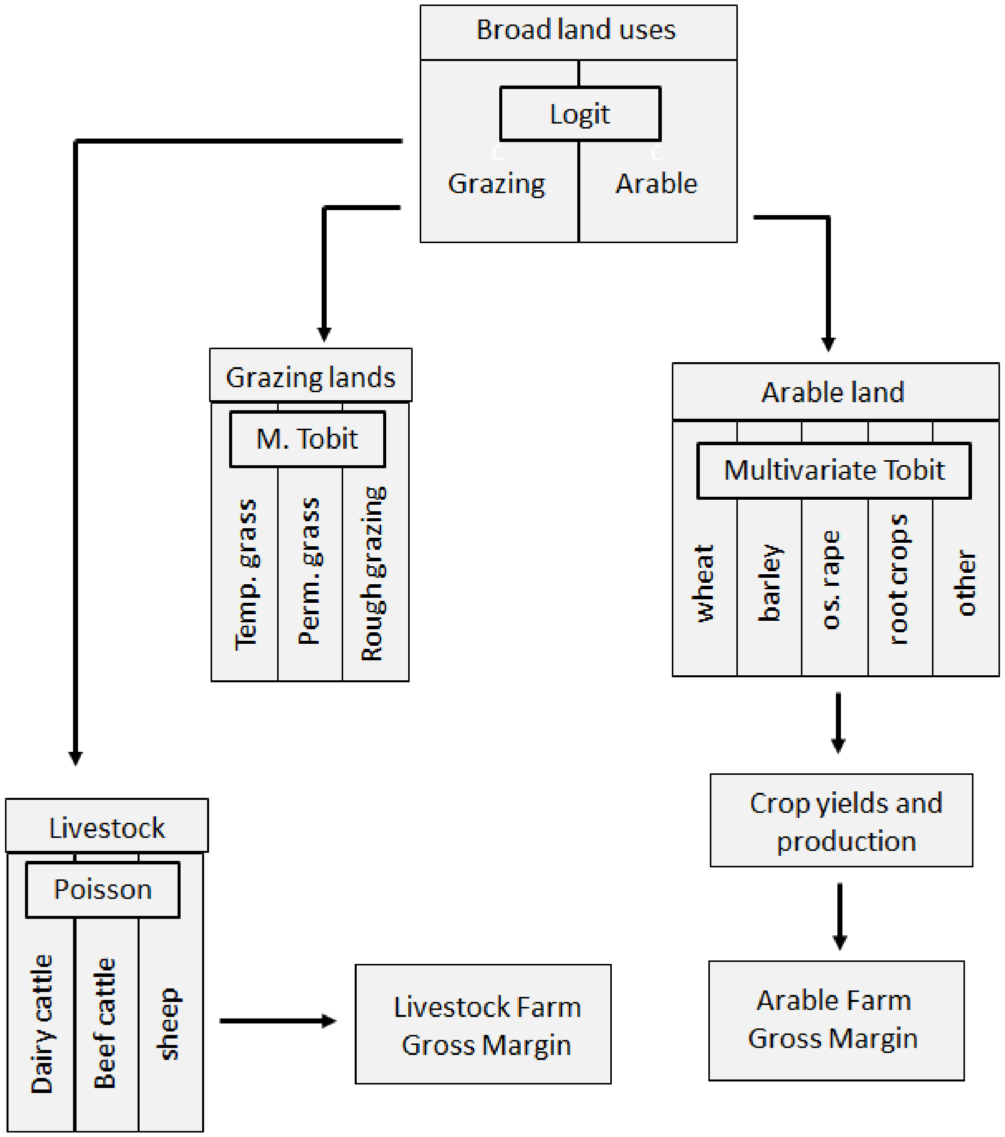

In the final step, we calculate gross margin using some simple assumptions. For arable farms, we use information on yield and prices to calculate revenues. We then assume that gross margin corresponds to 45% of revenues, a proportion that has been surprisingly stable in the past years. For example, during the 10 years between 2008 and 2018 it always remained between 42% and 45% (22). Here for simplicity we obtain FGMs by multiplying revenues by 0.45. Regarding livestock, we also assume gross margins to be related to output prices and historical information.

2.7. Limitations of our approach

Several caveats need to be considered when considering the results produced by our agricultural modelling approach.

First, this framework is essentially static and looks at equilibrium, long-run relations. While this is an essential feature for examining long-term impacts such as climate change, it does not investigate inter-temporal aspects of agricultural production decisions. For instance, we assume equilibrium in the land market with land shadow prices equal across all land uses. However, in the short-run other factors such as levels of existing capital (e.g. buildings) or conversion costs could bring disequilibrium in the land market.

Second, the issue of land tenure, which can be important in shaping agricultural land decisions, is not addressed in this work. For example, traditional agricultural tenancies guarantee lifetime security of tenure in most circumstances, with the considerable prospect of succession for two more generations. Such tenancies traditionally include a clause restricting the land to agricultural use, reserving existing trees to the landlord, and preventing tree planting on any scale by the tenant farmer.

Third, we assume that farmers are risk neutral and profit maximisers. While other factors besides mere profit maximization can influence farmers' decisions and previous research as shown that farmers can exhibit a significant level of risk aversion, nevertheless the strong, out of sample validity of the model in predicting actual land use suggests that, at least over the medium to long term, such assumptions yield empirically defensible models.

Fourth, the research here focuses on the impact of changes in temperature and precipitation on land use decisions, but does not account for other factors which might be affected by climate change. For example, increased CO_2 fertilization could improve crop yields, however there may be a quantity versus quality trade-off as these could be offset by declining nutritional value. Further potential effects of climate change include impacts on pollinators and the transmigration of new crop pests and diseases. Finally, although we considered the impact of changing average temperature and precipitation, we did not consider potentially significant impact extreme events. Nevertheless, our related research suggests that this is a defensible approach over the period under analysis (23).

Fifth, we do not account for the potential introduction of novel farming practices or technologies such as new crops.

3. The timber module: forest growth and the financial returns from timber.
3.1. Introduction

The timber module was developed by Robert Matthews and colleagues at Forest Research (UK) and provides analysis of tree growth and the financial returns from timber. This was extended to consider production under current and future climates. The module consists of two distinct elements:

(i) a model, with flexible functional forms, of forestry growth measured in m^3/ha/annum (with the peak timber growth rate attained by a stand of trees over its lifetime being known as its Yield Class (YC); see discussion in (24) incorporating the impacts of variation in the physical environment (e.g. soil characteristics) including climate variables and climate change;

(ii) the relationship between YC and the financial returns from timber.

3.2. Objectives

- To model variation in growth rates and timber YC for representative conifer and broadleaf tree species and their response to the full range of variation in physical environmental conditions across Great Britain.
- To incorporate into this analysis the influence of climate change upon growth rates and timber yields.
- To provide estimates of future tree growth and timber yield to feed into life cycle assessment of forest-related greenhouse gas (GHG) storage and emissions (see subsequent discussions).
- To predict timber costs, revenues and hence profitability for different tree species across locations, climate scenarios and a common silvicultural management regime.
- To feed profitability assessments into estimates of how land owners will react to changes in the financial value of alternative land uses and hence expected land use change.

3.3. Yield class and the financial returns from timber.

3.3.1. Yield class and the financial returns from timber: Data

In determining the suitability of sites for forest growth we rely on several databases derived from modelling combined with advice from UK Forestry Commission (FC) experts. Site specific expected forest growth is established through use of the Ecological Site Classification (ESC) model (25), a well-established decision model originally developed by (26) and based on a synthesis of multi-criteria analysis (27) and fuzzy-set theory (28). A schematic overview of the model is presented in Figure 3.1.

Figure 3.1: Schematic diagram of the ESC model.

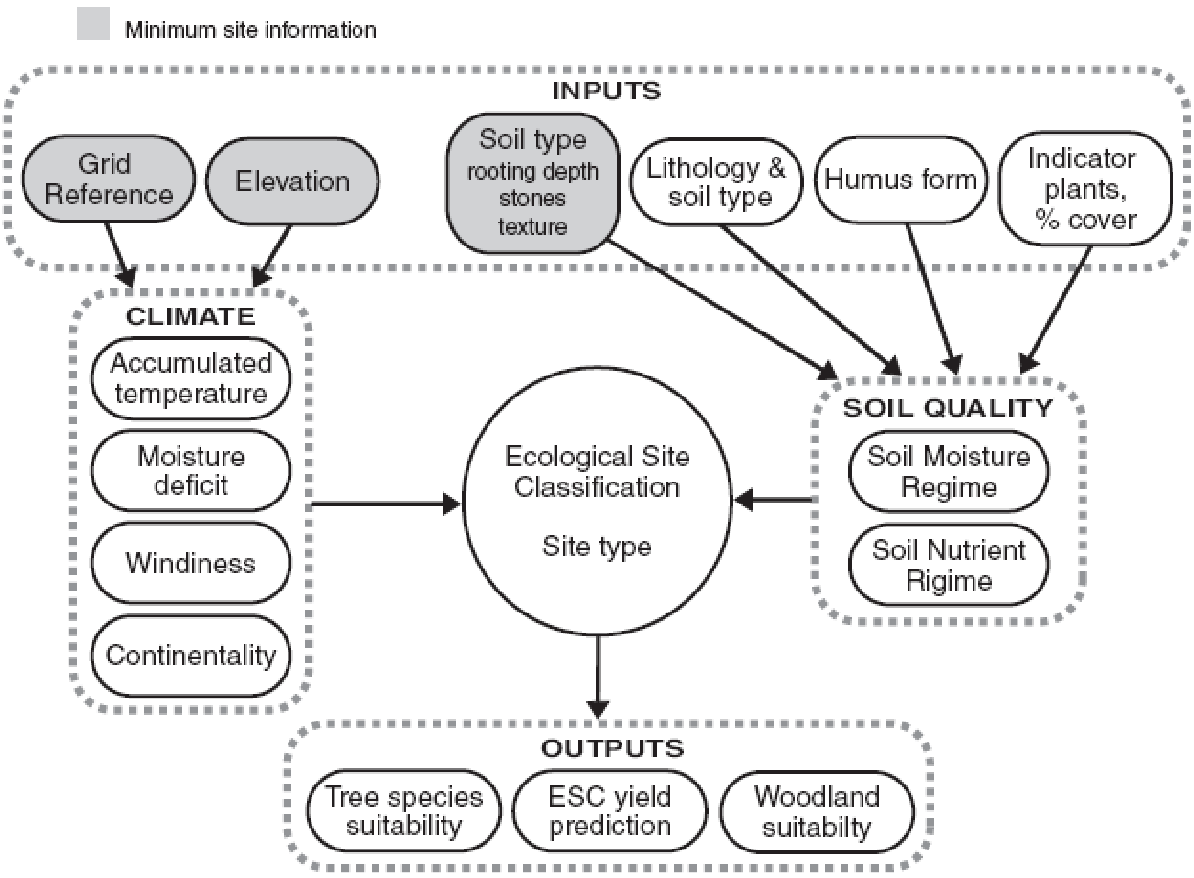

Source: (29)

The ESC model provides an analysis of tree growth rates and timber yield which is sensitive to the suitability of land (in terms of soil, moisture, elevation, temperature, etc.) and incorporates the judgment of experts who assign characteristics into two macro-classes: climate and soil (25). Each macro-class is further organised into sub-classes (e.g. accumulated temperature or soil moisture regime). One output of the model is predicted YC (in m^3/ha/yr.) for each GB 250m grid cell[5]. This output resolution was converted to the 2km grid system used for the wider analysis. Table 3.1 provides a quantitative summary of data for the two representative species (30) considered in this analysis: Sitka Spruce (SS) for coniferous and Pedunculate Oak (POK) for broadleaf.

Table 3.1: Yield class (YC) characteristics across GB.

Tree species	Mean YC	st.dev	Min	Max
Sitka Spruce (SS)	13.23	3.81	0	21
Pedunculate Oak (POK)	3.82	1.95	0	8

YC = the peak timber growth rate attained by a stand of trees over its lifetime; measured in m^3/ha/annum
Source: ESC (25)

[5] ESC estimates YC as a continuous variable which is assumed to be zero where soil and climatic factors are unsuitable for planting; such as on bare rock or in urban areas. Note that it is conventional for forestry studies for average YC values for a given stand to be rounded to the nearest even number (31).

3.3.2. Yield class and the financial returns from timber: Methodology

The financial returns from timber production are obtained by multiplying tree timber volume by corresponding market price and making allowance for all relevant costs.

To obtain tree volumes the YC values obtained from ESC were fed into the CARBINE model (32), which adapts the site specific estimates of tree volume to a variety of management regimes. For the purposes of modelling in NEV in both this section and in the forest greenhouse gas model we consider a 'thinning and felling' management regime. As Figure 3.2 illustrates, the rate of growth and volume of timber output from SS is both faster and more plentiful than that of POK.

Figure 3.2: Harvested timber volumes over multiple rotations: Sitka Spruce (yield class 14) and Pedunculate Oak (yield class 4).

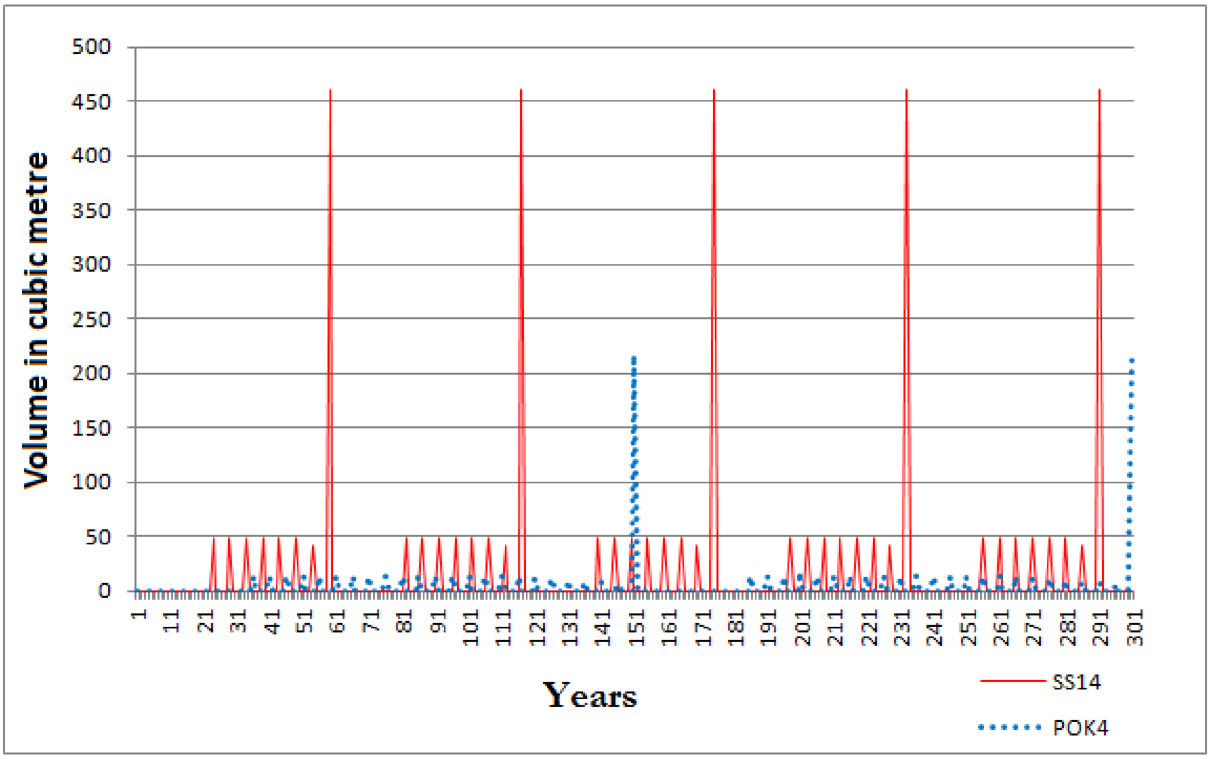

Note: The figure shows the volume of timber harvested from thinnings and felling over a three hundred year period. A site producing SS at YC14 might only see POK growing at YC4. This results in five rotations of SS occurring in the time taken to produce just two rotations of POK.

Source: Derived from the CARBINE model (32)

Tree volume taken from the CARBINE model is then combined with the FC Forest Investment Appraisal Package (FIAP) (33) to calculate the financial performance of forests through the comparison of revenues based on current timber prices and management costs. FIAP allows the analyst to combine price per m^3 for SS and POK with average management costs (for activities such as mounding, planting, staking, insurance, drainage, weeding, spraying etc.,) under a variety of silvicultural systems (34).

While the vast majority of goods traded in the world have a constant unit price this is not the same for trees and timber. For any given species of tree the price per tonne of timber increases with the volume of that tree. Put simply, a tonne of small stem trees is worth far less than a solid tonne of timber cut from a single tree. This is because while the latter can be used to produce a huge variety of high value products (e.g. flooring, roofing and manufacturing), the former is only suitable for say fencing, fuel or pulp. The unit price per tonne therefore rises rapidly as the volume of the trees concerned increases. For conifers this relationship hits a constant value once stems are large enough to yield for construction materials. For hardwoods, the premium on the largest stems still persists (e.g. for the highest quality products) a decline in the rate of increase in unit price is still noticeable.

The resulting price-size curves are monitored by the FC over time and are expected to remain constant in real terms throughout the period of the analysis (35). These curves are illustrated in Figure 3.3.

Figure 3.3: Price-Size Curves for Sitka Spruce and Pedunculate Oak.

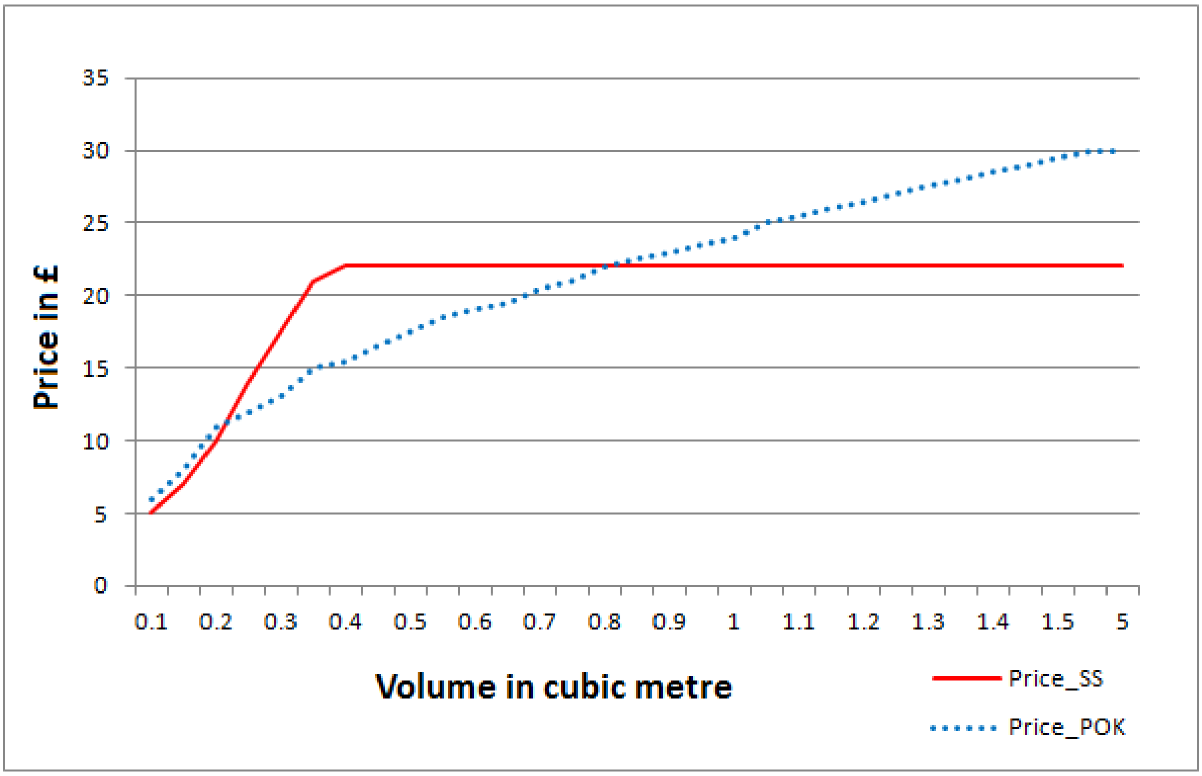

Note: Comparison of price-size curves (price by volume per cubic metre) for Pedunculate Oak and Sitka Spruce.

Source: Derived from (33) using contemporary price data

Differences between species are also reflected in management costs. For example, on average managements costs for POK YC4 in the first 10 years are £560/ha whereas they are only £230/ha for SS YC14. Relevant management costs refer only to variable costs and exclude fixed costs such as fencing, consultancy advice, etc., which are expected to be significant only in the early years of land use conversion.

In addition to timber values, analysis of the private financial value of woodland includes forestry grant payments. However, in keeping with the shadow pricing approach (36, 37)

adopted for the treatment of agriculture, we exclude forestry grant schemes from our assessments of the social value of land use conversions on the grounds that these represent transfer payments within society.

Financial returns are calculated by first converting all current and future revenues to their present day equivalents using the official social discount rate of 3.5% (38) and then summing these to calculate the Net Present Value (NPV; 39)[6] of timber production. This overall value can then be related to an annual equivalent (annuity) using standard formulae (35). Results are reported in Table 3.2 for each species and YC.

Table 3.2: NPV and annuity values for one hectare Sitka Spruce (SS) and Pedunculate Oak (POK).

Species	Yield Class	Net Present Value (£/ha)	Annuity (£/ha/yr.)
SS	6	-2262	-84
SS	8	-1865	-71
SS	10	-1336	-51
SS	12	-813	-31
SS	14	-243	-10
SS	16	299	12
SS	18	884	35
SS	20	1278	53
POK	2	-6485	-221
POK	4	-6340	-218
POK	6	-6159	-209
POK	8	-5750	-196

Note: Comparison of current expected profitability of Sitka Spruce and Pedunculate Oak under thinning and felling management regime and a constant social discount rate of 3.5%.

Table 3.2 shows that, with the exception of high yielding SS forests, the financial returns to timber production in the UK are negative. Such poor financial performance explains the low prevalence of commercial woodland across the majority of Great Britain, a result which is reinforced by the comparatively high rates of subsidy for farming across the country. However, this calculation ignores the other social benefits, such as carbon sequestration and recreation considered elsewhere in this analysis. It also ignores the impact of climate change upon tree growth and timber yield, to which we now turn.

3.4. Modelling climate change impacts on forest growth and timber yield

[6] The net present value (NPV) of an investment project is found by summing all the present and discounted future benefits and costs of that project. If that assessment includes absolutely all benefits and costs (including the opportunity costs of alternative investments), and allows for risk, then a positive NPV indicates that it is worthwhile investing in that project. Note that the assessment given in Table 2.2 only includes private timber returns. It ignores capital gains (increases in land value) and all non-market benefits, both those accruing to the private owner (including the status benefits of land ownership, hunting, etc.) and those passing to wider society and therefore cannot be considered exhaustive. Adding in the other benefits and costs assessed elsewhere in this analysis is an essential part of delivering an adequate assessment.

Historically, forests have been fairly resilient to the effects of short run variation in weather patterns. However, climate change research indicates that, across the UK, temperatures are set to increase, especially in summer, while precipitation will increase in winter but reduce in summer. The impact of these changes are likely to vary across species (40; 41; 42), effects which need to be incorporated into our understanding of forest growth as they are liable to affect yield class. Furthermore, these effects are predicted to be locationally non-uniform requiring spatial analysis.

3.4.1. Data

While the ESC model (25) provides high quality estimates of yield class, the raw outputs available to this research lack sensitivity to climate change. To address this we develop a new model of tree growth rates linking the spatially and species sensitive yield data underpinning ESC to local environment and climatic factors taken from the climate, soil and terrain data described at the start of this Supplementary Information. This is then used to conduct a cross-sectional multivariate analysis of the co-dependencies of variables. Factors drawn from the dataset have been selected to be as similar as possible to the input variables used in ESC. The key variables in the model are:

- Mean temperature and precipitation during the UK growing season (April to September) over the period 1961 to 1990.
- Average slope and elevation of the cell, which are further determinants of the YC.
- Easting and Northing. These variables are ancillary to the description of the YC changes but we expect that they will capture spatial correlation in other explanatory factors not explicitly modelled here.
- Soil characteristics defined in Table 1.1. are transformed into a set of binary variables:
  - The dominant annual soil water regime, which takes value 1 if the soil is defined as not wet (e.g. water-table is not wet within 80cm of the soil surface for 3 or more months per annum); 0 otherwise.
  - The pH variable, which takes value 1 if pH > 5.5, namely if the soil is a rich or very rich soil type (26); 0 otherwise.
  - The water capacity variable, taking value 1 if water in soil > 75mm/m; 0 otherwise.
  - The carbon in soil variable, describing soil health; the variable takes value 1 when soils are healthy, namely where the percentage of organic carbon in top and sub-soil is within a range: > 1.2% and < 25%; 0 otherwise.

3.4.2. Methodology

To model the complex non-linear relationships between YC, the physical characteristics of the local planting area and the climate we rely on the highly flexible semi-parametric regression approach provided by the generalized additive modelling method developed by (43). This is preferred over simple linear regression or other parametric specifications as it allows the modelling to adapt to non-linearities in the data such as those between tree growth and variations in the climate.

This modelling approach proceeds through two steps. In the first we allow the relationship between tree growth and the two key elements of climate, temperature and rainfall, to take a

data-determined non-linear form as specified in Equation 3.1. Here we specify functions allowing the relationships of these two variables to growth to follow a non-linear path beyond some data-determined threshold level; temperature K and rainfall J. These threshold levels are set by set by maximising the fit of the model for different levels of K and J.

Equation 3.1:

$$Temp_1 = \begin{cases} 0 & \text{for Temperature} \leq K \\ (\mathrm{MT}_i - \mathrm{K}) & \text{for Temperature} > K \end{cases}$$

$$Temp_2 = \mathrm{MT}_i$$

$$Rain_1 = \begin{cases} 0 & \text{for Rainfall} \leq J \\ (\mathrm{MP}_i - \mathrm{J}) & \text{for Rainfall} > J \end{cases}$$

$$Rain_2 = \mathrm{MP}_i$$

Where: MT_i is the historic mean monthly air temperature (°C) for the growing season (April to September) averaged for 1961-1990 for each 2km grid square *i* across GB and; MP_i is the historic total rainfall (mm of precipitation) for the same growing season and averaged over the same time period for each grid square interpolated from 5 km grid baseline data for UKCP09 held by the Met Office[7].

The output of this first step is the values for the two thresholds. The temperature threshold (K) differs between the species, being 12°C for SS and 9°C for POK. For rainfall, the threshold (J) is 400mm for both species. These thresholds identify the values above which the effect of temperature and rainfall on yield class is no longer linear.

The second step models tree growth (YC) within highly flexible semi-parametric regression functions for SS and POK. This defines a set of non-parametric, smooth functions s(.) of variables which, though exerting considerable influence upon growth, are not going to change into the future and therefore simply need to be controlled for in any predictions drawn from the model. The variables modelled using such smooth functions are: *slope*, *elevation*, and *Easting* and *Northing*. This approach removes a very high degree of non-focal variation from the analysis which might otherwise unduly affect estimates of the focal fixed factors which are estimated using parametric specifications to permit robust prediction. The key variables of interest here are those connected to climate and therefore predicted to change throughout the remainder of this century. These are the temperature and rainfall variables non-linearly defined in Equation 2.1. In addition to these we also parametrically model certain other variables as recommended by our Forestry Commission partners. The final model is specified as Equation 3.2.

Equation 3.2:

$$\begin{aligned} YC = {} & \alpha + \beta_1 s(easting, northing) + \beta_2 s(slope) + \beta_3 s(elevation) \\ & + \beta_4 Wr + \beta_5 Wc + \beta_6 pH + \beta_7 Carbon \\ & + \beta_8 Temp_1 + \beta_9 Temp_2 + \beta_{10} Rain_1 + \beta_{11} Rain_2 \end{aligned}$$

[7] Where necessary (e.g. where boundaries of grids or coastal cell cause uncertainties) the value of the adjacent cell has been used.

$$+\beta_{12}Temp_1Rain_1 + \beta_{13}Temp_1Rain_2 + \beta_{14}Temp_2Rain_1 + \beta_{15}Temp_2Rain_2 + \varepsilon$$

where all variables are indexed to individual 2km grid squares i; the variables in the smooth functions, s(.) are: *easting* and *northing*, *slope* and *elevation*; the non-focal parametric dummy variables are: *Wr* the water regime, *Wc* water capacity, *pH* soil-pH level, and *carbon* is carbon in soil; and the focal parametric step functions are temperature and rainfall variables non-linearly defined in Equation 3.1 ($Temp_1$, $Temp_2$, $Rain_1$ and $Rain_2$) together with all interaction variables (e.g. $Temp_1 * Rain_2$); and ε is a normally distributed error term.

3.4.3. Results: Modelling tree growth

Equation 3.2 was estimated separately for SS and POK providing the results given in Table 3.3.

Table 3.3: Predicted timber yield class (YC) for Sitka Spruce (SS) and Pedunculate Oak (POK) as a function of cell characteristics for all 2km GB grid cells

Variable	Description	SS	POK
		Flexible functions	
		edf (st.err)	*edf (st.err)*
s (easting, northing)	Ancillary variable (captures non-focal local variation)	28.80*** (29.00)	28.91*** (29.0)
s (slope)	Average slope of the cell	8.40*** (8.90)	8.38*** (8.90)
s (elevation)	Average elevation of the cell	8.88*** (9.00)	8.44*** (8.91)
		Fixed factors	
		Coeff (st.err)	*Coeff (st.err)*
Wr (Water regime; dummy var)	Annual dominant soil water regime. 1= if water-table is not wet (within 80cm of surface for 3 or more months pa.); 0=otherwise	0.0589*** (0.0108)	0.1237*** (0.0061)
Wc (Water capacity: dummy var)	Water storage capacity expressed as millimetres per metre (mm/m): 1= water in soil > 75mm/m 0=otherwise	-0.0284* (0.0131)	0.0610*** (0.0076)
pH (Dummy variable)	Soil Health: 1= Non-acid soils (pH>5.5); 0= otherwise	0.0306 (0.0160)	0.2837*** (0.0089)
Carbon	Carbon in soil (% of organic carbon in top soil). 1= if between 1.2% & 25%; 0=otherwise	-0.088*** (0.0114)	-0.0291*** (0.0070)
$Temp_1$	Temperature threshold: >K K=12°C for SS. K=9 °C for POK	4.669*** (0.293)	0.6330*** (0.0630)
$Temp_2$	Temperature (°C)	-4.935*** (0.007)	0.1836*** (0.0186)
$Rain_1$	Rainfall threshold: > J J=400mm for SS and POK	0.1358*** (0.0065)	-0.0542*** (0.0038)
$Rain_2$	Average rainfall in the growing season (mm)	-0.137*** (0.0065)	-0.1370*** (0.0065)
$Temp_1Rain_1$	(Temperature threshold: > K) *(Rainfall threshold >J)	0.0194*** (0.0009)	-0.0039*** (0.0005)
$Temp_2Rain_1$	(Temperature) * (Rainfall threshold: > J)	-0.0132*** (0.001)	0.0055*** (0.00045)

$Temp_1Rain_2$	(Temperature threshold: >K)*(Rainfall)	-0.0163***<br>(0.001)	0.0013***<br>(0.0003)
$Temp_2Rain_2$	(Temperature) * (Rainfall)	0.0130***<br>(0.006)	-0.0033***<br>(0.0003)
Constant (α)		66.36***<br>(2.60)	-0.0567***<br>(0.0090)
N	Number of cells	56,366	50,766
Log-likelihood		-42264	-42105
R^2Adj		0.93	0.89

Notes: Significance p-value: *p<0.05; **p<0.01; ***p<0.001.
The semi-parametric specification enables the distribution of continuous explanatory variables to be kept flexible, changing in accordance with the data. This approach compares favourably with parametric approaches as it allows estimates of the degree of non-linearity without any need for further assumptions. In the upper part of the table the effective degree of freedom (edf) reports the estimated level of non-linearity for the slope, elevation and easting and northing variables. In the second half of the table, coefficients of linear parameters describe their effect on YC.

The results presented in Table 3.3 are given in two parts reflecting the functional forms adopted. The upper part of Table 3.3 reports the effective degree of freedom (edf) of the smooth functions which explain the estimated level of non-linearity for the easting and northing, slope, and elevation variables. Below this in the remainder of the table we report estimated coefficients for the linear parameters such as the non-focal dummy variables; and the focal (climate related) rainfall and temperature variables expressed as step-functions.

All the variables modelled as smooth function are highly non-linear, in fact the higher the *edf*, the more non-linear the estimate *s*(.). So, while an *edf* equal to one means that the best approximation for that variable is linear, in our data we find that all variables are better represented by non-linear functions.

The parametric variables are all highly significant and with the expected sign. The pH and water regime dummies are positively related to YC for both species, indicating that yield rises in response to increases in these factors. Conversely, water capacity is negative for SS and positive for POK, indicating that a rise in water capacity leads to a fall in the yield for SS, but a rise in POK YC, a finding which is consistent with other work in the field (40). This and other climate related relationships affecting growth become increasingly important in a warming world, as discussed in greater detail below.

The overall explanatory power of the YC models for SS and POK is highly satisfactory. The SS model explains 92% of observed variation in YC with an average Mean Square Error (MSE) of 1.01 (median 0.3) implying that predicted values will be generally very well-determined. The POK model also performs well explaining 89% of YC variation with a mean MSE of 0.31 (median 0.11).

3.4.4. Results: Predicting tree growth under climate change

The YC models reported in Table 3.3 provides a rich understanding of the responsiveness of each species to variation in temperature and rainfall and hence can be used to predict the impact of climate change on both SS and POK using the Met Office UK Probabilistic Projections of

Climate Change over Land[8], medium emissions model, as per Table 0.1. These provide probabilistic, monthly average, climate projections for the UK out to 2079.

For both tree species the relationship between temperature and growth is non-linear. For SS we find that the temperature effect is positive under the species-specific threshold of 12°C but negative above, indicating that the species performs less well at higher temperatures. This finding reflects the fact that this species originates from the cool conditions of the Canadian west coast and southern Alaska, suggesting that SS is vulnerable to the contemporary central European conditions which climate change is bringing to the UK. In contrast, the temperature effect for POK while also non-linear, is positive both below and above its 9°C threshold.

As for is the case for agriculture (44), the interaction between rainfall and temperature is significant for both tree species. While SS can cope well with variations in rainfall at lower temperatures, as the climate warms the species becomes increasingly dependent upon high rainfall in order to maintain growth rates. In contrast, within the ranges of both current and expected future UK climate, POK is resistant to low rainfall and responds positively to higher temperatures making it relatively robust against climate change when compared to SS.

Findings from this analysis are fed into the analysis of timber values as outlined previously. This is then fed into the NEV optimisation model where these benefits are compared with the net benefits of alternative land use.

3.5. Conclusion

The results presented detail the non-linear relationships between temperature and precipitation and tree growth for two key representative species. Growth rates are expected to occur under climate change though these vary across time, locations and species. The modelling system captures these changes and relates them to consequence response in timber production and value. As discussed subsequently, this also translates into impacts on the carbon storage capacities of these different tree species.

[8] The Met Office UK Probabilistic Projections of Climate Change over Land provides climate estimates (including temperature and rainfall) for a 25km grid (2028 cells) across the UK at monthly timesteps out to 2079. Bilinear interpolation was then used to relate this our 2 km grid. Further detail provided at: https://www.metoffice.gov.uk/research/approach/collaboration/ukcp/science/probabilistic-projections.

Annex A3: Interaction effects and non-linearity between climate variables and timber productivity

The semi-parametric model with all continuous variables as smooth functions (*s*(.)) describes significant interaction effects between mean temperature and precipitation for both Sitka Spruce (Figure A3.1) and Pedunculate Oak (Figure A3.3). The relationship between climatic variables and Yield Class is non-linear and single smooth functions depict the expected non-linearity (Figures A3.2 for SS and A3.4 for POK). Building on this evidence from several semi-parametric models we define the model and step-functions reported in earlier in this section.

Figure A3.1. Sitka Spruce: smooth function for both temperature and precipitation variables

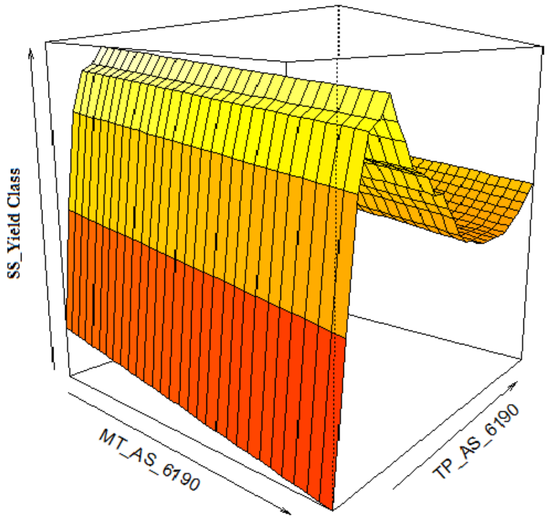

Note: Shows the interaction effect of temperature and precipitation on SS productivity

Figure A3.2. Sitka Spruce: single smooth function for temperature and precipitation

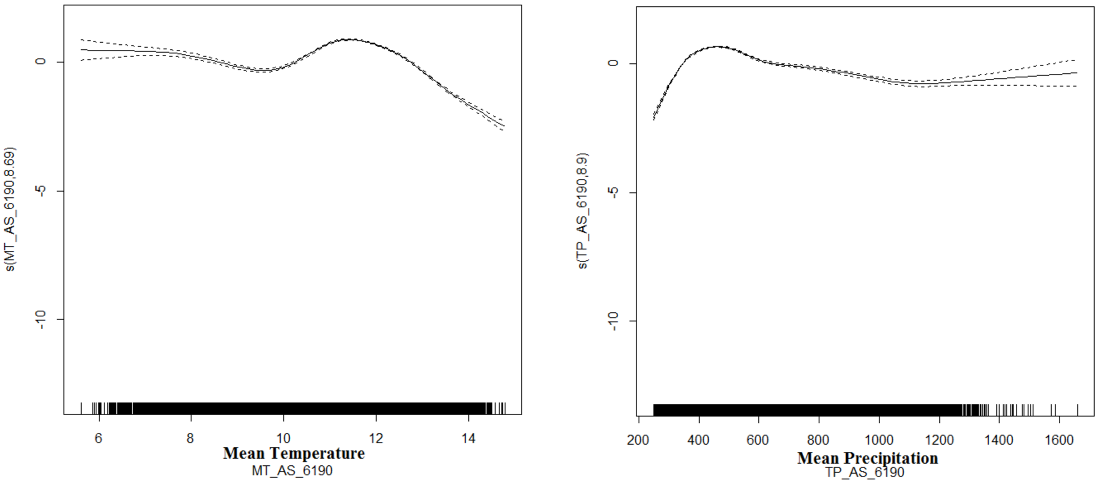

Note: The figure shows the non-linear function for temperature and precipitation. Both graphs have been used for the definition of the step-functions in the model of forest growth (Equation 2.2)

Figure A3.3. Pedunculate Oak: smooth function for temperature and precipitation variables

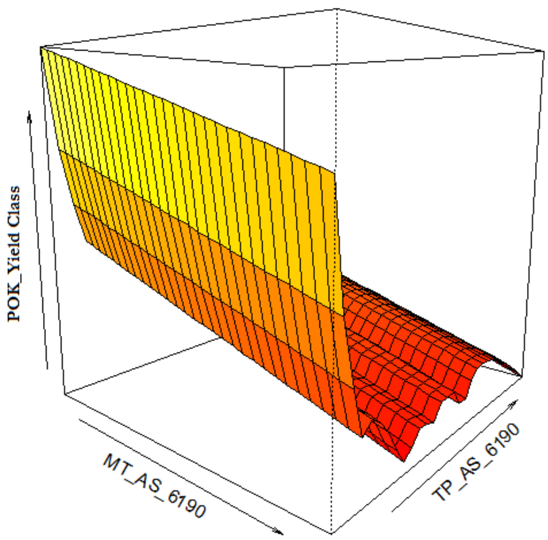

Shows the interaction effect of temperature and precipitation on POK productivity.

Figure A3.4. Pedunculate Oak: single smooth function for temperature and precipitation

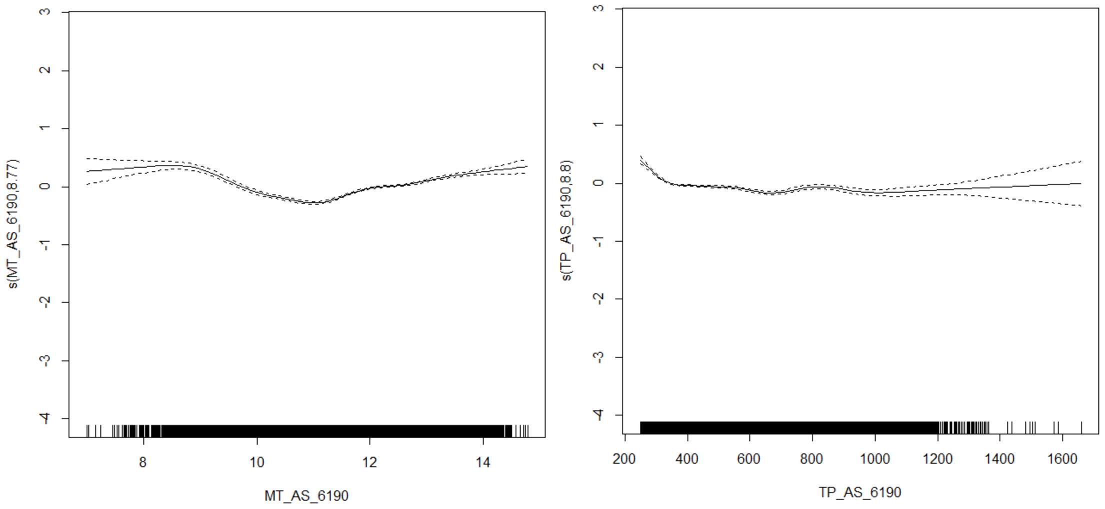

Note: Shows the non-linear function for temperature and precipitation. Both graphs have been used for the definition of the step-functions.

4: Agricultural greenhouse gas module
4.1. Introduction

This section discusses the spatially and temporally explicit modelling of greenhouse gas (GHG) flows associated with predicted changes in agricultural land use. There are a range of models available to estimate emissions as a function of land use and site management, from the IPCC Tier 1 methods (45) to more detailed process-based models, such as DNDC (46), RothC (47), or DAYCENT (48). These models vary with regard to the data requirements, computational intensity, and time required for interpreting the output. Within this project we chose to use the Cool Farm Tool (CFT) (49) as a model of intermediate complexity which requires as inputs general activity data and site characteristics provided by the other components of this project. Data inputs and section linkages are described in more detail below.

4.2. Objectives

The aim is to calculate the GHG flows associated with agricultural land use change. GHG flows are calculated as a function of soil type, land-use, and assumed farm management data to enable spatial projections. Further details regarding the caveats relating to emissions excluded from this calculation are given in the discussion of methods.

4.3. Data

Many soil based emissions from agriculture depend on certain soil characteristics as well as management practices. (50) for example, incorporate such characteristics within an empirical model of soil based nitrous oxide (N_2O) and nitric oxide (NO) emissions. To populate such model, the following variables were obtained for the UK from the Harmonized World Soil Database (HWSD) (51): soil texture, soil organic matter (SOM), soil moisture, soil drainage, soil pH bulk density, and direct and indirect N_2O emissions were estimated accordingly. The inputs are presented in more detail in Table 4.1.

Table 4.1: Categories for soil parameters as used in CFT.

Soil parameter	Classes
Soil texture	(i) coarse (sand, loamy sand, sandy loam, loam, silt loam, silt)<br>(ii) medium (sandy clay loam, clay loam, silty clay loam)<br>(iii) fine (sandy clay, silty clay, clay)
SOM	SOM <= 1.72<br>1.72 <= SOM <= 5.16<br>5.16 <= SOM <= 10.32<br>SOM >=10.32
Soil moisture	moist<br>dry
soil drainage	poorly drained - for fine soils<br>good drained - for medium and coarse soils
soil pH	pH <= 5.5<br>5.5 <= pH <= 7.3<br>7.3 <= pH <= 8.5<br>pH >= 8.5
Bulk density	values from Harmonised World Soil Database (source below)

Notes: Soil parameters from the Harmonized World Soil Database (HWSD) were categorized for soil texture, soil organic matter (SOM), soil moisture, soil drainage, soil pH and bulk density to give background information for calculating GHG
Source: (51).

Predicted land use information was obtained from the agricultural model describing the seven land use categories for farmland as follows: oilseed rape; cereals; root crops; grassland with rough grazing; permanent grazing; temperate grazing and other.

4.4. Methodology

Agriculture is a substantial emitter of GHGs through, for example, machinery use, mineral and organic fertiliser use, ruminant livestock, effects of both biomass and soil carbon stocks. Major carbon pools on land persist in living biomass (forests, perennials and tree-cropping systems), in addition to soil carbon.

Since most agricultural produce is for consumption within a period of months to a few years it is common practice not to account for photosynthetically fixed carbon in plant biomass or agricultural produce (52). The soil organic carbon (SOC) pool can be a substantial source or sink for emissions, although, except in the case of organic soils, the SOC pool tends to equilibrate under fairly constant land use (53). As a consequence, the major sources of emissions not related to energy use are nitrous oxide (N_2O) and methane (CH_4). N_2O emissions arise due to the mineralisation of nitrogen in organic matter (in the soil or for example in animal manures), and through the use of synthetic nitrogen fertilisers. Major sources of methane are from ruminant livestock (a function of dry matter intake) and manure management. Since dry matter intake is roughly proportional to animal size the key variables affecting GHG emissions are nitrogen fertiliser for field crops (54) and number of head for a given livestock species. These were thus the critical input variables required for the GHG modelling component.

Agricultural land is classified into seven categories. For each category a representative management regime is identified with specific fertiliser rates and machinery use characteristic of the UK. GHG emissions associated with livestock were incorporated into the analysis implementing the emission factors reported by (45).

4.4.1 Cool Farm Tool (CFT)

The Cool Farm Tool was employed to calculate GHG flows from agricultural land. This tool was originally developed for farmers to estimate the carbon footprint of crop and livestock products. It was designed to be both simple enough for general agricultural use, but scientifically robust for calculating carbon emissions. The CFT has been tested and adopted by a range of multinational companies who are using it to work with their suppliers to measure, manage and reduce GHG emissions in an effort to mitigate global climate change. The calculation of emissions is done at farm-level, based on land use and related information, and takes all relevant data on production processes, fertiliser use, energy and transport into account. The tool identifies hotspots and makes it easy for farmers to test alternative management scenarios, revealing those that will have a positive impact on net GHG emissions.

Methodologically the CFT sits between calculators using simple emission factor approaches (45, Tier 1) and Process-Based models that require a greater level of data input and training to interpret (45, Tier 3). The tool is divided into seven input sections as follows:

- General Information (location, year, product, production area, climate);
- Crop Management (agricultural operations, crop protection, fertilizer use, residue management);
- Sequestration (land use and management, above ground biomass);
- Livestock (feed choices, enteric fermentation, N excretion, manure management);
- Field Energy Use (irrigation, farm machinery, etc.,);
- Primary Processing (factory, storage, etc.,);
- Transport (road, rail, air, ship).

CFT (49) has been engineered in Microsoft Excel and is currently being adapted for online use.

The CFT employs a multivariate empirical model of (50) to estimate NO and N_2O emissions from fertiliser applications. The model is given as follows.

Equation 4.1:

$$\mathrm{N_2O} = e^{\mathrm{const}} + \sum_{1}^{n=i} \mathrm{Factor\ class}\ (i)$$

Where factor classes are: i) fertiliser type x fertiliser application rate; ii) crop type; iii) soil texture; iv) soil organic carbon; v) soil drainage, vi) soil pH; vii) soil carbon exchange capacity; viii) climate type; and ix) application method. Factors were determined by statistical analysis and are given in (50). The model for ammonia (NH_3) emissions differs marginally as follows.

Equation 4.2:

$$\mathrm{NH_3} = \mathrm{FA} \cdot e \sum_{1}^{n=i} \mathrm{Factor\ class}\ (i)$$

where FA is the amount of fertiliser applied. The model is described in (55).

Emissions from the production of nitrogen fertilisers are generally comparable in magnitude to field N_2O emissions. These emissions are often attributed to industry, although since they are produced for agricultural use it is often considered appropriate to incorporate these emissions in agricultural assessments and product carbon foot printing. Embedded emissions in other agro-chemicals are incorporated on a unit active ingredient using figures derived from the (56) harmonisation life cycle assessment.

Other embedded emissions (for example in machinery manufacture) are not included. Although this is somewhat inconsistent from a scoping point of view, there is no consensus on how to incorporate these emissions although they are acknowledged to be insignificant relative to other agricultural emissions sources (57).

For the present analysis we use the first two of the seven inputs for the CFT described earlier; the "General Information", and "Crop Management," programmed into MATLAB (2013) to calculate carbon emissions from agriculture for the UK. Therefore, representative management regimes include fertilizer use and emissions for machinery in six of the seven land use categories as shown in Table 4.2. For the remaining "other" land use category (as defined in the Defra June Agricultural Census (JAC); www.edina.ac.uk) we assume the following approximate breakdown into other land use classes: (i) cereals - 10%, (ii) horticulture - 20%, (iii) other agriculture - 45%, and (iv) woodland - 25%. Woodland GHG are considered

subsequently. For the horticulture subclass we assumed management as for root crops. For the “other agriculture” subclass we assumed 15% of the 45% to be fallow (no net emissions). For the remaining 30% of this subclass we assumed emissions to be an average of those from all other main land use classes.

For land management practices (Table 4.2) fertiliser use and general management of agricultural land were considered as typically used in the UK (St. Clair et al., 2008; Haverkort and Hillier, 2011; DEFRA 2011a). Fertiliser applications were estimated from DEFRA (2011a) and were weighted for the typical crops used in the UK.

Table 4.2: Management practices including fertilizer use for different land uses.

Land use	Fertiliser	Fertiliser (organic)	Management
Oilseed rape	N = 191 kg ha^{-1}<br>P_2O_5 = 58 kg ha^{-1}<br>K_2O = 65 kg ha^{-1}<br>CaO = 4400 kg ha^{-1}	N = 172 kg ha^{-1}<br>P_2O_5 = 52 kg ha^{-1}<br>K_2O = 58.5 kg ha^{-1}<br>CaO = 3960 kg ha^{-1}	Ploughing<br>Discing<br>Fertiliser spraying<br>Harvesting
Cereals	N = 146 kg ha^{-1}<br>P_2O_5 = 54 kg ha^{-1}<br>K_2O = 64 kg ha^{-1}<br>CaO = 4000 kg ha^{-1}	N = 131 kg ha^{-1}<br>P_2O_5 = 48.6 kg ha^{-1}<br>K_2O = 57.6 kg ha^{-1}<br>CaO = 3600 kg ha^{-1}	Ploughing<br>Harrowing<br>Gain drilling<br>Roller harrowing<br>Fertiliser spraying<br>Harvesting<br>Baling
Root crops	N = 129 kg ha^{-1}<br>P_2O_5 = 95 kg ha^{-1}<br>K_2O = 165 kg ha^{-1}	N = 116 kg ha^{-1}<br>P_2O_5 = 85.5 kg ha^{-1}<br>K_2O = 148.5 kg ha^{-1}	Ploughing<br>Field<br>Cultivating/ridging<br>Rotary hoeing/bed<br>Tilling<br>Planting<br>Tine harrowing/seed handling & transport<br>Fertiliser spraying<br>Potato harvesting
Grassland with grazing	-	-	-
Permanent grazing	N = 85 kg ha^{-1}<br>P_2O_5 = 21 kg ha^{-1}<br>K_2O = 25 kg ha^{-1}<br>CaO = 4300 kg ha^{-1}	N = 76.5 kg ha^{-1}<br>P_2O_5 = 18.9 kg ha^{-1}<br>K_2O = 22.5 kg ha^{-1}<br>CaO = 3870 kg ha^{-1}	Ploughing<br>Fertiliser Spraying<br>Harvesting
Temporary grazing	N = 118 kg ha^{-1}<br>P_2O_5 = 27 kg ha^{-1}<br>K_2O = 41 kg ha^{-1}<br>CaO = 4600 kg ha^{-1}	N = 106 kg ha^{-1}<br>P_2O_5 = 24 kg ha^{-1}<br>K_2O = 36.9 kg ha^{-1}<br>CaO = 4140 kg ha^{-1}	Ploughing<br>Fertiliser Spraying<br>Harvesting

Notes: Depicts typical land management practices for fertiliser use and general management of agricultural land as found in the UK. Fertilizer amounts are given for conventional practices for a mix of 95% conventional, 5 % organic.

Source: Derived from St. Clair et al. (2008), Haverkort and Hillier (2011), Jones and Crane (2009) and DEFRA (2011a).

Currently between 5 to 10% of the farms in the UK are considered to be organic Jones and Crane (2009). To reflect this in the study, we reduced all fertiliser use by 5% (Table 4.2) to accommodate a 5% minimum coverage as organic farms.

Emissions of CH_4 and N_2O from livestock (dairy and beef cows, and sheep) were estimated from (IPCC, 2006). The factors are summarised in Table 4.3. The calculation refers to a typical average weight of animals in the UK.

Table 4.3: Emission factors for CH_4 and N_2O from livestock.

Emissions	Dairy cows (600 kg)	Beef cows (300kg)	Sheep (65 kg)
CH_4 from fermentation<br>*(kg CH_4 head^{-1} yr^{-1})*	117	57	8
CH_4 from manure due to annual temperature (T=13°C)<br>*(kg CH_4 head^{-1} yr^{-1})*	27	8	0.19
N excretion rate<br>*(kg N (1000 kg animal mass)$^{-1}$ day^{-1})*	0.48	0.33	0.85
N_2O from manure<br>*(factor)*	0.02	0.02	0.01

Notes: Emissions factors per head for livestock
Source: IPCC (2006)

4.4.2. Validation and caveats

Using the management assumptions for the seven land uses, we obtain a total value for UK nitrate fertiliser use of around 1,331,286 t N/yr. It is noteworthy that the figure for nitrogen use somewhat exceeds the total synthetic N use figure from (55), which is approximately 1,000,000 t/yr., but both numbers are close. Possible reasons for overestimation of the total amount of fertiliser, include:

- Estimated fertiliser use does not consider organic farms. Calculations of fertiliser and emissions in the model with organic farms will be less;
- The classification of agricultural land into just seven land use categories required simplified assumptions regarding management, and an overestimation of fertiliser may have resulted;
- Most farms in the UK (70% -58) use a type of manure that reduces the general use of chemical fertilisers. In the current estimation we do not consider such uses.

The main reasons of uncertainties in estimations of direct and indirect N emissions from managed soils (and differences across studies) are related to the calculation of emission factors, the natural variability, partitioning fractions, lack of coverage of measurements and spatial aggregation (45).

Management of crop residues such as straw and other non-harvested crop biomass (e.g. burning the residue, composting it or leaving it on the farm) was not considered in the current study with the assumption that residue is exported from the site ("Export from farm"). Although management of such residual biomass can be a substantial source of emissions (e.g. 45) it is in practice very difficult to account for it. This is due both to a lack of data regarding common practice for its management and attribution or allocation between agricultural sectors.

We also did not include emissions due to the oxidation of organic (e.g. peat and fen) soils. Organic soils are typically water-saturated soils containing high densities of C, accumulated over many centuries. In order to use this land for agriculture, these soils need to be drained, which aerates the soil, favouring decomposition and therefore high fluxes of CO_2 and N_2O. The global warming potential (GWP) of N_2O over a 100 year time horizon is 298 (59) (i.e. effectively meaning that over a 100 year period 1 molecule of N_2O has the same global warming effect as 298 molecules of CO_2). Taking this into consideration, the GHG emissions from the Norfolk and Lincolnshire fens, for example, are probably underestimated.

4.5. Results

Per hectare estimated emissions for each land use class for an example soil type are shown in Table 4.4. For grazing land there are substantial differences between rough grazing land and improved pasture – with the former being essentially unmanaged except by grazing animals and the latter often receiving substantial fertiliser treatments in addition to other management activities, such as mowing and seeding. It should be noted that this table does not include the emissions from the livestock themselves, as this is a function of stocking rate rather than area per se. Emissions from the animals themselves are treated later. Here rough grazing is assumed - with no fertiliser or pesticides – which results in low emissions from the site (excluding livestock). Agrochemical use is highest for root crops. The “field energy use” reflects the machines used in the process, based on the assumption that diesel fuel is burned.

Table 4.4: GHG emissions in $CO_2e\ ha^{-1}\ yr^{-1}$ for different land use and management regime for single soil type.

		All data in kg $CO_2e\ ha^{-1}\ yr^{-1}$				
Land use	Fertiliser production	Background direct and indirect N_2O	Fertiliser induced field emissions	Agro-chemicals	Field energy use	Totals
Oilseed rape	1451 (1306)	164.2 (164.2)	669 (581)	102.5 (102.5)	113.2	2450 (2267)
Cereals	1248 (1123)	164.2 (164.2)	471 (413)	41 (41)	152.1	2076 (1893)
Root crops	531 (478)	164.2 (164.2)	404 (356)	164 (164)	130.4	1394 (1293)
Grassland with grazing		49.3				49.3
Permanent grazing	1090 (981)	48.1 (48.1)	167 (150)	123 (123)	44.4	1473 (1347)
Temporary grazing	1253 (1127)	48.1 (48.1)	238 (212)	123 (123)	44.4	1707 (1555)

Notes: Example of GHG emissions of $CO_2e\ ha^{-1}\ yr^{-1}$ for the stated land use and the following soil type: soil texture: medium, SOM: 1.72 – 5.16, soil moisture: moist, soil drainage: good, soil pH: 5.5 – 7.3. Emissions in parentheses are for a 95% conventional 5% organic mix.

4.5.1. GHG emissions in $CO_2e\ ha^{-1}\ yr^{-1}$

The GHG emissions per hectare vary as a function of farming system. Lowest values are for rough grassland – predominantly in the Scottish Highlands – on which agricultural production is limited and of relatively low intensity. Those areas in which the bulk of our cereal and field crops are grown have GHG emissions of the order or 1000-2500 kg CO_2e ha^{-1} yr^{-1} in which cases GHG emissions are mostly a function of nitrogen fertiliser use. However, it is worth stating that nitrogen use is generally controlled in the UK, and in good practice nitrogen is efficiently used so that inputs are matched to plant uptake.

Applying our definition of the "other" land use class discussed previously allows us to estimate per hectare emissions as: (i) 10% cereals; emissions = 208 kg CO_2e ha^{-1} yr^{-1} (ii) 20% horticulture/root crops; emissions = 279 kg CO_2e ha^{-1} yr^{-1} (iii) 30% of averaged emissions of the other 6 land uses and 15% bare soil with no emissions; emissions = 460 kg CO_2e ha^{-1} yr^{-1} and (iv) 25% wood; emissions = 0 kg CO_2e ha^{-1} yr^{-1}. So, as a result, the total estimated GHG emissions for "other" land use are estimated to be 947 kg CO_2e ha^{-1} yr^{-1}.

By considering 5% of all farms to be organic, the results show a clear reduction in the GHG emissions (Table 4.4) compared to the high fertiliser input for non-organic, intensive grazing grassland. Emissions from livestock are considered separately from the other land management emissions. Average emissions for dairy cows, beef and sheep were obtained by multiplying the (per head) emission factors below by the number of animals in each class within each grid cell. Based on the emission factors (Table 4.3) from (45) we calculated the following general GHG emissions for an annual mean temperature of 13°C:

- Dairy cow (600 kg) = 4585 kg CO_2e/head/ yr.;
- Beef cow (300 kg) = 1963 kg CO_2 e/head/ yr.;
- Sheep (65 kg) = 299 kg CO_2 e/head/ yr.

Model simulations were performed to examine the plausibility of estimates obtained from the analysis. Simulations for crops and grass reflected agricultural land use, yielding estimates of high GHG emissions for regions with intensive cropping or for grasslands with high stocking densities. In the north of the UK (Scotland) and in the west (Wales) rough grazing is the dominant land use with low emissions from soil and plants. The highest GHG emissions for crops go up to 2750 kg CO_2e ha^{-1} yr^{-1}. The GHG emissions from livestock show a different picture with highest emissions in intensive grazed regions (Wales, most of Scotland and north western England). Together, these result in total emissions up to 7700 kg CO_2e ha^{-1} yr^{-1} for intensively grazed regions. The lowest emissions are shown in east Scotland, for unmanaged grassland with a very low grazing intensity.

In general, higher emissions of GHGs results from regions in which there is substantial livestock production. The higher values of emissions (around 7700 kg CO_2e ha^{-1} yr^{-1}) result from areas of permanent grassland (which we have assumed to be improved and thus receive significant fertiliser inputs), where there is intensive dairy, beef, or sheep production. This is often in southern and western parts of GB on lands which are not generally suitable for cereal production. The assumptions regarding input use may influence the magnitude of the emissions from these areas. However, the general effect is robust given the outputs of the land use model, since ruminant livestock are known to be significant sources of GHGs from farming.

The current level of total emissions were calculated to be 51 Mt CO_2e per annum for crop land and livestock in England, Wales and Scotland. (60) calculated 49 Mt CO_2e for the agricultural sector in the UK in 2009. The close agreement of these numbers is felt to be acceptable given

slight differences in the scope of these calculations (our number includes around 5% for energy use and machinery but does not include Northern Ireland).

4.6. Discussion and Conclusion

The MATLAB coding used by the CFT calculates GHG emissions for the seven land uses by assuming corresponding typical management systems. These are generalisations to provide estimates of GHG emissions transferable across the entire country.

Livestock are a major contributor to total GHG emissions, and in particular, the total emissions are highly sensitive to stocking rates particularly for cattle and sheep, which are an important source of CH_4 from both enteric fermentation and from manure, and GWP of CH_4 is 25 times that of CO_2 (59).

The inclusion of organic farms reduces GHG emissions due to reduced fertiliser use. Less fertiliser use means lower GHG emissions, which is good in terms of GHG emissions mitigation. But with reduced fertiliser there is often a trade-off in the yield, which has consequences for food production, and may create a driver for land use change if any loss in production is to be compensated for by exploiting lands currently not under agricultural use. There is still a lot of research needed to find the ideal environmental optimum N rate by crop and region to compare with the current economic optima.

5: The forestry greenhouse gas module

5.1. Introduction

Analyses have shown that, even if emission reductions pledges are honoured in full (61), they will be insufficient to attain net zero and that GHG removal from the atmosphere will also be required (62). Of the options available, land use change is seen as essential (63; 64; 65) with afforestation identified as the GHG removal method which combines the highest CO_2 removal potential with lowest per tonne costs and greatest technology readiness level (65); see Annex A5). Assessments have identified that (contingent on emissions reductions being put in place) a 2050 target of 13 $MtCO_2$ pa of removals via new afforestation is consistent with attaining net zero (65).

This section describes research that estimates the annual greenhouse gas (GHG) flows arising from woodland and the afforestation of land, accounting for the emissions and sequestration associated with standing trees, deadwood and forest litter, soil and roots, and harvested wood products (HWP). These flows vary with the chosen forest management regime, which in our analysis entails a combination of 'felling' at the end of a rotation (the lifetime of a tree crop) and 'thinning' of a proportion of trees at various points within the rotation (typically undertaken to maximise overall timber revenues). This regime and the overall analysis allows direct compatibility with the guidelines provided in the UK Woodland Carbon Code (66).

All GHG measures are expressed as tonnes of CO_2 equivalent (tCO_2e) and are directly comparable with Woodland Carbon Units (*ibid.*). Our analysis is underpinned by the Forest Research CARBINE model (32), which is employed in a wide range of forest decision applications (67). However, its use here is confined to the estimation of GHG flows.

5.2. Objective

The objective of this module was to estimate the effect of forest planting on net annual carbon flows in livewood stands, deadwood and forest litter, soil and roots, and harvested wood products (HWP), for representative conifer (Sitka spruce) and broadleaf (Pedunculate oak) species.

5.3. Data

To predict the flow of GHG emissions from forestry activities, we relied on the CARBINE model, which uses inputs regarding tree growth rates derived from the Ecological Site Classification (ESC) decision support system originally developed by (26). Drawing upon the yield tables provided by (31), the updated ESC model (25) provides, at the 2km grid cell resolution, site-specific estimates of the maximum mean annual increment in timber volume by yield class (YC; measured in m^3/ha/yr.) for new plantations across the entirety of Great Britain, taking into account the local characteristics of planting sites.

These estimates provide the basic input to the CARBINE analysis of GHG sequestration and emissions associated with livewood, deadwood (including litter), soil carbon and harvested wood products (HWP).

For newly created forest areas, initial data inputs into CARBINE include: tree species, year of planting and age, in addition to management regime and rotation period (in years assuming a clearfell regime). In our analysis we adopt 2013 as the initial year of planting and apply the following assumptions:

- No fertilization or irrigation. This is a common management regime in UK forests.
- No genetic or agronomic improvements. Studies have found evidence of small yet significant increases in yield class over time (e.g. 68) which may indicate genetic or agronomic improvements. While we control for temperature and rainfall variation in our modelling, it is also possible that increases may be due to other climate related effects such as increases on CO_2 fertilisation (69; 70) although evidence of such effects to date is mixed, being mainly based upon experimental trials (71) rather than field studies (72). The assumption therefore would either have little effect or lead to a slight underestimation of growth rates.
- No pests or disease impact. The number of tree pest and disease incidents has increased markedly over the past half century (73) with devastating effects for certain species. The drivers of these incidents appear to be both due to climate change altering the habitable area for pests and pathogens and global trade increasing the biosecurity risks. the lack of definitive understanding of long term risk trends means that this factor is not considered within our analysis as any assumption is difficult to defend and potentially major in terms of its impact on estimates. However, we see this as a major area for research and note that our estimates should be viewed as a baseline from which pests and pathogens may generate substantial change.

In this analysis we focus on a single management regime: ‘thinning and felling’. Thinning involves the removal of wood at prescribed stages during the lifecycle of the stand. Thinning is assumed to start several years after planting (varying across species and YC) and then occurs at regular periods (e.g. every 5 years). Felling ages similarly vary by species and growth rates. The present analysis assumes species representative stands and tree density (on planting or regeneration) of 2,500 trees per hectare.

The spatially explicit nature of the analysis allows the calculation of species-specific carbon sequestration in livewood. Stem volumes (in units of cubic metres over bark per hectare) for both ‘standing’ and ‘removed’ wood are assessed for the chosen management regime. Stem biomass estimates are obtained by multiplying the species-specific stem volume (from 31) by a species specific value of wood density, expressed as oven dry tonnes of mass per cubic metre of ‘green’ timber volume (34). In the case of SS and POK these values are 0.33 odt/m^3 and 0.56 odt/m^3 respectively as shown in Table 5.1.

Table 5.1: Tree species and growth rates determining carbon sequestration in the CARBINE model

Tree species	Yield Class* (m^3/ha/yr.)		Basic density† (odt/m^3)	Allometric coefficients‡	
	Lowest	Highest		fR	fB
Sitka spruce	6	24	0.33	0.45	0.35
Oak	2	8	0.56	0.50	0.80

Notes: * Yield Class measures tree growth rate, defined as the maximum average rate of cumulative volume production over a rotation (the average rate of production will vary with the specified rotation)

† Basic density is defined here in units of oven dry timber (odt) mass per ‘green’ cubic metre.
‡ The allometric coefficient fR is used to determine the quantity of root wood, whilst fB is used to determine the quantity of branch wood and foliage combined.

Source: CARBINE model (32)

The figures reported in Table 5.1 summarise the rate of growth (YC), density of wood and allometric coefficients determining carbon sequestration for the two species under consideration. Here fR and fB are species-specific coefficients assumed to be constant with respect to tree age, size and growth rate. The values of these constants are based on interpretation of summary estimates of root, branch, foliage and stem biomass using the Forestry Commission forest stand biomass model BSORT (74). Together these data relate the timber yield class of a given species to its corresponding level of carbon sequestration.

5.4. Methodology

CARBINE is an analytical model of carbon exchanges between the atmosphere, forest ecosystems (trees, deadwood, litter and soil) and the wider forestry sector as a result of tree growth, mortality and harvesting (32; 75, 76; 67). Carbon sequestered in harvested wood of merchantable quality is allocated to HWP using a dynamic assortment forecasting model that accounts for variation in product out-turn specific to tree species and size classification of stem wood at the time of harvest (77). Different emissions are also considered depending on the classes of wood products in terms of their service life and the consequent time profile of carbon emissions. Emission profiles are set so as to emit all stored carbon over the lifetime of the relevant HWP. Carbon not sequestered in HWP is treated as waste and conservatively assumed to rapidly emit all stored carbon.

CARBINE consists of various sub-models, each estimating different aspects of forest carbon flows by calculating the stock levels at different points in time. The sub-models used in this analysis are:

- GHG sequestered in and emitted from livewood;
- GHG sequestered in and emitted from deadwood (litter);
- GHG sequestered in and emitted from soil;
- GHG sequestered in and emitted from HWP.

Note that a further CARBINE sub-model analysing the GHG implications of substituting timber for fossil fuels is not incorporated within the present analysis (although obviously such substitution raises the potential for afforestation delivering further net reductions in GHG emissions).

The sub-models for livewood and deadwood each consist of four elements assessing stems, branches, foliage and roots. Total tree volume is converted to oven dry biomass using the values of wood density described in Table 5.1, assuming a carbon content of 0.5 tC per oven dry tonne of biomass (78). Although the carbon content of woody dry matter is assumed to be constant, different tree species exhibit very different patterns of carbon sequestration. An overview of the structure of the CARBINE model, illustrating the processes and the various points of associated emissions, is provided in Figure 5.1.

Figure 5.1: Overview of the structure of the CARBINE model.

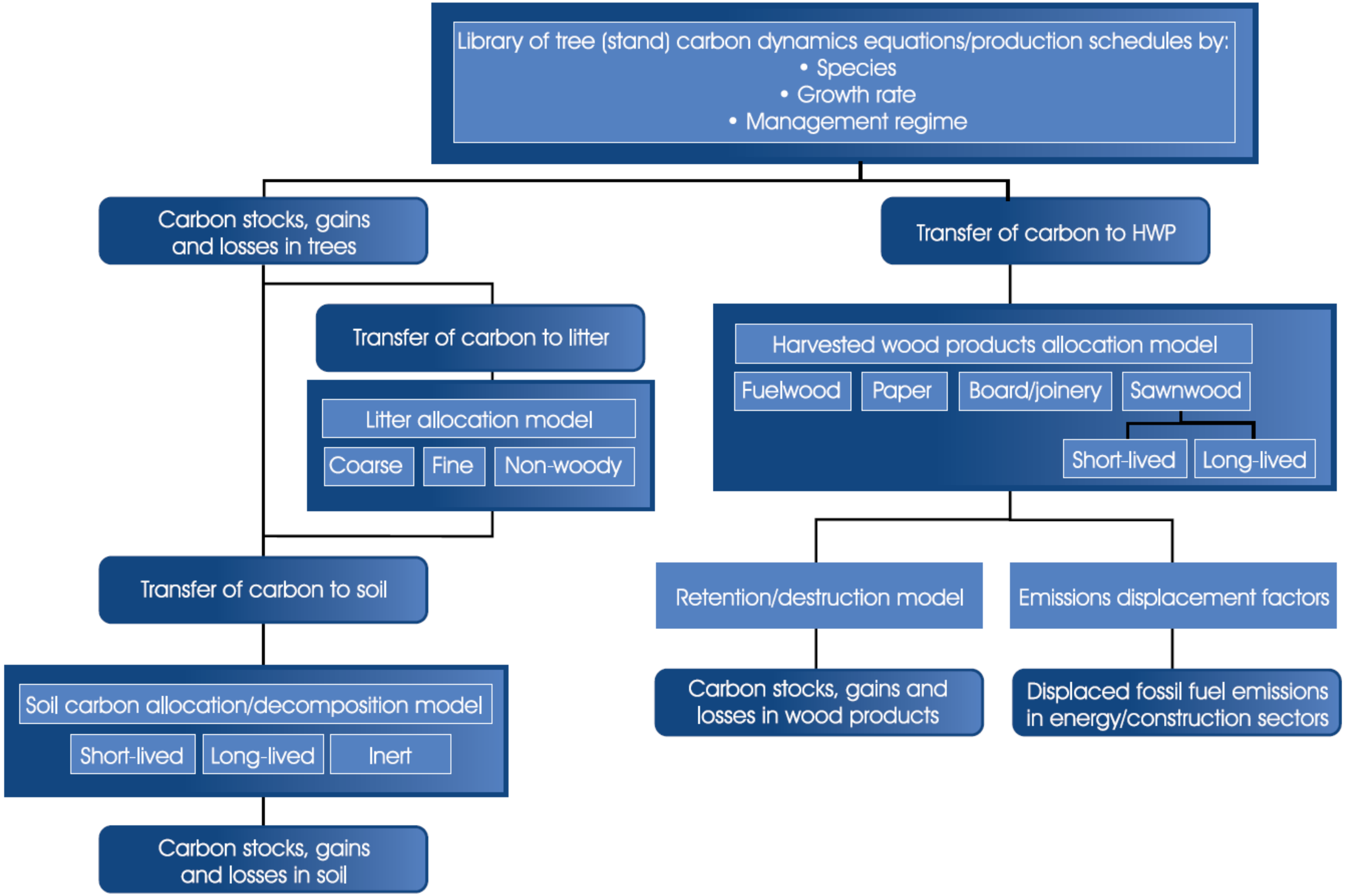

Source: 76, 67.

To obtain estimates of carbon and biomass in tree roots, branches and foliage the model relies on simple allometric relationships relating wood quantities to carbon sequestration, as defined by Equations 5.1 and 5.2 respectively.

Equation 5.1:

$$\text{Root carbon or biomass} = fR \times \text{Stem carbon or biomass}$$

Equation 5.2:

$$\text{Branch + foliage carbon or biomass} = fB \times \text{Stem carbon or biomass}$$

Figure 5.2 illustrates the CARBINE approach to allocating harvested wood between forest litter and primary products. Branchwood from harvested trees is assumed to be either used as wood fuel or left on site as part of the litter pool. The proportions allocated to be left on site or harvested for fuel are determined by simple partition coefficients, η_1 and η_2 (Figure 5.2). These coefficients are both set to 50% as per (76). The first step in the ultimate allocation of harvested stem wood to primary products involves an initial allocation to waste wood left as litter in the forest and to three ‘raw’ stem wood categories of ‘bark’, ‘small roundwood’ and ‘sawlogs’. The proportion of stem wood allocated to litter is determined by a partition coefficient, η_3, which is set to a standard value of 10% (33). The allocation of the remaining stem material to bark, small roundwood and sawlogs (otherwise known as a product assortment) is determined respectively by the partition coefficients η_4, η_5 and η_6, which depend on the size and shape of the harvested trees. In turn, tree size and shape depend on many factors but notably tree species, growth rate and the relevant management regime (79). The specific definitions used for small roundwood and sawlogs also influence these allocations.

Figure 5.2: The allocation of harvested wood inherent in the CARBINE model

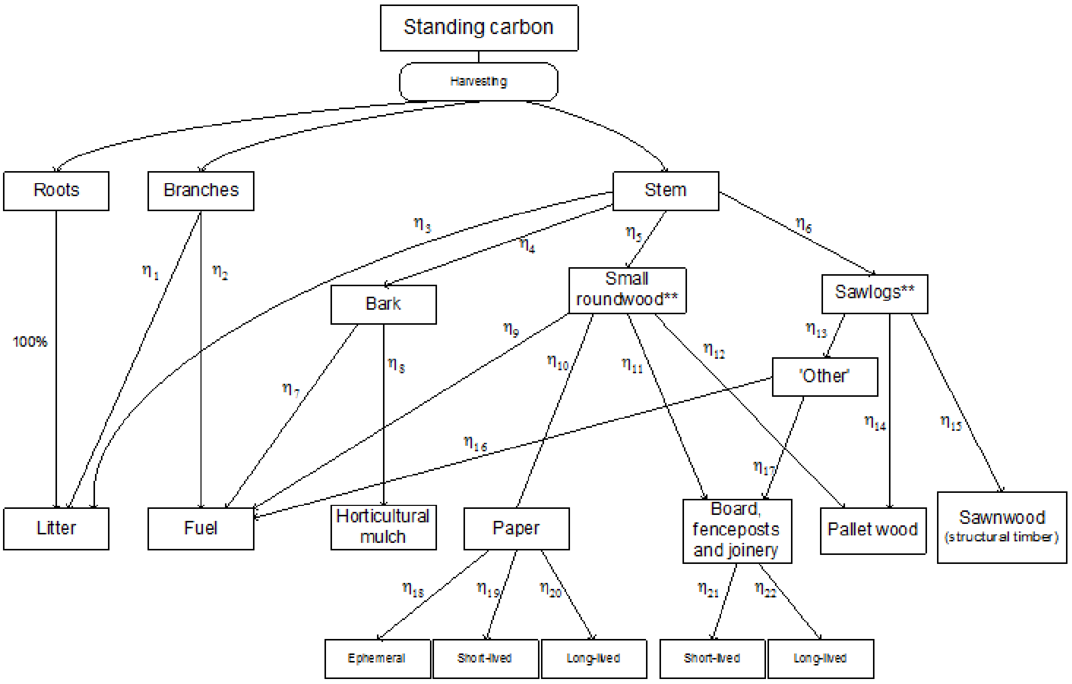

* Proportion varies with tree size and shape

** (not including bark)

Source: (76)

Assumptions regarding sawlog size were taken from previous applications of CARBINE (67) while the calculation of bark, small roundwood and sawlog partition coefficients (η_4, η_5 and η_6) were based on standard tables given in (79) and (31). However, some modelling of these results was necessary to enable the values in the tables to be accessed by variables available in CARBINE. The general form of the equations for estimating η_4, η_5 and η_6 expressed as percentages is given by Equations 5.3, 5.4 and 5.5.

Equation 5.3:

$$\eta_5 = 100 \times (1 - \eta_4 - \eta_6)$$

Equation 5.4:

$$\eta_4 = 100 \times (1 - fUB\ (dbh))$$

where fUB (dbh) is a function for estimating underbark stem wood volume (or biomass or carbon) as a fraction of overbark stem wood volume (or biomass or carbon) and dbh is taken as the quadratic mean of the diameter breast height of the harvested trees (79). The parameter η_6 is defined as:

Equation 5.5:

$$\eta_6 = 100 \times (fUB\ (species, dbh) \times fSAWLOG\ (dbh))$$

where fSAWLOG (dbh) is a function for estimating overbark sawlog volume (or biomass or carbon, for conifer or broadleaf sawlogs as defined above) as a fraction of overbark stem wood volume. Parameterization of fUB (dbh) and fSAWLOG (dbh) relies on piecewise relationships with respect to the quadratic mean dbh of harvested trees (for a fuller explanation see 79, and 31). These relationships also depend on tree species (or species group) and whether the stand has been thinned or not. The values assigned to other relevant partition coefficients are described in Table 5.2.

Table 5.2: Partition coefficients for allocation of 'raw' harvested wood material to primary wood product categories

Timber species group	Species-specific partition coefficients								
	Small roundwood				Sawlogs			'Other'	
	η_9	η_{10}	η_{11}	η_{12}	η_{13}	η_{14}	η_{15}	η_{16}	η_{17}
Spruces	20	20	35	25	70	0	30	43	57
Oak	80	20	0	0	80	15	5	56	44

Source: (79)

Finally, the soil carbon sub-model runs concurrently with the forest sub-model. Initial soil carbon is estimated based on land use/cover (e.g. arable, pasture, etc.) and soil texture (sand, loam, clay or peat). The structure and parameterisation of the soil carbon sub-model is based qualitatively on the Roth-C agricultural soil carbon model (47).

5.5. Results

In this section we summarise selected key results from the CARBINE analysis of the GHG impacts of afforestation. Figure 5.3 illustrates carbon sequestration (tCO_{2e}/ha) in livewood for an area planted with Pedunculate oak (POK) growing at YC4. The two lines shown illustrate the livewood storage occurring in each year and the cumulative storage for each rotation (with felling clearly shown where the cumulative curve returns to zero and the per annum (marginal) curve records a major negative value as stored carbon is transferred from livewood to HWP or waste forms). The shape of the cumulative storage graph indicates that maximum marginal storage is reached about two-thirds of the way through the rotation. The graph also underlines the long term nature of rotations for deciduous species, with felling arising some 150 years after planting in this instance. Figure 5.4 illustrates comparable curves for Sitka spruce (SS). While exhibiting similar marginal/cumulative relationships, SS rotations are typically much shorter (e.g. 58 years for YC14 SS).

Figure 5.3: Carbon (t CO_2/ha) in pedunculate oak (YC4) livewood per annum (marginal) and accumulated within a rotation, over three rotations.

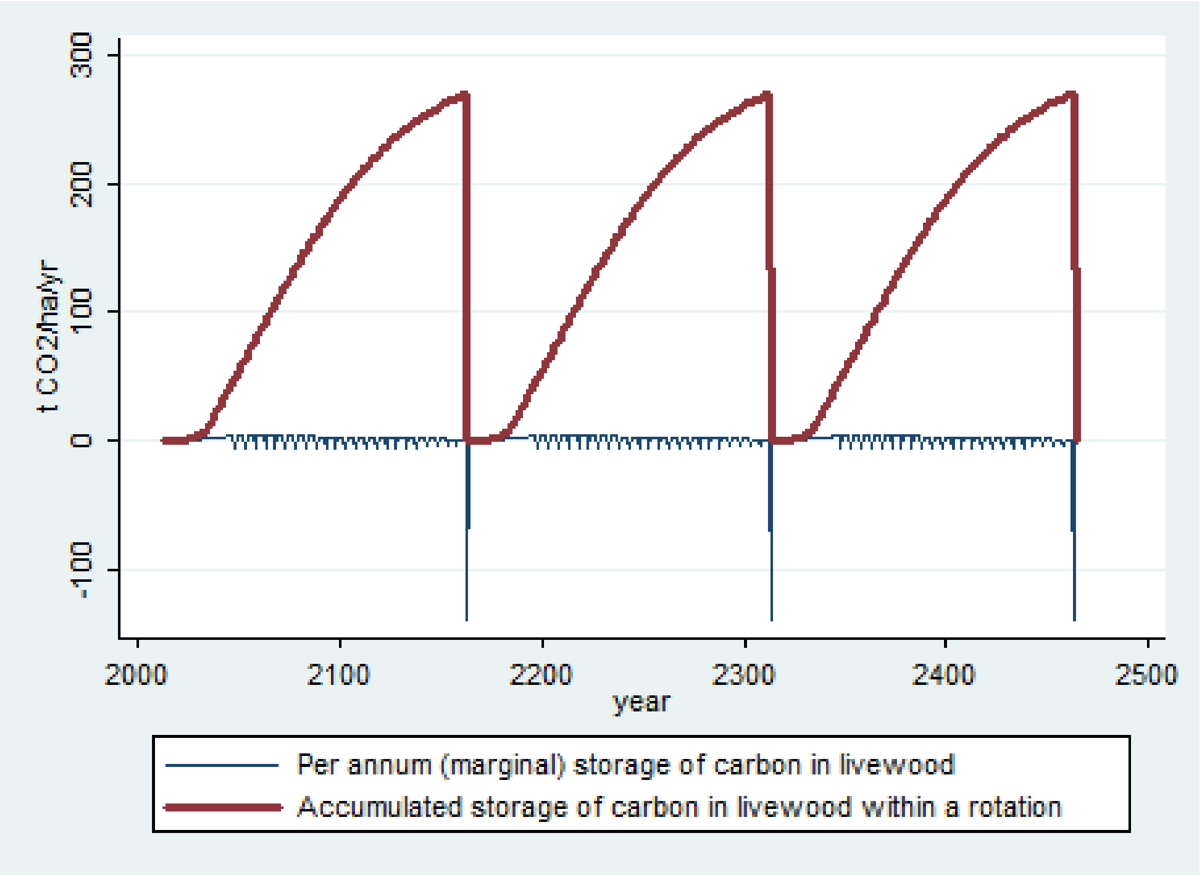

Source: Analysis using CARBINE (76)

Figure 5.4: Carbon (t CO_2/ha) in Sitka spruce (YC14) livewood per annum (marginal) and accumulated within a rotation, over five rotations.

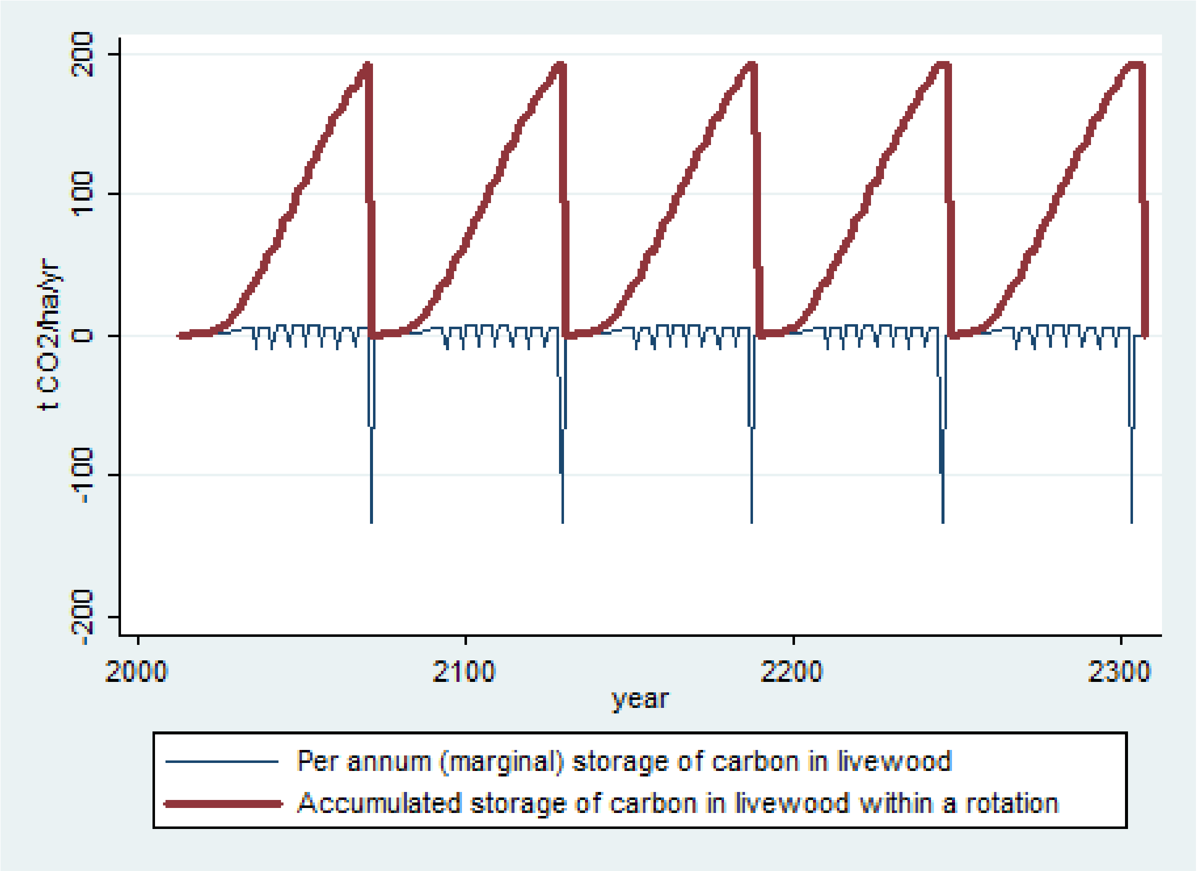

Source: Analysis using CARBINE (76)

Continuing with the POK example, Figure 5.5 graphs the marginal (per annum) and cumulative curves for the storage of carbon in HWP. This slowly increases over the first rotation and peaks immediately after felling. However, this peak is quickly reduced due to wastage and then more slowly erodes as we move further into the future as longer lived products slowly emit their stored carbon back into the atmosphere. This relationship is repeated for successive rotations. A somewhat similar pattern of build-up and then release is observed for carbon in forest litter.

Figure 5.5: Carbon (t CO_2/ha) in pedunculate oak (YC4) harvested wood products (HWP) per annum (marginal) and accumulated within a rotation, over three rotations

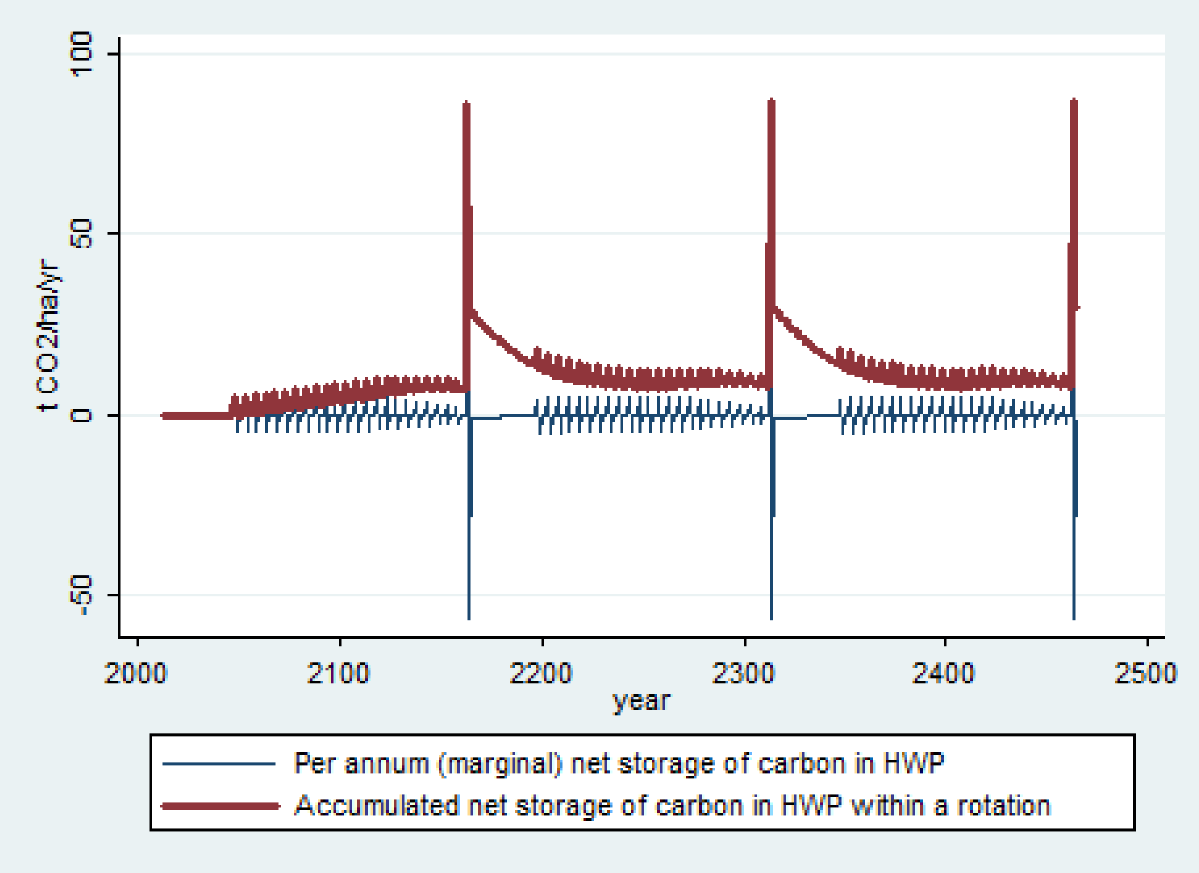

Source: Analysis using CARBINE (76)

Key to any forecasts of the soil carbon contribution to net GHG flow is the ability to take into account the land-use prior to afforestation. This is differentiated according to whether prior soil use was either classified as disturbed or undisturbed. Still considering POK, Figure 5.6 provides carbon profiles for both organic (peat) and clay soils, each being considered for both prior disturbed or undisturbed land use.

Figure 5.6: Carbon (t CO_2/ha) in pedunculate oak (YC4) accumulated or lost over three rotations for soil types: undisturbed clay; disturbed clay; disturbed organic; and undisturbed organic.

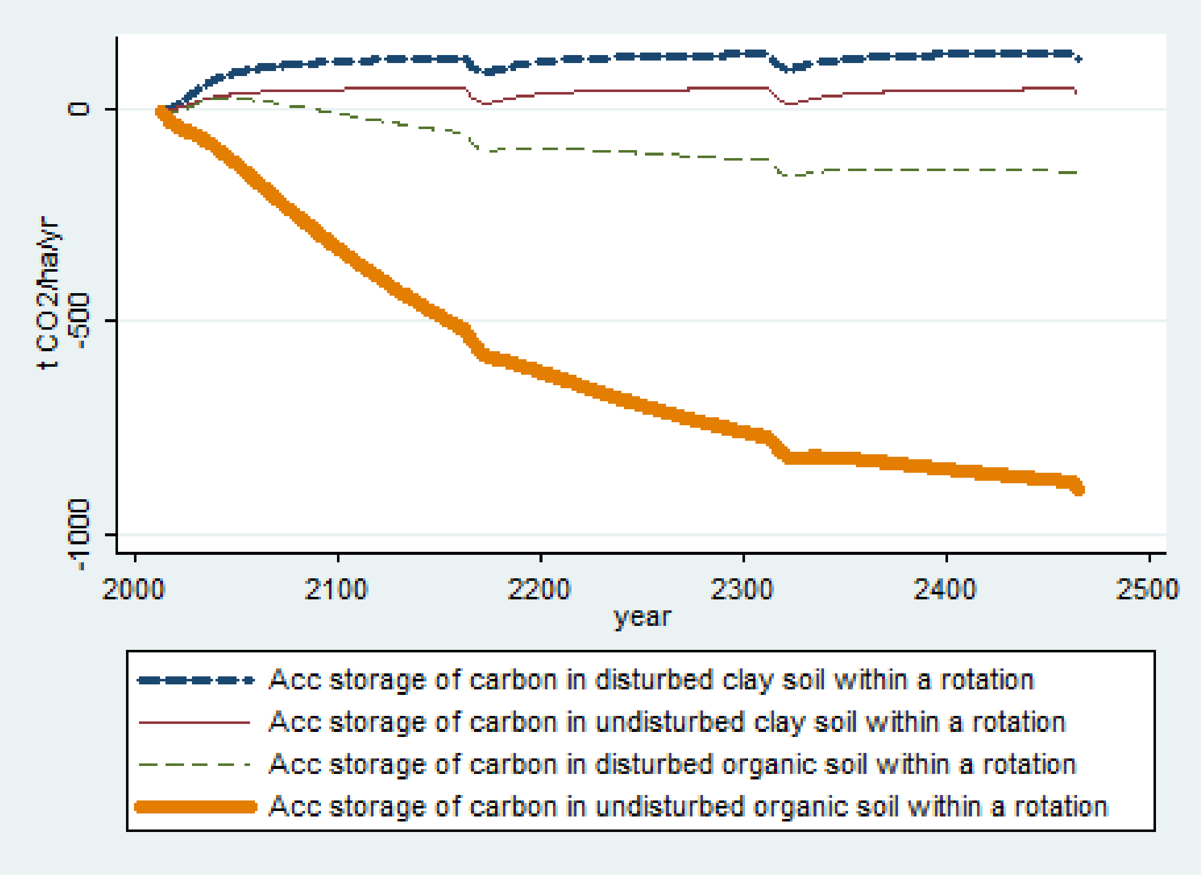

Source: Analysis using CARBINE (76)

The most striking feature of this graph is the strong reduction in soil carbon which occurs when trees are planted on previously undisturbed organic soils (e.g. peatland). Table 5.3 reports the quantity of carbon accumulated or lost for different soil types over one rotation of POK (similar patterns of soil carbon change occur for coniferous afforestation). The negative values for organic soils confirm, as in the Woodland Carbon Code guideline (66), that the woodland creation on organic soil cannot be an eligible activity, as it is associated to high quantity of carbon lost. This occurs because afforestation causes peats to dry out and release their previously stored carbon. Peatlands are in fact superb stores of carbon and their potential loss can be dramatic. In comparison, afforestation of previously disturbed peatlands results in a much smaller level of losses – although this merely reflects the fact that previous disturbance will have already lead to drying out and carbon release. In contrast, the afforestation of most other soils results in an increase in carbon storage. Here the change is greatest for previously disturbed soils (such as arable areas subject to regular ploughing) which are likely to have suffered prior depletion of their natural carbon stocks.

Table 5.3: Carbon (tCO_2/ha) in pedunculate oak (YC4) accumulated or lost over one rotation for soil types: undisturbed clay; disturbed clay; disturbed organic; and undisturbed organic.

Period	Disturbed mineral clay	Undisturbed mineral clay	Disturbed organic	Undisturbed organic
2013-2023	17.59	7.71	-1.58	-47.02
2024-2033	54.67	23.76	19.14	-69.14
2034-2043	77.37	33.40	26.44	-102.46
2044-2053	90.87	39.11	25.44	-141.70
2054-2063	99.26	42.58	20.16	-183.00
2064-2073	104.85	44.84	12.89	-224.18
2074-2083	108.87	46.44	4.79	-264.20
2084-2093	111.95	47.63	-3.53	-302.59
2094-2103	114.43	48.52	-11.79	-339.17
2104-2113	116.48	49.19	-19.85	-373.90
2114-2123	118.19	49.66	-27.67	-406.83
2124-2133	119.62	49.94	-35.21	-438.02
2134-2143	120.84	50.11	-42.44	-467.51
2144-2153	121.91	50.19	-49.32	-495.37
2154-2163	117.80	45.17	-60.92	-526.71
2164-2173	88.79	15.29	-96.99	-581.37

Source: Analysis using CARBINE (76)

5.6. Conclusion

The models described in this section are incorporated within our integrated modelling system permitting assessment of the consequences of afforestation upon the sequestration and emission of GHGs. This assessment is comprehensive in that it embraces GHG in livewood, waste and forest litter, products and soil carbon.

Annex A5: Cost-effectiveness, scalability and technology readiness of alternative approaches to greenhouse gas removal.

The Royal Society and Royal Academy of Engineering (65) provides a comparative assessment of the greenhouse gas removal potential of multiple technologies, most of them based upon land use change. These included: Afforestation, reforestation and forest management; Wetland, peatland and coastal habitat restoration; Soil carbon sequestration; Biochar; Bioenergy with carbon capture and storage (BECCS); Enhanced terrestrial weathering; Mineral carbonation; Direct air capture and carbon storage (DACCS) and; Building with biomass. Results are presented in Table A5.1 which is abstracted from that report.

As can be seen from the above table, the Royal Society and Royal Academy of Engineering assessment concluded that afforestation, reforestation and forest management provided the lowest cost per tonne of CO_2 removal. Cost-effectiveness is essential as, given the reality of fixed budgets for attaining net zero, reducing the costs per tonne of removal increases the level of greenhouse gas removal achieved. Furthermore, while forest management can remove 1-2 $GtCO_2$ pa, the potential removal afforded by afforestation and reforestation is up to ten times this level.

In summary therefore planting trees is the most cost-effectiveness, technology ready and scalable of all greenhouse gas removal options. Our focus upon tree planting as a means of greenhouse gas removal is therefore based upon sound, peer reviewed research.

Table A5.1: A summary of carbon dioxide removal potentials, costs and technology readiness levels (TRLs) from (65).

GGR method	Global CO_2 removal potential ($GtCO_2$ pa)	Cost per tCO_2 (US$)	Technology readiness level (TRL)
Increased biological uptake			
Afforestation, reforestation and forest management[234,235,236]	Afforestation/ reforestation 3 – 20<br>forest management 1 – 2	3 – 30	8 – 9
Wetland, peatland and coastal habitat restoration[237]	0.4 – 20	10 – 100	5 – 6
Soil carbon sequestration[238,239]	1 – 10	10 profit – 3 cost	8 – 9
Biochar[240,241,242]	2 – 5	0 – 200	3 – 6
Bioenergy with carbon capture and storage[243,244]	10	100 – 300	Bioenergy: 7 – 9<br>CCS: 4 – 7
Ocean fertilisation[245,246]	1 – 3	10 – 500	1 – 5
Building with biomass[247]	0.5 – 1	0	8 – 9
Natural inorganic reactions			
Enhanced terrestrial weathering[248,249]	0.5 – 4	50 – 500	1 – 5
Mineral carbonation[250]	–	50 – 300 (*ex situ*)<br>20 (*in situ*)	3 – 8
Ocean alkalinity[251,252]	40	70 - 200	2 – 4
Engineered removal			
Direct air capture[253,254,255]	0.5 – 5	200 – 600 (early stage)<br>100 (longer term)	4 – 7
Low-carbon concrete[256,257,258]	>0.1	50 – 300 (mineral carbonation)	6 – 7

There are 9 TRLs which describe the maturity of technology; TRL1 basic principles, TRL2 invention and research, TRL3 proof of concept, TRL4 bench scale research, TRL5 pilot scale, TRL6 large scale, TRL7 inactive commissioning, TRL8 active commissioning and TRL9 operations.

Note: These figures are typically developed with a range of different assumptions and predictions and so are not necessarily directly comparable. They represent technical potentials and may not take social aspects of deployment into consideration. Removal potentials demonstrate year on year removals, but for the GGR methods that saturate this does not continue indefinitely. Costs variably represent actual, predicted or targeted values. All are presented rounded to 1 significant figure to provide some guidance to order of magnitude expectations.

Source (65).

6: The recreation module - Impact of land use changes upon recreation values

6.1 Summary

A key consideration in the expansion of woodland planting in Great Britain is the possibility that new woodlands will provide increased opportunities for recreation. To estimate the magnitude of the benefits that might be realised by those increased recreational opportunities and to understand how those values might differ across planting locations requires the estimation of a recreational demand model. The structure of that model must be such that it allows estimation of the welfare benefits of new woodlands in monetary terms to allow direct comparison with the other costs and benefits of planting. Also, that model must capture fundamental characteristics of the welfare experienced by recreationists: particularly, that the benefits enjoyed from a recreational woodland decline both with increasing distance to that woodland and also with the increasing availability of alternative outdoor recreational opportunities. In this section, we report on the building of a recreational demand model that fulfils those criteria, with further detail presented in (1).

6.2. Theory and Economic Modelling

Our approach to estimating a recreational demand model adopts the long-established random utility maximization framework first developed by (80). That framework characterises recreational decisions as discrete choices of particular sites to visit out of an array of sites available, each offering different opportunities for outdoor recreational activities. In essence, the modelling approach seeks to establish the value of the recreational opportunities offered by visited sites starting from the availability of data recording information about the particular sites chosen for visitation, the set of sites that they could have possibly visited, and the characteristics of both visited and alternative sites.

More formally, imagine a dataset that records the outdoor recreational choices of a sample of individuals, indexed $i = 1,2,\dots,N$, on a particular day. Each member of that sample enjoys a set of possible sites that they might visit, indexed as $j = 1,2,\dots,J_i$, and the data records which particular site is chosen for a visit. The choice as to which site to visit will depend on a number of factors, but two important considerations are the quality of the recreational experience offered by a site and the cost in time and money of visiting that site. In our model, called travel cost model, the quality of recreational experience offered by site j is determined by the vector of site characteristics $\boldsymbol{x}_j$ and the costs of making a trip to site j by the travel costs tc_{ij}.

To construct our model, we first need to posit a function which describes the utility an individual will enjoy if they decided to visit site j. In line with the vast majority of the literature in this field we choose the simple linear approximation;

Equation 6.1:

$$v_{ij} = \alpha_j + \boldsymbol{x}_j\boldsymbol{\beta} + \gamma\left(I_{i,t} - tc_{ij}\right) \qquad (j = 1, 2, \dots, J_i \text{ and } \forall i)$$

where, $I_{i,t}$ is individual i's per period income, α_j is a site-specific utility element, $\boldsymbol{\beta}$ is the vector of coefficients describing the marginal utilities of site qualities and γ_i is the marginal utility of income.

Alternatively, an individual may choose not to make an outdoor recreational trip. We give that "no trip" option the index $j = 0$, and specify the utility from that option as;

Equation 6.2:

$$v_{i0} = \alpha_0 \qquad (\forall i)$$

Since the scale on which utility is measured is not known, we can make any arbitrary decision as to what quantity represent zero. For the purposes of this analysis we set α_0, the utility of the "no trip" option to zero such that the utility of other options is measured in comparison to the utility provided by this baseline option.

Adopting the familiar random utility maximization framework, we develop our econometric specification from Equation 6.1 by constructing the conditional indirect utility function;

$$u_{ij} = v_{ij} + \varepsilon_{ij} \qquad (j = 0, 1, \ldots, J \; and \; \forall i)$$

where ε_{ij} is an econometric error term introduced to capture the divergence between our model of utility (v_{ij}) and the individual's experienced utility (u_{ij}). Following standard practice, the error terms are assumed to be distributed $IID \; EV(0,1)$; that is to say, as independent draws from a standard Type I Extreme Value distribution.

In making recreational trip decisions it is assumed that individuals choose from the set of options $j = 0, 1, \ldots, J_i$, selecting that option which gives them the highest utility. Accordingly, the probability of observing individual i choosing to visit site k can be written as;

Equation 6.3:

$$\begin{aligned} P_{ik} &= Prob\left[u_{ik} > u_{ij} \;\; \forall\, j \neq k \,\right] \\ &= Prob\left[v_{ik} + \varepsilon_{ik} > v_{ij} + \varepsilon_{ij} \;\; \forall\, j \neq k \,\right] \\ &= Prob\left[v_{ik} - v_{ij} \; > \varepsilon_{ij} - \varepsilon_{ik} \;\; \forall\, j \neq k \,\right] \end{aligned}$$

Given the distributional assumptions regarding the error terms, Equation 6.3 results in an econometric expression for the probability of observing a particular recreational choice that takes the familiar multinomial logit (MNL) form;

Equation 6.4:

$$P_{ik} = \frac{e^{v_{ik}}}{\sum_{j=0}^{J_i} e^{v_{ij}}} \qquad (\forall i, k)$$

Given data on the recreational choices of the N individuals, it follows from Equation 6.4 that the log of the likelihood of observing those choices is;

Equation 6.5:

$$ln\, L(\boldsymbol{\alpha}, \boldsymbol{\beta}, \gamma) = \sum_{i=1}^{N} \sum_{j=0}^{J_i} Y_{ij}\, ln\, P_{ik}$$

Where Y_{ij} is a dummy variable which takes the value 1 if individual i chose recreational option j, or zero otherwise, and $\boldsymbol{\alpha}$ is the vector of utility elements specific to the different recreation trip options containing elements α_j $(j = 0,1, \ldots, J_i)$. The parameters of the model can be estimated using maximum likelihood methods by optimising Equation 6.5 with respect to the parameters of the utility function $\boldsymbol{\alpha}, \boldsymbol{\beta}, \gamma$.

The MNL is perhaps the simplest of the large class of econometric models that might be used to model recreational choices in the random utility framework. The MNL is adopted for the purposes of this research for a number of reasons. First, the datasets constructed for the purposes of estimating a recreational choice model for NEA-FO are extremely large: they need to be to provide a representative analysis of outdoor recreation decisions for GB. The simplicity of the MNL likelihood function Equation 6.4 allows the maximum likelihood routines to return estimates in timescales of several hours rather than the several days that would be required for more complex specifications. It would not have been practical to estimate those more complex models within the timescales of this project. More importantly, the MNL provides an expression for the expected welfare values that are derived from access to a set of recreational sites that takes a particularly convenient form;

Equation 6.6:

$$E[W|J_i] = \frac{1}{\gamma} ln\left(\sum_{j=0}^{J_i} e^{v_{ij}}\right)$$

In simple terms, given the assumptions of the MNL model, Equation 6.6 describes the analyst's best estimate of the maximum welfare, in money terms, that a respondent will enjoy from the J_i recreational activities open to them on any one choice occasion. The purpose of the NEA-FO analysis is to understand how that welfare might be enhanced by the provision of new recreational opportunities in the form of open access woodlands. So, for example, imagine a new woodland were added to an individual's recreational choice set, then from Equation 6.6 the expected value of that new woodland to individual i would be;

Equation 6.7:

$$E[\Delta W] = \frac{1}{\gamma} ln\left(\sum_{j=0}^{J_i} e^{v_{ij}} + e^{v_{iJ+1}}\right) - \frac{1}{\gamma} ln\left(\sum_{j=0}^{J_i} e^{v_{ij}}\right)$$

Notice that the log form of Equation 6.7 implies that as the number and quality of recreational opportunities available to an individual increases (i.e. the size of $\sum_{j=0}^{J_i} e^{v_{ij}}$ goes up) the smaller the additional welfare benefits enjoyed from the addition of the new woodland. In other words, individuals well-endowed with recreational opportunities will value an additional woodland less than those with relatively few recreational opportunities.

Now, imagine that there existed M locations in which new woodlands might be planted and we faced the problem of choosing N $(N < M)$ locations in which to plant in order to maximise recreational welfare values. Any particular planting decision could be described by a vector $\boldsymbol{d}$, where $\boldsymbol{d}$ has M elements, one for each potential planting location, and in which the mth element, d_m, records a 1 if planting occurs at that site and a 0 otherwise. Clearly, the elements in $\boldsymbol{d}$ will sum to N. In this case, the welfare benefits of a particular planting decision for individual i will be given by;

Equation 6.8:

$$E[\Delta W] = \frac{1}{\gamma} ln\left(\sum_{j=0}^{J_i} e^{v_{ij}} + \sum_{m=1}^{M} d_m e^{v_{im}}\right) - \frac{1}{\gamma} ln\left(\sum_{j=0}^{J_i} e^{v_{ij}}\right)$$

It turns out that the expression in Equation 6.8 has a number of important features with respect to the argument $\sum_{m=1}^{M} d_m e^{v_{im}}$: that is to say, with respect to the element of Equation 6.8 that

reflects our planting decisions. First, it is monotonically increasing in that argument and second it evaluates to zero when that argument takes a value of zero. Those two features mean that Equation 6.8 can be linearised in a way that allows the use of relatively simple methods of integer programming to select the optimal set of planting locations. This is discussed in greater detail in (2).

Use of the MNL does, however, entail accepting some limitations to the realism of the model. In particular, the MNL does not allow for particularly realistic patterns of substitution between options, such as the fact that the certain elements of the choice set might be much closer substitutes than others. So for example, imagine two individuals, one with a choice set replete with woodland another with a choice set with very few opportunities for woodland recreation. For the sake of argument, however, assume that both individuals enjoy approximately identical welfare values from the recreational opportunities afforded by their different choice sets. Now imagine we were to extend both individuals' choice sets by adding additional woodland. Intuition informs us that that additional woodland would offer much greater welfare gains to the individual lacking in woodland recreational activities. Observe from Equation 6.7, however, that the MNL would prescribe that both individuals enjoy the same welfare gain from that addition to their choice set.

Models exist that might provide more detailed substitution patterns. One potential extension would be to estimate a Nested Multinomial Logit Model (NMNL) which can be specified to allow for groups of similar types of site to exhibit much closer substitution relationships. Unfortunately, moving to a NMNL specification would introduce complexity in using the recreational model in the identification of optimal planting strategies. Accordingly, that extension is the focus of future research endeavours.

6.3. Data

The estimation of a discrete choice recreational demand model requires the compilation of a dataset that details two key items of information:

- *Choices:* The recreational decisions made by a sample of households: that is to say, a dataset which describes whether a household chose to make an outdoor recreational trip on a particular occasion and, if they choose to make such a trip, where they decided to visit.

- *Choice Sets*: Details of the set of outdoor recreational sites that each of those households might potentially have chosen to visit: that is to say, households' recreational choice sets.

Constructing such a dataset for the purposes of the NEA-FO project presents two unique challenges. First, the NEA-FO project is pursued at the scale of a nation, the vast majority of previous recreational modelling exercises focus on a considerably smaller spatial scale. Second, most of those previous modelling exercises focus on one particular form of outdoor recreation; most frequently fishing trip or trips to beaches. The NEA-FO project requires a model which can distinguish the benefits that come from woodland recreational sites in the context of all alternative outdoor recreation opportunities. Accordingly, the development of a dataset for the NEA-FO recreational model has necessitated the creation of a recreational

choice dataset of unprecedented scope and detail and required the use of advanced software applications capable of processing and manipulating enormous datasets.

6.3.1. Outdoor Recreation Activity Data

At the core of the NEA-FO recreational choice dataset is data collected from Natural England's national survey entitled the *Monitor of Engagement with the Natural Environment* (81). Similar surveys are undertaken in Scotland and Wales, but the unique feature of MENE is that it records the exact destination of recreational trips taken by respondents. While it may be possible to extract useful information from the Scottish and Welsh surveys, since they do not record information on recreational destination the data they provide is not immediately amenable to recreational demand modelling.

In its present form, the MENE survey began in 2009-10 with surveys being undertaken each year through to 2012-13. In total, the MENE dataset records between 9,000 and 10,000 respondent interviews each year: a total of 37,571 observations. Each observation provides details of the outdoor recreational activities of a household member over the course of the last week. If the respondent has involved themselves in such activities, then one particular trip is chosen at random. The MENE data records information on the activities undertaken on that trip, the nature of the outdoor location visited and its approximate geographic location. The MENE dataset is provided with weights that allow analysts to derive nationally representative statistics from the data.

6.3.1.1. Outdoor Recreation Site Data

Perhaps the greatest challenge in constructing the NEA-FO recreation data set has been identifying a comprehensive, spatially-referenced catalogue of outdoor recreational sites in Great Britain. No such dataset currently exists. From the outset, we defined three qualitatively different forms of outdoor recreational site:

- *Area features (Parks)*: These recreational sites are prescribed by some well-defined boundary. Recreational activity is allowed across most, or all, of the site and the provision of recreational services is often the primary, or sole, purpose of the site. Good examples of area features include municipal parks, nature reserves and recreational woodlands. We use the generic term parks to refer to sites of this type.

- *Linear features (Paths)*: These recreational sites are prescribed by linear rights of way, usually in the form of footpaths or bridleways. Often used for walking or hiking, these rights of way may pass through agricultural land, along rivers or coastlines or over areas of semi-natural land. In their use of these linear features, recreational users will usually not deviate from the path into the surrounding countryside and indeed may not have the right to do so. We use the generic term path to refer to sites of this type

- *Beaches*: With characteristics of both linear and area features, we include beaches as a separate category for the purposes of our analysis.

Data on area features were compiled from an array of geographical information system (GIS) resources. Detailed information on those data sources is provided in (2). In brief, accessible

recreational woodlands in GB were identified from the Woodland Trust's *Woods for People* project (reported annually, e.g. 82) and the characteristics of those woodlands (primarily whether broad-leafed or coniferous) were determined by cross-referencing with the Forestry Commission's inventory of the UK's woodland estate. Data regarding the location of national and local nature reserves, as well as country parks, National Trust properties and doorstep and millennium greens were compiled from a variety of mainly government sources. The type of habitat characterising those recreational sites was determined through overlaying CEH's *Landcover* dataset (see Table 1.1), allowing sites to be categorised as primarily semi-natural grassland, wetlands or mountains, moors and heaths. Likewise, recreational sites were categorised as being lake or river sites if those features dominated the site. One major category of outdoor recreational site not represented in those datasets is that of municipal parks, recreation grounds and commons (often termed urban greenspace). Since, no GB dataset exists for such sites, their locations were determined from interrogation of the rich resource provided by the Open Street Map (83) project.

OSM was also instrumental in defining linear features. The GB network of public access paths and bridleways (from now on just paths) was extracted from OSM. Paths in urban areas or in recreational parks were extracted leaving just those that passed through natural areas and through agricultural land. Notice that many of the recreational opportunities afforded by the UK's national parks were captured by way of their paths network. A single 'path' recreational site was identified as a contiguous network of connected paths. The characteristics of each of those paths was established according to the type of habitat they passed through and by their proximity to rivers, lakes and coasts. Beaches were identified through reference to a variety of sources documented (2).

Table 6.1 documents the types and number of outdoor recreational sites identified in the construction of the recreational choice dataset.

Table 6.1: Recreational sites identified in the recreational choice dataset.

Site Type	Number of Sites	Number of Size Categories
Beach:		
Beach	505	1
Area Features:		
Municipal		
Parks	7,307	3 (≤25ha, >25ha&≤75ha, >75ha)
Recreation Grounds	5,031	3 (≤25ha, >25ha&≤75ha, >75ha)
Commons	1,399	3 (≤25ha, >25ha&≤75ha, >75ha)
Woods		
Broad Leaf	13,209	3 (≤50ha, >50ha&≤150ha, >150ha)
Coniferous	4,375	3 (≤50ha, >50ha&≤150ha, >150ha)
Rural		
Semi-Natural Grassland	1,042	2 (≤50ha, >50ha)
Wetland	118	1

Mountains, Moors & Heaths	228	1
Country Park	589	1
National Trust	125	1
Coastal	51	1
Water:		
Rivers	506	1
Lakes	144	1
Linear Features (Paths):		
Natural:		
Mountains, Moors & Heaths	1,024	2 (≤5km and >5km)
Woodland Broad Leaf	1,149	2 (≤5km and >5km)
Woodland Coniferous	499	2 (≤5km and >5km)
Farm and Grassland		
Farm	15,486	2 (≤5km and >5km)
Semi-Natural Grassland	2,066	2 (≤5km and >5km)
Water		
Coastal	523	2 (≤5km and >5km)
Estuary	203	2 (≤5km and >5km)
Rivers	1,469	2 (≤5km and >5km)
Lakes	186	2 (≤5km and >5km)
Total	57,224	43

6.3.1.2. Choice Sets

The recreational site dataset was used to identify a choice set for each respondent in the MENE survey data. That data identified the lower super output area (LSOA) of each respondent's home. Accordingly, the outset location for recreational trips for each respondent was taken as the population weighted centroid of the LSOA in which they reside.

Using a UK roads dataset provided by the OS, a GIS roads network was constructed for the UK in which the driving time along every stretch of road was established from average driving speeds on roads of different categories. Access points to recreational parks or paths were taken at points at which those paths are parks intersected the roads network (except where those intersections were on motorways or dual carriageways). Accordingly, large parks or extensive paths are often characterised by a large number of access points.

To create a choice set for each respondent, the 23 recreational site types described in Table 6.1 were further subdivided by size (see final column of Table 6.1) to generate 42 different categories of recreational site. For each respondent in the dataset GIS software was used to locate the 10 nearest sites of each type. Accordingly, each respondent's choice set was taken to comprise that array of 430 sites. Two additional options were added to each respondent's

choice set: the option to not take a trip ("no trip" option) and the option to take a trip to a site not present in their choice set ("other trip" option).

Finally, the GIS network software was used to record the travel time and travel distance to each of the sites in a respondent's choice set through the roads network. Travel times and distances were converted to travel costs by using an approximation to each respondent's cost of time calculated as a third of their after tax hourly income and adding on a cost of travel calculated as £0.25 per kilometre travelled.

Since, 883 respondents to the MENE survey indicated that they had not started their trip from their home, the travel costs calculated in this way would not be correct. Accordingly, those observations were excluded from the data. All the same, the final dataset used in the analysis contained information on almost 15 million respondent-site choice options.

6.4. Matching Choices to Sites

The next step in constructing the NEA-FO recreational dataset was to establish which particular site each respondent had chosen to visit for a recreational trip. Of the 37,571 observations in the MENE dataset, some 22,562 respondents had not taken an outdoor recreational trip over the course of the last seven days. Those individuals were catalogued as choosing the "no trip" option.

The MENE dataset failed to record the destination location for a further 2,258 respondents. As a crude approximation, those respondents were categorised as choosing the "other site" option. Given more time, those choices could be handled in a more realistic manner, particularly accounting for the fact that those respondents might actually have chosen one of the options in their choice sets.

A further set of observations were attributed to the "other trip" option on account of the information in the MENE dataset suggesting that they had not visited a site identified in the recreational site dataset. In particular, trips described as being to an "allotment" or to a "village" were handled in this manner.

For the remaining observations, the MENE dataset records answers to a series of questions that provide insights as to the nature of the recreational site the respondent visited. For example, the survey records whether the respondent went for a walk on a path or took part in an activity involving water, the data also records whether the respondent visited a wood or a beach or a municipal park. That array of information was used in identifying which particular recreational site the respondent had visited. As a first step, GIS software was used to identify the 50 closest path sites, 50 closest park sites and 50 closest beach sites to each respondent's destination location as recorded in the MENE data. Then a scoring system was devised in which the characteristics of each site were compared to the description of the visited site recorded in the MENE data. The closer the site matched the description provided in the data, the higher its score. Finally, each sites score was inverse-weighted by its distance from the MENE-recorded destination location. The chosen site was taken to be that site with highest distance-weighted score.

Where the distance-weighted score exceeded a threshold level, it was determined that the actual site visited had not been found in the recreation site dataset either because that site had not been

identified in compiling that dataset or because the destination location in the MENE dataset was incorrectly recorded.

Table 6.2 reports the breakdown of trips in the data set that were made to sites of different types. Notice how almost half of the trips taken by respondents in the data are to municipal parks and recreation grounds. Importantly, for the purposes of the analysis, trips to woodlands are also shown to be an important recreational destination.

Table 6.2: Proportion of respondents making an outdoor recreational trip visiting different types of site

Site Type	Percentage of Respondents
Beach:	7.99%
Area Features:	
Municipal	
Parks & Rec. Grounds	43.33%
Commons	2.88%
Woods	
Broad Leaf	15.32%
Coniferous	1.77%
Rural	
Wetland	0.50%
Mountains, Moors & Heaths	0.11%
Semi-Natural Grassland	2.12%
Country Park	7.58%
National Trust	0.79%
Water:	
Rivers & Lakes	1.87%
Linear Features (Paths):	
Natural:	
Mountains, Moors & Heaths	0.44%
Woodland	0.81%
Farm and SNG	
Farm and Grassland	11.68%
ln(length of path)	
Water	
Coastal	0.78%
River or Lake	2.01%

As a final step, the site identified as a respondent's chosen destination was searched for in each respondent's choice set. For the majority of observations, the visited site was identified. For those observations where the site was not found in a respondent's choice set, that respondent was characterised as choosing the "other trip" option. A better approach would have been to add the identified destination to the respondent's choice set, but for technical reasons and for lack of time that extra processing step was not undertaken.

6.5. Modelling Results

A multinomial logit model of the form described above was estimated using specially written code which employed a number of programming tricks in order to speed up execution with extremely large datasets. The model was specified using a relatively simple specification for the utility function (1). In particular, a constant was included for each type of recreational site and the value of the recreational benefits coming from sites of different types allowed to vary according to the natural log of the area (park sites) or length (path sites) of the site. The estimated parameters of the model are shown in Table 6.3.

Table 6.3: Parameter estimates from a multinomial logit model estimated on the recreational choice dataset

Parameters	Coefficient	Robust std. errors	t-stat	p-stat
Travel Cost	-0.3353	0.0049	-68.4377	<0.001
No Trip (Baseline)	0			
Other Sites	-1.3179	0.0149	-88.4484	<0.001
Beach:				
Beach	-5.3490	0.3345	-15.9909	<0.001
Area Features:				
Municipal				
Parks & Rec. Grounds	-4.1898	0.1330	-31.5024	<0.001
Commons	-4.5799	0.1500	-30.5327	<0.001
ln(area)	0.0676	0.0125	5.4091	<0.001
Woods				
Broad Leaf	-5.1707	0.1882	-27.4742	<0.001
Coniferous	-5.1605	0.2021	-25.5342	<0.001
ln(area)	0.1296	0.0172	7.5362	<0.001
Rural				
Wetland	-5.7419	0.3586	-16.0120	<0.001
Mountains, Moors & Heaths	-7.1961	0.4171	-17.2526	<0.001
Semi-Natural Grassland	-6.8321	0.2943	-23.2149	<0.001
Country Park	-6.0077	0.3266	-18.3948	<0.001
National Trust	-6.0448	0.3454	-17.5007	<0.001

ln(area)	0.2856	0.0239	11.9491	<0.001
Water:				
Rivers & Lakes	-4.7912	0.5657	-8.4695	<0.001
ln(area)	0.1992	0.0473	4.2114	<0.001
Linear Features (Paths):				
Natural:				
Mountains, Moors & Heaths	-6.2508	0.3221	-16.814	<0.001
Woodland	-7.4496	0.3053	-21.429	<0.001
ln(length of path)	0.4411	0.0317	13.295	<0.001
Farm and SNG				
Farm and Grassland	-6.8321	0.2489	-25.498	<0.001
ln(length of path)	0.4377	0.0278	15.145	<0.001
Water				
Coastal	-7.5609	0.6157	-11.096	<0.001
River or Lake	-8.3692	0.5994	-12.642	<0.001
ln(length of path)	0.5982	0.0679	8.704	<0.001

One item of immediate note from Table 6.3 is that each and every parameter in the dataset is significant at higher than the 0.1% level of confidence. Of course, such an outcome is not unexpected given the fact that the model is estimated on a dataset comprising 34,653 observations. Also, recall that the baseline level of utility in the model is taken to be that provided by the "no trip" option. Since a sizeable majority of respondents did not choose to take a trip to an outdoor recreational sites it is not altogether surprising that the model estimates that the utilities derived from trips to such sites are significantly lower than that offered by the "no trip" option.

Examination of the estimated parameters suggests a broad consistency with prior expectations. Utility is decreasing in travel costs, such that respondents' behaviour suggests a preference for nearer recreational sites over more distant ones. The utility of those recreational sites is significantly increasing with the log of size and this is true across the range of outdoor recreational sites examined in the model. Parks in general (that is to say, sites defined as a specified recreational area) tend to command greater utility values than paths, with municipal parks, commons and recreation grounds offering the highest recreational values of all sites. Woodland recreational sites tend to fall somewhere in the mid-range of recreational values with no significant differences between the recreational values ascribed to broad-leaf woodland as compared to coniferous woodland.

6.5.1. Predicting Recreational Welfare

The recreation model implicitly assumes that the effect of changes in land use over time can be approximated by differences across space. For the purposes of examining optimal planting decisions, the model parameters shown in Table 6.3 were used to predict recreational welfare values across GB. First, the distribution of population across GB was simplified by ascribing the population in each lower super output area (LSOA) to the 2km grid cell within which its

central point falls. That procedure resulted in the identification of just over 11,500 population locations. For each population location a choice set of 432 recreational options was constructed in exactly the same way as described for the recreational data set. Finally using the estimated parameters and those choice sets, Equation 6.6 was evaluated to establish the current levels of welfare being enjoyed at different population locations across the UK. The geographic distribution of those annual recreational welfare values per year is illustrated in Figure 6.1.

Figure 6.1: Annual welfare benefits from access to current set of outdoor recreation opportunities

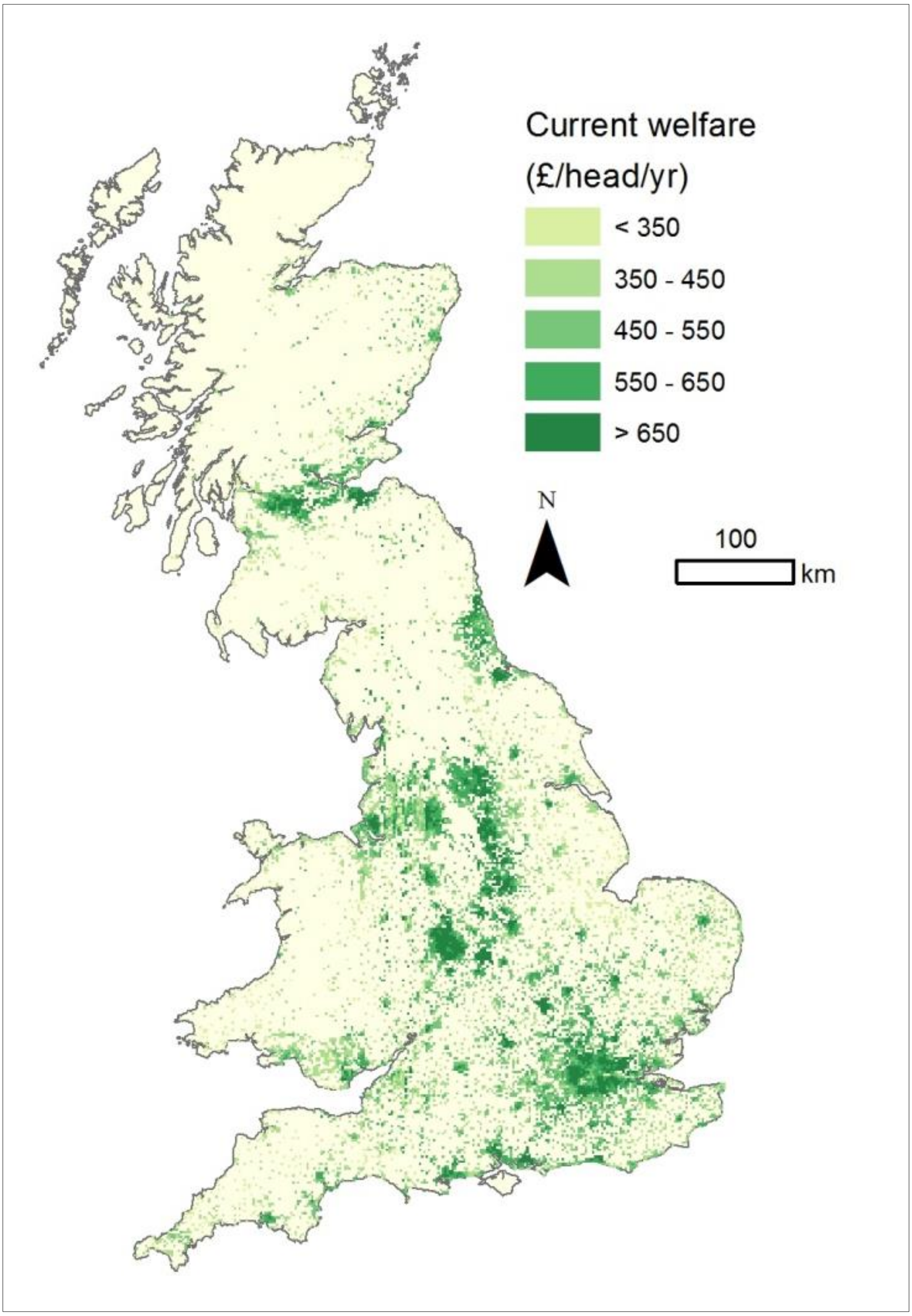

While a detailed discussion of the distribution of current welfare values is not the focus of this investigation, it is interesting to note that significant differences occur across GB with values ranging from a low of £258 to a maximum of £959 per person per year and that in part those difference reflect differences in the availability of recreational opportunities across the country.

To gain a better understanding of how the planting of new woodlands might impact on recreational welfare values a further investigative analysis was undertaken. In particular, using Equation 6.7 the welfare gains realised by individuals in each population location were estimated for two scenarios. The first scenario envisages a 100 ha broad-leaf woodland planted

10 minutes one-way drive time of that location. The second scenario considers the same sized wood but now planted 20 minutes' drive from the location. Results from these analyses are illustrated in Figure 6.2.

Consider first, the top row of Figure 6.2 which illustrates the welfare benefits from the first scenario of a 100 ha woodland planted at 10 minutes distance. Results are presented on the left-hand map as values per head. Here the annual welfare benefits of such a woodland would average £3.02 per head per year, but again considerable geographical variation exists in this value partly as a result of differences in the availability of recreational opportunities across GB. For example, the per head welfare gains appear to be relatively lower in London than they are in areas of north-west England or South Wales. That information on its own might suggest that the latter areas represent a preferred planting location to the former.

Now observe the right hand figure in the top row which shows values aggregated for everyone living within a population location. This map shows the same data but now multiplied up by the size of the population in that first, ten minute, scenario. The important thing to note here is that the weight of population in each location matters. Now the greatest gains in welfare are achieved by planting close to the heavily populated urban regions in, for example, London and Birmingham. Accordingly, in choosing planting locations, we expect to find that recreational benefits will be optimised not only by planting in locations where individuals enjoy the greatest welfare gains from new woods but, at least as importantly, in locations where many people can be advantaged by access to the new recreational resource.

The bottom row of maps in Figure 6.2 show an equivalent analysis but this time for the second scenario of a new 100ha woodland planted at a distance of 20 minutes' drive time from each population location. Comparing with the previous analysis, it is evident that the benefits of a new recreational woodland decline rapidly with increasing travel distance. At 20 minutes distance the average per head annual welfare gains fall to £0.29. The clear message is that in choosing optimal planting locations, recreational values will exert a powerful influence to plant close to heavily populated areas.

Figure 11.2: Recreational welfare benefits from the planting of a 100ha broad-leafed woodland

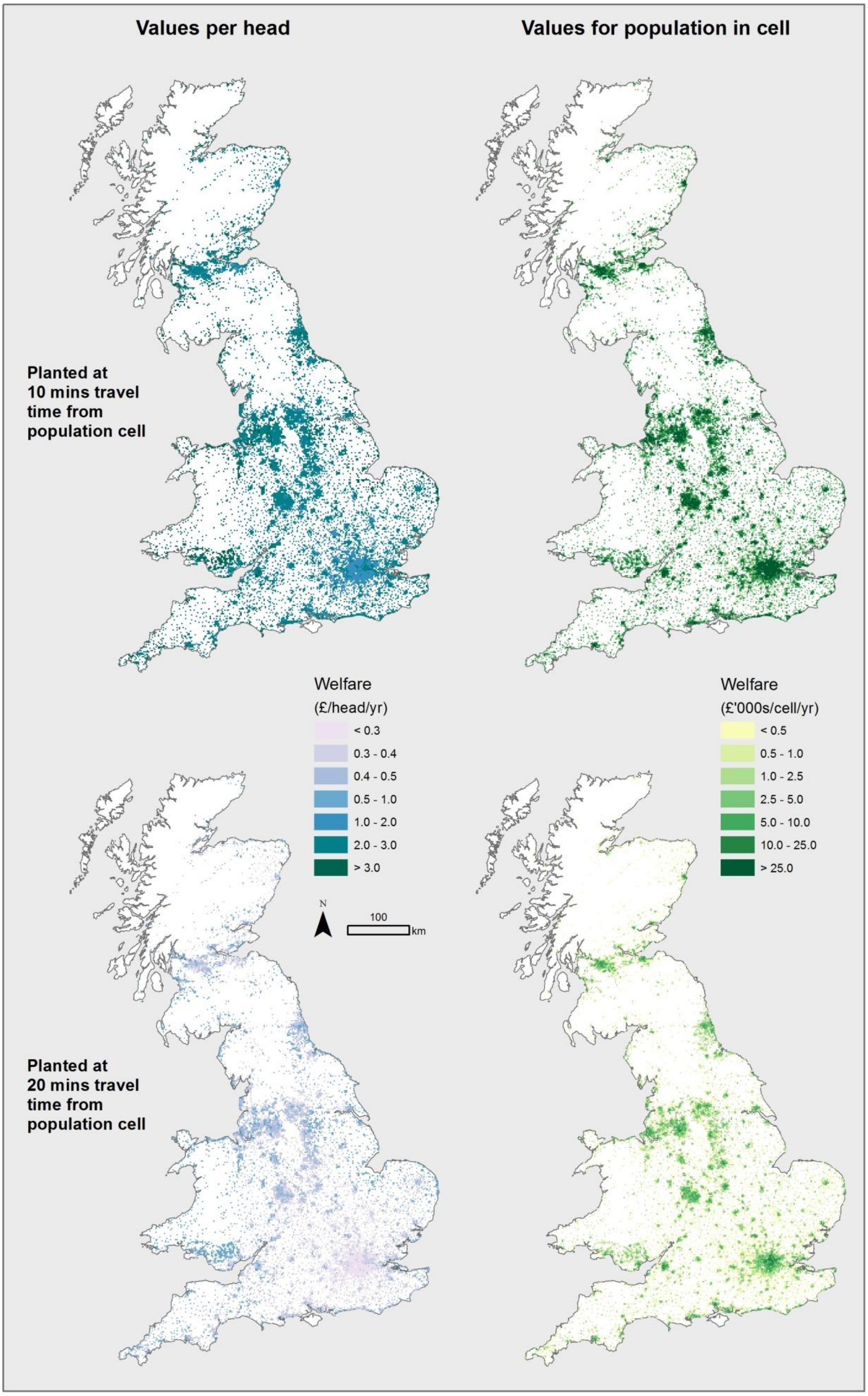

6.6. Concluding Remarks

The recreation model described in this section provides a means of estimating the welfare values that might arise from complex patterns of new woodland planting across GB. Those welfare values are calculated in money terms and our analyses suggest the magnitudes of the welfare gains estimated by the model are of intuitively appropriate proportions: the planting of a substantial 100ha forest at 10 minutes driving distance, for example, results in an average individual welfare gain of £3.02 per year. In addition, that recreational demand model predicts that those welfare gains are lower the more distant the newly planted woodland: at a distance of 20 minutes driving time that same 100ha woodland only yields average individual welfare gains of £0.32 per year. Finally, intrinsic to the structure of the model is the fact that the welfare gains from a new woodland are less substantial the greater the availability of alternative recreational opportunities: the same 100ha forest planted at 10 minutes driving distance, for example, offers an annual welfare gain of £4.65 for each individual in the worst endowed area and only £1.14 in the best endowed area.

Much of the effort in constructing the recreational demand model has been in compiling a suitably comprehensive dataset of outdoor recreational sites. That enormous undertaking has resulted in perhaps the richest dataset of recreational choices ever compiled for the UK, indeed, perhaps the most comprehensive constructed anywhere in the world. Within the time constraints imposed by the NEA-FO, we have only been able to exploit a tiny fraction of that richness and the possibility exists to develop truly exceptional recreational demand models based on this initial effort.

7: The biodiversity module

7.1. Summary

The biodiversity module relies on a model of bird diversity, developed using Breeding Bird Survey (BBS)[9] data collected at a 1km square resolution during the period 1999 –2011. These data were related to land use data from this period, together with various other predictors. Diversity was modelled for various categories of birdlife: (i) all species; (ii) farmland birds (of particular interest given declines in this group); (iii) woodland and upland habitat birds; (iv) birds on the red and amber lists of conservation concern (84); (v) birds on the green list (those not of conservation concern). Various combinations of these categories were also considered (e.g. red and amber list farmland species). Whilst some estimates lacked precision, patterns emerged with regard to the impacts of land use upon biodiversity. Habitat-specific constraints for upland, farmland and woodland areas are suggested.

7.2. Objective

The objective of this module is to develop a model of the impact of land use and land use change on the diversity of breeding birds across Great Britain. This is integrated into the overall NEV modelling suite and used to examine the impact of land use change and constraints upon measures of biodiversity.

7.3. Data

The Breeding Bird Survey (BBS) is a line-transect survey of a random sample of 1km squares across GB, collected annually by volunteers on behalf of the BTO, JNCC and the RSPB. Sample squares are chosen as a random sample, stratified by observer density: Regions with larger numbers of potential volunteers are thereby allotted a larger number of squares, enabling more birdwatchers to become involved in these areas. The analysis is weighted appropriately to take the differences in regional sampling density into account, as described below. Observers make two early morning visits to a given sample square between April and June, recording all birds encountered while walking two 1-km transects across the square. Birds are recorded in three distance categories, or as ‘in flight’. The aim is for each volunteer to survey the same square (or squares) every year (85).

BBS data for the years 1999 – 2011 were obtained (85) to correspond with the earliest and latest years for which land use data were available (*c.* 2000 and *c.* 2010). There are no BBS data available for 2001 due to access restrictions arising from the foot-and-mouth outbreak. Dictated by available records for agricultural land use data, analyses were conducted with respect to “early land use”, using bird data from 1999 to 2005, and “late land use” using bird data from 2006 to 2011. Analyses focused, therefore, on two ranges of years, referred to hereafter as “early” and “late”. Figure 7.1 shows the distribution of BBS 1km squares surveyed in Great Britain during the period considered. Land use data (see Table 1.1) at 1km resolution for the same locations were also used in the analyses.

---

[9] The BBS is jointly funded by the British Trust for Ornithology (BTO), the Joint Nature Conservation Committee (JNCC) and the Royal Society for the Protection of Birds (RSPB).

Figure 7.1: Distribution of Breeding Bird Survey (BBS) 1km squares surveyed between 1999 and 2011 with available 1km land use data.

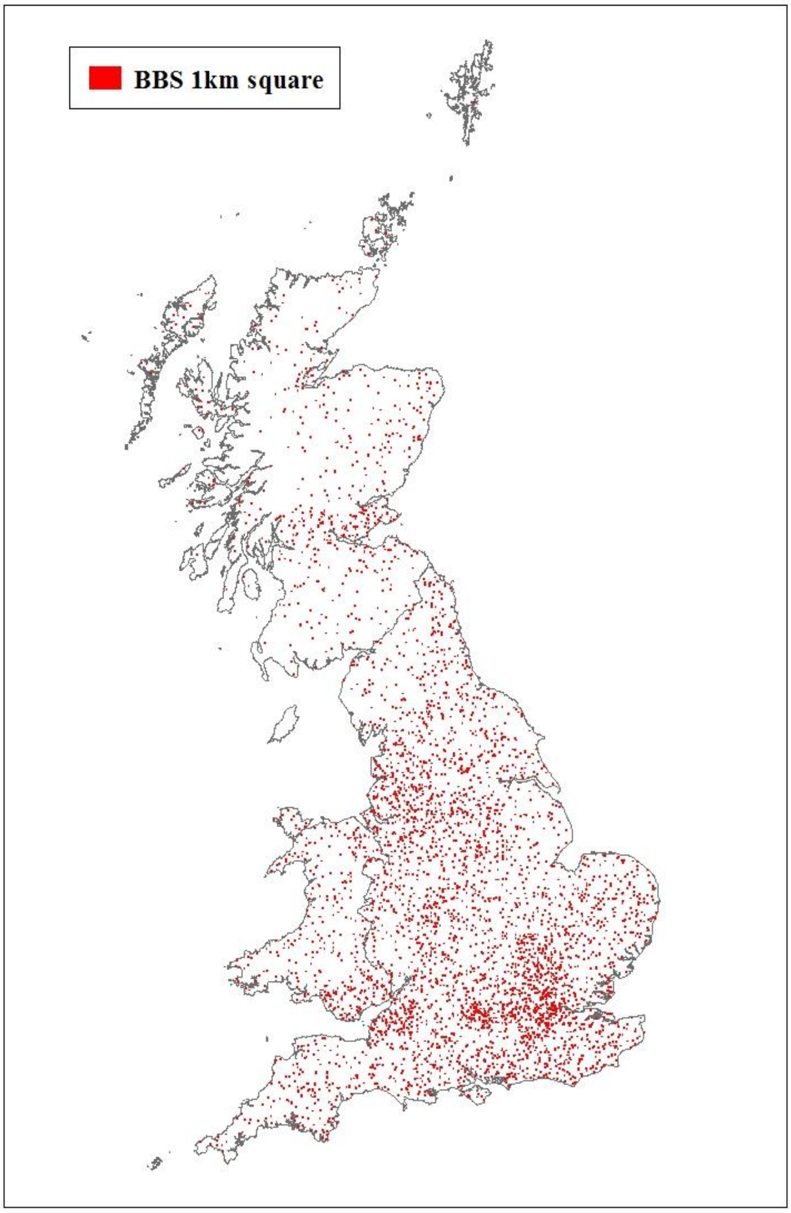

7.4. Methodology

BBS count data were processed in order to extract the most robust summary of the breeding bird community present in each 1km survey square. Breeding birds are easier to survey repeatedly due to territoriality and/or close association with nesting locations. Non-breeding birds, either wintering populations or young yet to reach breeding age, are much less predictable in numbers, aggregation and location in the landscape, requiring both different survey methods and different analytical approaches for spatio-temporal variation to be assessed. For these reasons, there is no analogue of the BBS for non-breeding birds. In the context of this report a breeding bird focus is appropriate because model robustness and, therefore, reliability is maximized, and because it makes efficient use of the datasets available for analysis. It is possible that future analyses may be able to incorporate data from other

surveys, taking into account non-breeding and wintering bird populations in wetlands, for example.

Bird data were summarized within each of the early and late year ranges in order to minimize possible effects of stochasticity in annual counts from these low-intensity sample surveys.

Records of birds in flight were discarded, as these individuals were not closely associated with the habitat within the cell. This helps to ensure that data contributing to the diversity indices were more likely to reflect direct influences of the habitat within the squares in question, such that changes in these habitats are reflected more accurately in the predictions. Squares with data from only one year within the 1999-2011 time periods were discarded, as were records of bird species that were recorded on fewer than 40 BBS squares across the country and full time period. For each species, the maximum count across both visits in a year was extracted.

Any unusually high, outlier bird counts (totals of birds not recorded as in flight) for each square-species combination were excluded because they probably represented non-breeding flocks. Flocks were identified and excluded as follows for all species: if a species had a ratio of maximum to median count of over 20, taking early and late visit counts into account across the whole BBS dataset, the counts greater than the 99th percentile were flagged. If one of the two counts from a given year were flagged in this way, the other, lower count was used and the flagged value discarded. If both counts were greater than the 99th percentile, then the lower value was used, unless both counts were greater than twice the value of the 99th percentile, in which case no count for that species was included for that square in that year (note that the latter occurrence was extremely rare). This process aimed to exclude records that were unreliable as indices of local breeding densities whilst retaining genuine extreme values that are likely to be informative of bird communities in unusual habitats. After this process, the maximum of the remaining early and late counts for a given square in a given year was taken as the count for that square and year.

The composition of the bird community represented by the presence and abundance of all remaining bird species in each survey square and year range was summarized using Simpson's Diversity Index (*D*) (86), calculated following Equation 7.1.

Equation 7.1:

$$D = \frac{1}{\sum_{i=1}^{S} p_i^2}$$

where S = number of bird species recorded at a focal site in that year, p_i = proportion of birds of species i relative to the total number of birds of all species.

The maximum value of D was calculated for each square across all years within each year range in which that square was surveyed. This became the dependent variable in the models. The maximum of these annual counts within a year range was then taken as the "early range" or "late range" count for that square, as appropriate.

In order to observe the effect of land use on different elements of the community of birds found in a 1km square, Simpson's diversity index was calculated using: (i) all species, (ii) only

species which are known to occur on farmland and farmland borders, (iii) those deemed to be woodland specialists or generalists (87), (iv) those species found in upland habitats, (v) those species that are red- or amber-listed and thus of conservation concern, (vi) those green-listed so not deemed to be of conservation concern (84). A further four species groups were produced by sub-dividing the latter two categories further into the farmland or woodland species on the red and amber, or green, lists.

For each 1km BBS grid square, the land use, land cover and livestock datasets dictated the possible explanatory variables. The variables selected were based on: a) expert knowledge of bird habitat preferences; b) limiting variables with uneven reporting rates across Great Britain (e.g. seasonality in agricultural data, see Table 1.1); and c) reducing the presence of correlated variables (e.g. cattle and sheep were highly correlated with permanent grassland). Additionally, coastal habitat was rare on BBS squares and was dropped due to low sample size. Due to the large, known differences in bird communities between deciduous and coniferous woodland the distinction between these was incorporated by using the deciduous and coniferous cover variables found in the input datasets (see Table 1.1).

In Great Britain, there are local area differences in the composition of bird communities which do not always relate to the presence or absence of a particular habitat (at least to the extent that such habitat is distinguishable using this land use definition). Following the approach used in UK-NEA (106), to avoid the spurious relationships with particular habitat categories that broad spatial patterns might produce, the 100km Ordnance Survey grid square corresponding to each BBS square was included in the model as a factor. Due to the paucity of BBS 1km squares in a number of 100 km squares, some adjacent grid squares were combined so that each level of this control variable contained at least 15 BBS squares.

General Linear Models were run using the GENMOD procedure in SAS (88). Data from both year ranges were included together in single models. This could have introduced a degree of pseudo replication and inaccurate estimates of variance with greater precision than was justified by the data. As a conservative estimate, therefore, standard errors were derived as the maximum standard error for each parameter from models run using either only early or only late range data. Models were fitted using every possible combination of the 12 land use variables; squared terms were always fitted with the corresponding linear term. The 100km square identity variable was included in every model. In order to account for the variable survey effort across the UK introduced by the stratification of the BBS sample and, thus, to ensure that the model results were equally applicable to all parts of the UK, an appropriate weighting variable was included in every model, as follows. The country was divided into the standard regions used in the organisation of the BBS (N=80), the total number of BBS squares surveyed during each year being divided by the number of squares surveyed in that region during the same year to provide an annual weight value for each square surveyed. The weight value for each square used in the models was then the mean weight value across the years in which that square was surveyed for each range of years, either between 1999 and 2005 or 2006 and 2011.

The Akaike Information Criterion (AIC) value was calculated for each model, with the lowest value across models showing the most parsimonious model, balancing explanatory power against the number of parameters. Akaike weights were calculated for each variable and model-averaged parameter estimates calculated for each variable, squared term, level of the 100km factor and intercept along with model averaged standard errors, as per Burnham and Anderson (89, 90).

7.5. Results

Akaike variable weights are shown in Table 7.1 for the diversity of all birds, with 1 representing the variables given the highest ranking in calculating model averaged parameter estimates. Values were similar for the other diversity variables. The weights show that all the variables, with the exception of potatoes, horticulture and other crops, were important in explaining the variation in bird diversity nationally. “Other crops” may have been too heterogeneous in composition to have a consistent effect over large spatial scales, while the same might be true of potatoes and horticulture they may still be influential land-uses locally.

Table 7.1: Model-averaged Akaike weight for land use variables, overall Simpson’s diversity index.

Variable	Model-averaged Akaike weight
Deciduous woodland	1.000
Coniferous woodland	1.000
Fresh water	0.982
Urban	1.000
Permanent grassland	1.000
Rough grazing	1.000
Non-farmed grassland	1.000
Wheat	1.000
Barley	1.000
Other cereal	0.201
Potatoes	0.687
Horticulture	0.562

Model fits were acceptable, although lower than would be ideal to support the use of the models for predictions: observed-to-predicted-value correlation coefficients varied from 0.53 to 0.70 (0.60 for the diversity of all birds). This indicates that the models have considerable predictive value, but that they also leave a significant proportion of the variation in diversity unexplained.

The model averaged parameter estimates and associated standard errors are shown in Tables 7.2 to 7.4 for all Simpson’s diversity indices for the intercept and all land use variables. For illustration, Table A7.1 in Annex A7 shows the parameter estimates and standard errors for the 100 km square factor for the diversity of all birds. High standard errors, suggest that regional effects on diversity, independent of land-use differences, were weak in most cases. The limited importance of the potatoes, horticulture and other crops variables is reflected in the high model-averaged standard errors relative to the parameter estimates for these three variables (Tables 7.2 to 7.4).

Table 7.2: Land use variable model-averaged parameter estimates for models with pooled data and standard errors as maximum of early or late range data, dependent variables are Simpson's diversity indices with different bird species communities.

Model-averaged parameter estimate (SE)				
Variable	All species	Farmland species	Farmland red- and amber-list species	Farmland and green-list species
Intercept	16.4301 (1.7469)	10.5509 (1.1634)	5.8858 (0.5639)	6.9949 (0.5530)
Deciduous	0.1224 (0.0217)	0.0773 (0.0136)	0.0189 (0.0072)	0.0612 (0.0075)
Deciduous^2	-0.0019 (0.0003)	-0.0012 (0.0002)	-0.0004 (0.0001)	-0.0009 (0.0001)
Coniferous	-0.0461 (0.0139)	-0.0240 (0.0091)	-0.0017 (0.0061)	0.0080 (0.0061)
Coniferous^2			-0.0002 (0.0001)	-0.0003 (0.0001)
Fresh water	0.0902 (0.0547)	-0.0213 (0.0325)	0.0053 (0.0162)	-0.0503 (0.0173)
Fresh water ^{2}	-0.0022 (0.0013)	-0.0000 (0.0007)	-0.0006 (0.0005)	0.0008 (0.0004)
Urban	0.0442 (0.0183)	0.0211 (0.0114)	-0.0005 (0.0056)	0.0250 (0.0056)
Urban^2	-0.0011 (0.0001)	-0.0006 (0.0001)	-0.0003 (0.0001)	-0.0005 (0.0001)
Perm grassland	0.0320 (0.0249)	0.0173 (0.0172)	0.0111 (0.0085)	0.0028 (0.0111)
Perm grassland^2	-0.0006 (0.0002)	-0.0003 (0.0001)	-0.0002 (0.0001)	-0.00005 (0.0001)
Rough grazing	-0.1297 (0.0152)	-0.0726 (0.0097)	-0.0313 (0.0043)	-0.0462 (0.0042)
Rough grazing^2				
NF grassland	-0.0931 (0.0175)	-0.0548 (0.011)	-0.0096 (0.0089)	-0.0352 (0.0057)
NF grassland^2			-0.0004 (0.0002)	
Wheat	0.0016 (0.0381)	0.0592 (0.0249)	0.0460 (0.0126)	0.0304 (0.0118)
Wheat^2	-0.0010 (0.0005)	-0.0013 (0.0004)	-0.0011 (0.0002)	-0.0008 (0.0002)
Barley	-0.0295 (0.0379)	0.0238 (0.0272)	0.0247 (0.0140)	-0.0212 (0.0160)
Barley^2	-0.0007 (0.0009)	-0.0014 (0.0007)	-0.0009 (0.0004)	-0.0001 (0.0004)

Other cereal	0.0187<br>(0.1790)	0.0768<br>(0.1231)	0.0044<br>(0.0544)	0.0605<br>(0.0758)
Other cereal^2	-0.0017<br>(0.0216)	-0.0043<br>(0.0129)	-0.0005<br>(0.0072)	-0.0035<br>(0.0078)
Potatoes	-0.0610<br>(0.1471)	-0.1012<br>(0.0853)	0.0045<br>(0.0418)	-0.0645<br>(0.0456)
Potatoes^2	-0.0002<br>(0.0101)	0.0010<br>(0.0065)	-0.0006<br>(0.0037)	-0.0004<br>(0.0040)
Horticulture	-0.0325<br>(0.0564)	-0.0044<br>(0.0292)	-0.0002<br>(0.0215)	-0.0161<br>(0.0223)
Horticulture^2	0.0005<br>(0.0013)	0.0002<br>(0.0009)	0.00002<br>(0.0006)	0.0005<br>(0.0006)

Note: NF = non-farm

Table 7.3: Land use variable model-averaged parameter estimates for models with pooled data and standard errors as maximum of early or late range data, dependent variables are Simpson's diversity indices with different bird species communities.

Model-averaged parameter estimate (SE)			
Variable	Woodland species	Woodland red- and amber-list species	Woodland and green-list species
Intercept	7.1114 (0.7466)	3.128 (0.3404)	5.7328 (0.4988)
Deciduous	0.1462 (0.0093)	0.0357 (0.0041)	0.1205 (0.0069)
$Deciduous^2$	-0.0015 (0.0002)	-0.0004 (0.0001)	-0.0013 (0.0001)
Coniferous	0.0706 (0.0079)	0.0059 (0.0036)	0.0546 (0.0055)
$Coniferous^2$	-0.0006 (0.0001)	-0.00005 (0.00003)	-0.0004 (0.0001)
Fresh water	-0.0185 (0.0290)	0.0027 (0.0058)	-0.0203 (0.0201)
Fresh water ^{2}	0.0007 (0.0007)		0.0006 (0.0006)
Urban	0.0244 (0.0072)	0.0056 (0.0033)	0.0162 (0.0052)
$Urban^2$	-0.0004 (0.0001)	-0.0001 (0.0000)	-0.0003 (0.0000)
Perm grassland	0.0265 (0.0138)	0.0010 (0.0060)	0.0147 (0.0105)
Perm $grassland^2$	-0.0002 (0.0001)	-0.000002 (0.0001)	-0.0001 (0.0001)
Rough grazing	-0.0412 (0.0056)	-0.0111 (0.0029)	-0.0303 (0.0040)
Rough grazing ^{2}			
NF grassland	0.0081 (0.0123)	0.0064 (0.0062)	0.0005 (0.0091)
NF $grassland^2$	-0.0007 (0.0003)	-0.0003 (0.0002)	-0.0004 (0.0002)
Wheat	0.0285 (0.0174)	-0.0015 (0.0069)	0.0274 (0.0129)
$Wheat^2$	-0.0007 (0.0003)	-0.0002 (0.0001)	-0.0006 (0.0002)
Barley	0.0098 (0.0256)	-0.0035 (0.0085)	0.0063 (0.0185)
$Barley^2$	-0.0002 (0.0006)	0.00001 (0.0002)	-0.0002 (0.0004)
Other cereal	0.1419 (0.0897)	0.0005 (0.0272)	0.1256 (0.0615)

Other cereal^2	-0.0103<br>(0.0092)	0.0001<br>(0.0033)	-0.0097<br>(0.0061)
Potatoes	-0.0725<br>(0.0552)	-0.0374<br>(0.0128)	-0.0379<br>(0.0429)
Potatoes^2	-0.0004<br>(0.0050)		-0.0008<br>(0.0040)
Horticulture	0.0031<br>(0.0128)	-0.0010<br>(0.0052)	0.0022<br>(0.0070)
Horticulture^2			

Table 7.4: Land use variable model-averaged parameter estimates for models with pooled data and standard errors as maximum of early or late range data, dependent variables are Simpson's diversity indices with different bird species communities.

Model-averaged parameter estimate (SE)			
Variable	Upland species	Red and amber list species	Green list species
Intercept	2.0959 (0.316)	8.3375 (0.7996)	10.6097 (0.8992)
Deciduous	-0.0072 (0.0029)	0.0064 (0.0120)	0.1110 (0.0123)
Deciduous^2		-0.0004 (0.0002)	-0.0015 (0.0002)
Coniferous	-0.0121 (0.0037)	-0.0014 (0.0089)	0.0169 (0.0100)
Coniferous^2	0.0001 (0.00004)	-0.0004 (0.0001)	-0.0005 (0.0001)
Fresh water	0.0449 (0.0094)	0.064 (0.0235)	0.0069 (0.0142)
Fresh water ^{2}	-0.0008 (0.0003)	-0.0014 (0.0005)	
Urban	-0.0095 (0.0027)	-0.0126 (0.0080)	0.0509 (0.0094)
Urban^2		-0.0003 (0.0001)	-0.0008 (0.0001)
Perm grassland	0.0157 (0.0044)	0.0248 (0.0113)	0.0094 (0.0154)
Perm grassland^2	-0.0002 (0.0000)	-0.0005 (0.0001)	-0.0002 (0.0002)
Rough grazing	0.0213 (0.0043)	-0.0596 (0.0059)	-0.0738 (0.0069)
Rough grazing ^{2}	-0.0003 (0.00004)		
NF grassland	-0.0031 (0.0039)	-0.0109 (0.0127)	-0.0556 (0.0093)
NF grassland^2		-0.0006 (0.0003)	
Wheat	-0.0312 (0.0075)	-0.0138 (0.0178)	0.0255 (0.0193)
Wheat^2	0.0004 (0.0001)	-0.0004 (0.0003)	-0.0010 (0.0004)
Barley	-0.0190 (0.0084)	0.0072 (0.0212)	-0.0522 (0.0227)
Barley^2	0.0002 (0.0002)	-0.0005 (0.0006)	0.0003 (0.0006)
Other cereal	-0.0157 (0.0349)	-0.0015 (0.0795)	0.0299 (0.1090)

Other cereal^2	0.0007 (0.0033)	-0.0005 (0.0112)	-0.0021 (0.0137)
Potatoes	-0.0280 (0.0304)	0.0039 (0.0615)	-0.0921 (0.0740)
Potatoes^2	0.0029 (0.0030)	-0.0003 (0.0061)	-0.0005 (0.0064)
Horticulture	-0.0200 (0.0116)	-0.0048 (0.0295)	-0.0042 (0.0168)
Horticulture^2	0.0002 (0.0003)	-0.0000 (0.0009)	

7.6. Discussion and conclusions

Considering results for the overall diversity index (Table 7.2), deciduous woodland has one of the largest estimated effects of land use upon bird biodiversity. The substantial positive linear effect combined with the smaller negative squared term suggest that increasing such woodland raises diversity although the rate of increase flattens off at higher levels. There is a negative linear effect for coniferous woodland, emphasising the importance of the difference between woodland compositions for bird diversity. Freshwater displays a similar shaped relationship to that of deciduous woodland (although at a lower effect size). A wider array of waterbirds will occur where fresh water is present in an area, alongside other habitats that will provide for a broad range of terrestrial species, although higher areas of fresh water will generally have less of the species-rich edge habitat that is particularly rich in resources for birds. Urban habitats show a positive polynomial trend, perhaps aided by the presence of garden habitat. Permanent grassland shows a similar trend, albeit with a rather high standard error. Negative estimates for rough grazing and non-farmed grassland may be related to the prevalence of these habitats at higher altitudes where diversity tends to drop off. The presence of such correlations implies that parameters should be interpreted with some care and not unduly extrapolated out of sample. Estimates for wheat and barley are quite low and have high associated standard errors, reflecting the species-paucity of large tracts of arable land, despite the presence there of significant numbers of species of conservation concern.

The results for farmland birds illustrate the complexity in interpreting broad patterns in a summary index such as diversity. Estimates for barley are quite low and with high standard errors, indicating no positive effect of this crop despite its common use in less intensive farming regimes that are typically beneficial to farmland birds. Conversely, there appears to be a positive relationship with wheat, albeit dropping off where it becomes most common, despite this crop being indicative of intensive arable cropping. This pattern is still observed whether species are of conservation concern or not. This apparent contradiction probably reflects the fact that, despite long-term declines, farmland birds are still most common in regions dominated by farmland, while more extensive systems are now mostly found in marginal farming areas where factors such as climate and non-cropped habitats may have more influence on the presence of farmland birds. Relevant non-cropped habitats include the hedgerows found in much arable farmland, versus the dry-stone walls often found in more marginal areas.

Woodland species are more diverse in deciduous woods and this trend is stronger, with smaller standard errors indicating more certainty in the conclusion, than it is for diversity as a whole. However, coniferous woodland also shows a positive relationship for this category of species,

indicating the importance of this tree community for a largely distinct group of bird species. However, while this trend is positive, it is curvilinear and peaks at an intermediate level of conifer area, showing that the benefits for diversity are maximized when it is found in combination with other habitats. The pattern is also strong only for overall woodland bird diversity and species on the green list, not for those considered of conservation concern, i.e. the effects mostly concern common species.

Upland bird diversity has a negative relationship with deciduous woodland and predominantly negative relationships, becoming less steep at higher areas, with coniferous woodland, wheat and barley. These effects show negative associations with land-uses not found in the uplands, while predominantly positive associations with fresh water and rough grazing (which is correlated with the mountains, moorlands and heathland land cover variable) reflect the habitat preferences of upland species.

When all birds on the red or amber list are considered, fresh water and permanent grassland appear predominantly to influence diversity positively, although the relationships level off at higher area cover values. Other variables, including deciduous woodland, show less clear effects on birds of conservation concern, but, for birds on the green list, deciduous woodland still shows the greatest effect on diversity, as found for the overall diversity index. However, such patterns should be interpreted with caution, because land-use variables may actually only be correlated with the true drivers of variation, rather than causal factors, and diversity are complex composite variables that will be influenced in multiple ways.

All of the results described above and, ultimately, used in land use change analyses to assess effects on biodiversity, concern changes in the Simpson's diversity index for breeding birds. Although the method for calculating this index is described in the methodology, the absolute figures for the index do not have an easily visualized meaning.

7.6.1. Constraints and caveats

The production of multiple indices allows for the possibility of a more nuanced approach to biodiversity constraints, sensitive to the dominant species and habitats of the area in question. The bird communities in uplands, for example, may change with encroachment of farmland or woodland leading to a rise in overall diversity, but this might mask a reduction in the diversity of more specialist upland birds, which would not be desirable from a conservation viewpoint. Equally, where a landscape is dominated by farmland, then maintenance of farmland bird diversity might be a target, and if there are substantial areas of woodland, woodland bird diversity is likely to be more important than a simple, overall diversity index. With this in mind, we suggest the following rules for land use constraints, which would be spatially specific, defined in respect of landscape type. We would propose that a "change" (in all cases below, "*falls*") would be defined as having to be significant, i.e. with 95% confidence limits excluding zero. In each case below, we suggest an appropriate constraint that could be defined from the data analysis described above for a particular landscape type, defined by default at the 1km square scale. If, on predicting bird diversity responses from land-use change and the models described here, the response would violate the constraint described, the land-use change concerned would be deemed unacceptable.

- In uplands, defined as where mountain, moors and heathland (MMH) constitute 50% or greater of the land cover of an area, neither upland diversity nor overall diversity of red- and amber-listed birds should *fall*.
- In farmland, defined as where total farmland (farmland plus improved grassland) constitutes 50% or greater of the land cover of an area, then neither farmland diversity nor overall diversity of red- and amber-listed birds should *fall*.
- In woodland, defined as where total woodland (deciduous plus coniferous woodland) constitutes 15% or greater of the land cover of an area, then neither woodland diversity nor overall diversity of red- and amber-listed birds should *fall*.
- In lowland mosaic landscapes, defined as where total woodland constitutes 15% or greater and total farmland constitutes 50% or greater of the land cover of an area, none of farmland diversity, woodland diversity and overall diversity of red- and amber-listed birds should *fall*.
- Where none of the above applies, then neither overall diversity nor diversity of red- and amber-listed birds should *fall*.

If any of the above diversity losses are predicted to apply within an area following land use change, then the value of the lost economic activity from imposing the constraint which avoids those losses provides us with an estimate of the 'opportunity cost' of maintaining biodiversity. The option which minimizes those opportunity costs is referred to as the cost-effective solution.

7.6.1.1. Caveats

A number of caveats should be taken into account when interpreting the maps, the models presented, and the summaries of bird diversity predicted under each scenario. These are summarised below considering the source data and the model used:

- **BBS survey design and data handling**. BBS surveys focus on terrestrial breeding birds, so coastal and estuarine birds tend to be under-recorded and make up only a small proportion of the diversity modelled here. Birds which are normally observed in flight are likely to be under-recorded as these observations are discarded from this analysis. The number of BBS squares covered in upland habitats is limited due to problems of accessibility for volunteers, so the results for these areas are under-represented compared to lowlands. Given the conservation importance of retaining upland bird communities rather than allowing generalists (common species which inhabit multiple environments) to colonise the uplands, which could increase overall diversity, we have retained this index for use in the constraints. The number of species contributing to the diversity index was limited to those that were recorded in 40 or more squares. Variations in the detection probability between species and habitats were no accounted for in the analyses described here. Therefore, variation in diversity may be underestimated.

- **Consistency of habitat relationships with scale**. Birds may differ in their habitat preferences depending on the scale of the change in habitat. The model derived here, as it stands, is assessing only the effect of habitat cover at the 1km square scale, but the larger effect sizes of the 100km square variable suggests that larger-scale factors may be more important. The scale at which diversity is measured must be considered if it is to be used to underlie management decisions: aiming to maximize diversity at a local scale will very often give rise to different recommendations to maximizing it at larger

scales. The percentage cover of habitats determining the constraints may have to be adjusted at different scales and this could be a focus of future research.

- **Subtle changes and other drivers.** Important effects on biodiversity could easily occur through subtle changes in land-use which are not included in these models. Whilst it is important to assess the effect of land use change on bird communities, hence the use of diversity indices here, there will be important sensitivities at species-level which will be missed in this analysis due to the lumping of effects for multiple species. This may potentially lead to management which might be good for a community of birds overall but bad for particular species, which may be of conservation concern themselves. Future work intends to look in more detail at species-level effects. Finally, the analysis here models only the effect of land use change on bird species diversity. Direct effects of climate, for example, could have major impacts on bird distribution independent of their effects on land-use.

  Model fit. The models of Simpson's diversity index described here explain reasonable proportions of the observed variation, but less than would be ideal. It is likely that this is because diversity is a complex, multi-faceted variable that is influenced by assemblage composition and the relative abundances of all species present, as well as the coarse nature of the land-use variables used. As a result, caution should be used in interpreting predictions of changes in diversity resulting from applications of the models. On-going work is investigating the models based on the abundance of individual species and the use of less amalgamated and enhanced land-use data. These models will be used to construct diversity indices as a secondary product and are expected to provide greatly improved predictive power.

Annex A7: Regional effects on biodiversity

Table A7.1: Model-averaged estimates for levels of 100km square class, Simpson's diversity for all birds.

Ordnance Survey 100km Square merged region descriptions	Model averaged parameter estimate (Std.Err)
AB	-1.5548 (1.2386)
AR	-0.7253 (1.2079)
CO	-0.4816 (1.4215)
CU	0.0762 (1.2291)
DY	-0.7001 (1.2267)
GL	-0.4303 (1.1546)
OH	-3.1689 (1.3261)
OS	-1.1752 (1.3274)
NC	-0.4483 (1.2249)
NG	-0.9245 (1.2531)
NH	-0.5495 (1.2104)
NN	-0.6337 (1.2155)
NO	-1.0614 (1.2117)
NS	-1.3374 (1.1810)
NT	-0.6208 (1.2039)
NU	0.4162 (1.6096)
NY	-0.7187 (1.1895)
NZ	-0.1999 (1.2037)
SD	0.3606 (1.1924)
SE	-0.5415 (1.1743)
SH	-1.0573 (1.2618)
SJ	0.3337 (1.1756)
SK	0.0360 (1.1508)
SO	-0.0577 (1.1764)
SP	-0.8994 (1.1529)
SS	-1.0692 (1.3072)
ST	-0.0263 (1.1687)
SU	-0.7647 (1.1388)
SX	-0.5799 (1.2273)
SY	-0.1932 (1.4505)
SZ	-1.4332 (1.7712)
TA	-1.6052 (1.2173)
TF	-1.1068 (1.1848)
TG	0.2882 (1.3868)
TL	-0.0432 (1.1586)
TM	-0.8741 (1.2631)
TR	0.000 (0.0000)

8. The Natural Environment Valuation (NEV) Integrated Model

8.1. Overview

The individual component modules and their linkages are programmed together through our custom-built software system; The Natural Environment Valuation (NEV) model. This programmed linkage allows the analyst to examine the consequences of any desired change in multiple drivers. For example, NEV allows the analysis to examine the consequence for land use of say a new farm subsidy regime at the same time as a shift in precipitation and temperature arising from climate change. NEV traces the consequences of these changes through the component modules to yield estimates of both direct impacts in terms of land use and agricultural produce, and indirect effects upon alternative land uses (e.g. a reduction in woodlands), changes in GHG emissions, recreation and biodiversity. All of these effects are assessed in quantitative terms and all except for biodiversity are measured in terms of economic values.

The major advantage of NEV is not in terms of the assessment of the effects of some user-specified policy change, useful though that is. Rather, the major innovation here is the potential which NEV affords to identify the optimal way in which to implement such a policy change. This is achieved by using NEV to interrogate all the component modules simultaneously to examine the consequences of any specified change in some land use driver occurring at any location and at any time over a specified period. Through an optimisation routine NEV identifies those solutions which maximise and user-defined objective. So, as in the Flat Rate payment analysis, NEV allows us to maximise the market value of a land use policy. Given that the current UK baseline is that a shift from conventional agriculture to woodland typically reduces market values then this is identical to minimising that loss i.e. identifying the set of farms whose subsidy requirements are lowest. These will be those farms which face the lowest opportunity cost of giving up their existing (low value) agricultural production and accepting subsides to plant trees. In the Natural Capital application NEV considers a much wide set of the consequences of land use change, extending beyond the simple market values of lost agricultural output and the value of timber production to additionally consider the major non-market consequences of converting farmland to woodland. This requires assessment of the biodiversity, greenhouse gas and recreational consequences of that land use change. In so doing the analysis illustrates a ‘spatial targeting’ approach to decision making which allocates scarce resources to those locations which maximise a specified objective. This approach avoids the problem of specifying pre-set end points through the Scenario Analysis. As the component modules all reflect the underlying variation of the natural environment and its consequences for economic costs and benefits, this approach genuinely incorporates the natural world into economic decision making.

8.2. A modular, programmed environmental-economic integrative analysis.

The individual component modules and their linkages are programmed together within the NEV software system. An immediate advantage of having an integration system which is linked to but distinct from the component modules is that this permits development of the overall system to evolve even when work is focussed upon just a single module. More fundamentally, modularity raises the potential for other users to substitute alternative models of a given element (e.g. other water quality models) to take advantage of the remainder of the integrated system. The system is constructed in open-source code wherever possible with the

intention being to move towards an entirely open-source system which we would share freely so as to enhance general use of the research and its modelling.

The component modules are constructed so as to simulate the effect of changes in the diverse environment, policy, economic and social forces which drive each system. Figure 8.1 provides an illustrated overview of the NEV decision support system. Here, baseline data provides models of the current status of each component module with each module linked from those that directly impinge upon it and to those which it effects.

Figure 8.1: Illustrated overview of the modules and connections comprising the NEV decision support system

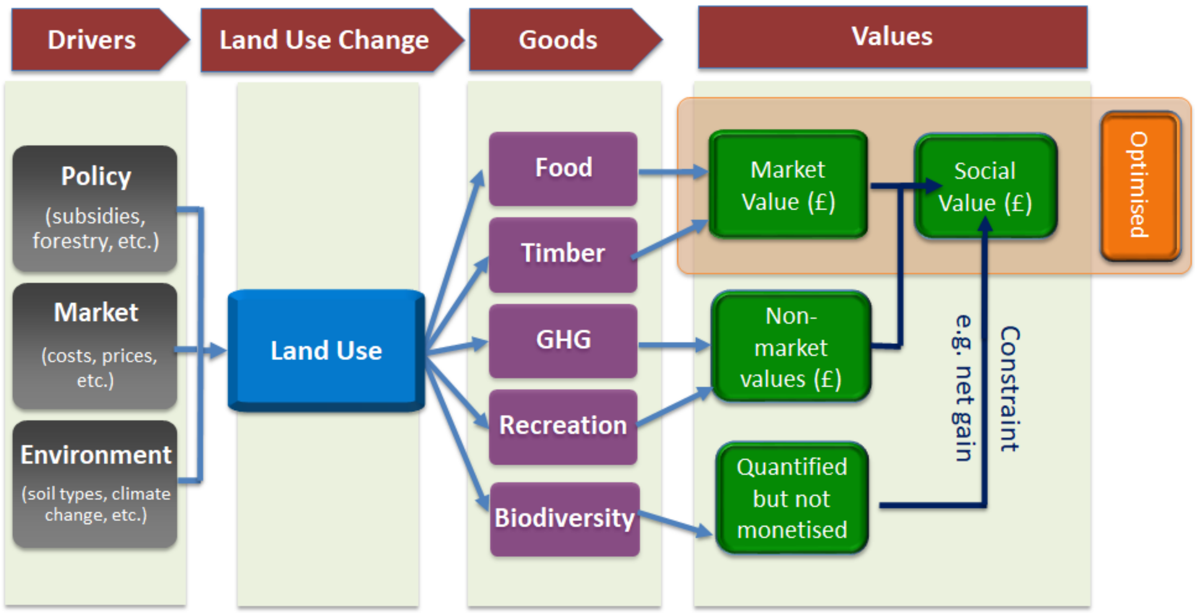

The land use model is acted upon by three sets of drivers: (i) Policy drivers such as land use subsidies, regulations on permitted uses, etc.; (ii) Market drivers, such as the price of crops, the costs of fuel and other inputs, etc.; and (iii) Environment drivers including spatially variable factors such as soil type and temporally variables drivers such as climate change. Changes in one or more of these drivers (e.g. an alteration in farm subsidies in conjunction with on-going climate change) impacts directly upon land use. The underlying modelling here is econometric as described in the agricultural land use model presented previously. It recognises that, within the limits imposed by variation in the three sets of drivers (e.g. physical environment constraints economically precludes certain land uses in certain locations such that wheat cropping of the top of bare rock mountains will yield negative profits), there is a local decision maker (e.g. the farmer) deciding land use in a way to satisfy a self-determined objective. That objective is empirically determined through our analysis of prior land use decisions across GB but roughly relates to an attempt to maximise profit given imperfect knowledge and risk aversion (96; 15, 44; Fezzi et al., 2014, 2015; 23). As policy, market and environmental drivers alter so farmers and other decision makers alter land use (typically with a lag) and consequent produce.

The systems nature of the environment means that this land use change induces responses in all connected systems and these effects are captured in the NEV modules. So a shift in agricultural land use causes change in other land uses, either directly (e.g. though afforestation of previous farmed land) or indirectly (e.g. through responses in greenhouse gas emissions or storage, changes in wild species habitat and biodiversity and changes in recreational behaviour). The programmed linkages within NEV yield rapid estimates of all these responses assessed as quantities and, where robust valuation is possible, as economic values (all but the biodiversity effects).

Development of the individual component modules built for this analysis drew upon the diverse data sources described above. These provided data which were spatially referenced and covered an appropriate time period sufficient to allow the incorporation of both location and temporal change effects within models of land use decision making. In summary these included spatially and temporally disaggregated climate variables including average temperature and accumulated rainfall during the growing season obtained from the UK Met Office website and interpolated to match the resolution of our land use data. Estimates of future weather variables were obtained from the UKCOP (91; 92) climate change scenarios. We also include other measures of the physical environment (such as urban extent) from Ordnance Survey (OS) sources which also provided topographic variables from their Digital Terrain Model, while soil characteristics were derived from the 1km master library of the European Soil Database (93).

8.3. Optimisation

The programmed nature of the model connections in NEV permit fast analysis of the consequences of driver change. This combined with the spatial and temporal nature of all modules permits the use of a variety of optimisation techniques which can be applied to maximise some objective. For the Flat-rate payment approach this objective is to maximise the private financial return to land owners of planting some set amount of forestry (in our application 2 million hectares) over some set period.

A readily implemented approach to optimisation is to employ greedy algorithms. In our Flat-rate payment application the greedy algorithm (94) effectively calculates the private financial return of planting woodland on former farmland (note that this excludes urban or unplantable areas) for every possible location across GB. These are then ranked from the highest to lowest value locations and the desired number of cells are chosen for afforestation. This is a defensible approach when the appraisal is limited to private financial values alone where the afforestation of one area does not change the profitability of afforesting another. However, this spatial independence assumption becomes less defensible if we apply this method to the wider appraisal of values in the Natural Capital approach. Here what happens in one location can have a significant effect upon the consequences of change in other locations. A good example of such spatial dependence concerns recreation and its benefit value. Planting a woodland in an area which has little other open access to the environment can generate a substantial recreational benefit. However subsequently doubling the size of that woodland is unlikely to double its recreational value because much of the requirement for outdoor activity is already catered for in that first forest (a phenomena which economists refer to as diminishing marginal value). In effect the first tranche of woodland provides a substitute for the second and we might well find that the increase in benefits would be higher had we relocated that second forest elsewhere.

To combat the real world challenge of site substitution (or complementarity) that arises from spatial dependence NEV modules such as that for recreation explicitly incorporate those effects. The propensity to visit a site becomes a function not only of the attributes of that site but also those of locations around that site – and of alternative locations around the potential visitors home. However this of course means that, when one site is afforested this has the potential for changing (either positively or negatively) the benefits of afforesting another site. These dynamic effects cannot be captured by the static analysis of the greedy algorithm.

To address the dynamics inevitably triggered when a huge area of land is afforested over a long period NEV utilises the processing speed of its interlinked models to employ combinatorial optimisation techniques (95). These take the user defined number or area of sites (here 2 million ha.), divided over the planting period (a decision which itself has dynamic repercussions but for which we simply assume an equal division over time in this study) and calculates all possible combinations of this planting ordering. In each case, as planting progresses the substitution and complementarity effects are calculated, providing an optimisation outcome which maximises the total market and non-market benefits afforded. This approach also permits the inclusion of additional constraints such as the positive net gain outcome for biodiversity discussed in the final analysis of our paper.

9. Summary of the Scenario analysis

The use of Land Use Scenarios as an input to policy development has become both high profile and increasingly prevalent in recent years (97; 98; 99; 100; 101; 102; 103; 104; 105). Of particular relevance to the present study is the Land Use Scenario analysis undertaken as part of the UK-NEA (106) as this focused upon changes to land use, including woodland creation, in our study area and had a major impact upon land use policy. The UK-NEA engaged with a large and diverse group of stakeholders as detailed in Table 9.1 (107). Using methods described in detail in its main report and supplementary papers (108; 109), the UK-NEA produced six scenarios for future land use and tree planting of which the Nature@Work scenario was identified as delivering the greatest level of ecosystem services. We adopt this scenario for comparison with the Flat-rate payment and Natural Capital approaches so as to cast the Land Use Scenario approach in its most favourable light.

Table 9.1: Stakeholders involved in the construction of scenarios

Stakeholder group	Stakeholder
Government Departments:	Department for Environment, Food and Rural Affairs (Defra)
	Defra Executive Review Group (ERG)
	Defra Natural Environment Strategic Unit (NESU)
	Defra Evidence Programme, Horizon Scanning & Futures
	Department for Business, Innovation and Skills (DBIS)
	Welsh Assembly Government
	Natural England, Strategy and Environmental Futures
	DBIS, Government Office for Science,
	Foresight Follow-up to Land Use Futures
Government Agencies:	Environment Agency
	Environment Agency Wales
	Forest Research
	Centre for Human and Ecological Sciences
	JNCC
	CCW
Research Community, Funders and Universities	Oxford University
	Imperial College
	Macaulay Institute
	Centre for Sustainable Water Management,
	Lancaster Environment Centre
	CEH
	UNEP-WCMC
	Natural Capital Initiative
	Cefas
	NERC
	UK-NEA leadership and Advisory Panel
Business and NGO	Waverley Management Consultants
	RSPB
	Sussex Wildlife Trust

References

1. Day, B.H., Owen, N., Binner, A., Bateman, I.J., Cho, F., De-Gol, A., Ferguson-Gow, H., Ferrini, S., Fezzi, C., Harwood, A., Lee, C., Liuzzo, L., Mancini, M. and Pearson, R. (2020) The Natural Environmental Valuation (NEV) Modelling Suite: A Summary Technical Report, *LEEP Working Paper 01/2020*, Land, Environment, Economics and Policy Institute (LEEP), University of Exeter Business School, https://www.exeter.ac.uk/media/universityofexeter/collegeofsocialsciencesandinternationalstudies/leep/documents/workingpapers/LEEP_Working_Paper_01-2020_-_The_NEV_Modelling_Suite_v2.pdf

2. Bateman, I.J., Day, B.H., Agarwala, M., Bacon, P., Baďura, T., Binner, A., De-Gol, A.J., Ditchburn, B., Dugdale, S., Emmett, B., Ferrini, S., Fezzi, C., Harwood, A., Hillier, J., Hiscock, K., Hulme, M., Jackson, B., Lovett, A., Mackie, E., Matthews, R., Sen, A., Siriwardena, G., Smith, P., Snowdon, P., Sünnenberg, G., Vetter, S. and Vinjili, S. (2014) *Economic value of ecosystem services*, Final Report from Work Package Report 3 to the UK National Ecosystem Assessment – Follow-On programme, Defra, London (available at http://randd.defra.gov.uk and UNEP-WCMC http://uknea.unep-wcmc.org).

3. Sen, A., Harwood, A., Bateman I.J., Munday P., Crowe A., Haines-Young R., Brander L., Provins, A., Raychaudhuri, J., Lovett, A., and Foden J. (2014) Economic Assessment of the Recreational Value of Ecosystems in Great Britain, *Environmental and Resource Economics*, 57(2): 233-249, DOI 10.1007/s10640-013-9666-7

4. Fuller, R. M., Smith, G. M., Sanderson, J. M., Hill, R. A., Thomson, A. G., Cox, R., Brown, N. J., Clarke, R. T., Rothery, P. and Gerard, F. F. (2002) *Countryside Survey 2000 Module 7: Land Cover Map 2000 final report*, NERC/Centre for Ecology and Hydrology 100pp.

5. Murphy, J.M., Sexton, D.M.H., Jenkins, G.J., Boorman, P.M., Booth, B.B.B., Brown, C.C., Clark, R.T., Collins, M., Harris, G.R., Kendon, E.J., Betts, R.A., Brown, S.J., Howard, T. P., Humphrey, K. A., McCarthy, M. P., McDonald, R. E., Stephens, A., Wallace, C., Warren, R., Wilby, R., Wood, R. A. (2009), UK Climate Projections Science Report: Climate change projections, Met Office Hadley Centre, Exeter, UK, http://cedadocs.ceda.ac.uk/1320/1/climate_projections_full_report.pdf

6. Morton, D., Rowland, C., Wood, C., Meek, L., Marston, C., Smith, G., Wadsworth, R., Simpson, I.C. (2011) Final Report for LCM2007 - the new UK Land Cover Map. *Countryside Survey Technical Report No. 11/07*, NERC/Centre for Ecology and Hydrology 112pp. http://www.ceh.ac.uk/documents/LCM2007FinalReport.pdf

7. Posen, P., Hutchins, M., Lovett, A., Davies, H. (2011) Identifying the catchment size at which robust estimations of agricultural land use can be made, and implications for diffuse pollution modelling, *Applied Geography*, 31, 919-929

8. Comber, A., Proctor, C., and Anthony, S. (2008) The creation of a national agricultural land use dataset: combining pycnophylactic interpolation with dasymetric mapping techniques, *Transactions in GIS*, 12, 775-791.

9. Gong, X. and Weber, J-L. (2009) Land Cover and Land Use Classifications in the SEEA Revision (UNCEEA/4/11), presented at the Fourth Meeting of the United Nations Committee of Experts on Environmental-Economic Accounting, 24th – 26th June 2009, UN Headquarters, New York, EEA and FAO, New York.

10. SGRPID (2011) *Economic Report on Scottish Agriculture: 2011 Edition*, Scottish Government Rural Payments and Inspections Directorate, UK; https://www.gov.scot/collections/economic-report-on-scottish-agriculture/

11. Lyons, H. (2010) *Methodology for the development of the ADAS land use database 2010*, ADAS, UK, http://edina.ac.uk/agCensus/support/Methodology_1km_2010.pdf

12. HM Treasury (2022) The Green Book: Central Government guidance on appraisal and Evaluation, HM Treasury, London.

13. UK-NEA (2011) *UK National Ecosystem Assessment: Technical Report*, UNEP-WCMC, Cambridge, UK, http://uknea.unep-wcmc.org/

14. Bateman, I.J. and Mace, G.M. (2020) The natural capital framework for sustainably efficient and equitable decision making, Nature Sustainability, https://doi.org/10.1038/s41893-020-0552-3

15. Fezzi, C., Bateman, I.J. (2011) Structural agricultural land use modelling for spatial agro-environmental policy analysis. *American Journal of Agricultural Economics*, 93(4): 1168-1188.

16. Gibson, M. (2012) Food Security—A Commentary: What Is It and Why Is It So Complicated? *Foods* 1(1): 18-27.

17. Peng, W. and Berry, E.M. (2019). The Concept of Food Security, in Ferranti, P., Berry, E. M. and Anderson, J. R. (Eds.) *Encyclopaedia of Food Security and Sustainability*. Oxford, Elsevier: 1-7.

18. FAO (2023). *Food Balances*. Rome, Food and Agriculture Organization of the United Nations.

19. Our World in Data (2024). Daily supply of calories per person. *Food Supply*. Oxford, University of Oxford.

20. National Health Service (2024). *Understanding calories*. UK, National Health Service.

21. Fezzi C., Bateman I.J., Hadley D., Harwood A. et al. (2019) Natural Environment Valuation Online Tool Technical Documentation, Chapter 1: Agriculture Model. Available at: https://www.leep.exeter.ac.uk/nevo/documentation/.

22. Defra (2018) Total income from farming – dataset. Available at: https://www.gov.uk/government/statistics/total-income-from-farming-in-the-uk (last access 09/2018).

23. Ritchie, P.D., Smith, G., Davis, K.J., Fezzi, C., Halleck-Vega, S., Harper, A., Boulton, C.A., Binner, A.R., Day, B.H., Gallego-Sala, A., Mecking, J.V., Sitch, S., Lenton, T.M. and Bateman, I.J. (2020) Shifts in national land use and food production in Great Britain after a climate tipping point, *Nature Food*, 1:76–83, doi:10.1038/s43016-019-0011-3.

24. Bateman, I.J., Anderson, K., Argles, A., Belcher, C., Betts, R.A., Binner, A., Brazier, R., Cho, F.H.T., Collins, R.M., Day, B.H., Duran-Rojas, C., Eisenbarth, S., Gannon, K., Gatis, N., Groom, B., Hails, R., Harper, A., Harwood, A., Hastings, A., Heard, M.S, Hill, T., Inman, A., Lee, C.F., Luscombe, D., Mackenzie, A.R., Mancini, M., Morison, J.I.L., Morris, A., Quine, C.P., Snowdon, P., Tyler, C.R., Vanguelova, E.I., Wilkinson, M., Williamson, D., Xenakis, G. (2022) A review of planting principles to identify the right place for the right tree for 'net zero plus' woodlands: Applying a place-based natural capital framework for Sustainable, Efficient and Equitable (SEE) decisions, *People and Nature*, 1–31, https://doi.org/10.1002/pan3.10331

25. Forestry Commission (2013a) *The Ecological Site Classification (ESC) Model*, Forestry Commission, Edinburgh, https://www.forestresearch.gov.uk/tools-and-resources/fthr/ecological-site-classification/

26. Pyatt, D. G, Ray, D., and Fletcher, J. (2001) An Ecological Site Classification for Forestry in Great Britain, *Forestry Commission Bulletin 124*, Forestry Commission, Edinburgh.

27. Ray, D, Suarez, J., and Pyatt, D. G. (1996). Development of an Ecological Site Classification System Decision Support System for British Forestry. Proceedings: 1st International Conference on GeoComputation. Leeds University.

28. Ray, D, Reynolds, K., Slade, J. and Hodge, S. (1998) A spatial solution to Ecological Site Classification for British Forestry using Ecosystem Management Decision Support, 3rd International Conference on GeoComputation, http://www.spatial.redlands.edu/emds/manuscripts/geocomp/geopap3.htm.

29. Monjur Murshed, S., Sliz, B., Montemurro, F., Vad, K., Bauen, A., Richter, G., Qi, A., Matthews, R., Eycott, A., Oliver, E., Taylor, G. and Tallis, M. Biomass Value Chain Modelling: Report on Tools and Data, ETI, (2011). https://doi.org/10.5286/UKERC.EDC.000032.

30. FC (2002) *National Inventory of Woodland Trees*. Forestry Commission, Edinburgh.

31. Edwards, P.N. and Christie, J.M. (1981) *Yield models for forest management*. Forestry Commission Booklet (FCBK048). Forestry Commission, Edinburgh.

32. Thompson, D. A, and Matthews, R. W. (1989). The storage of carbon in trees and timber. Forestry Commission Research Information Note 160, Forestry Commission, Edinburgh.

33. Forestry Commission (2013b) *Forest Investment Appraisal Package*, Forestry Commission, Edinburgh.

34. Lavers, G. M. and Moore, G. L. (1983) The strength properties of timber, *Building Research Establishment Report CI/Sfb i(J3)*, Building Research Establishment, Garston.

35. Bateman, I.J., Lovett, A.A. and Brainard, J.S. (2003) *Applied Environmental Economics: a GIS Approach to Cost-Benefit Analysis*, Cambridge University Press, Cambridge.

36. Gregersen, H. M, and Contreras, A. H. (1979). *Economic analysis of forest projects FAO Forestry Paper 17* (r. BiblioBazaar Ed.). Rome

37. Gregersen, H. M, & Contreras, A. H. (1992). *Economic assessment of forestry project impacts FAO Forestry Paper 106* Rome BiblioBazaar, reprinted 2011.

38. HM Treasury (2020) *The Green Book: Central Government Guidance on Appraisal and Evaluation*, HM Treasury, London

39. Black, J., Hashimzade, N. and Myles, G. (2009) A Dictionary of Economics (3rd ed.), Oxford University Press, Oxford.

40. Broadmeadow, S, and Nisbet, T. (2012). National map of woodland creation opportunities: targeting the WGS to help meet the objectives of the WFD and reduce flood risk in England. Forest Research, Alice Holt: Centre for Forestry and Climate Change.

41. Broadmeadow, M. (2002). Climate change: Impact on UK forests. Bulletin 125. Edinburgh http://www.forestry.gov.uk/fr/INFD-7KHELL: Forestry Commission.

42. Broadmeadow, M, and Ray, D. (2005). Climate change and British Woodland. http://www.forestry.gov.uk/fr/INFD-7KHELL: Forestry Commission Edinburgh.

43. Wood, S. N. (2003) Thin plate regression splines, *Journal of the Royal Statistical Society Series B,* 65, 95-114.

44. Fezzi, C. and Bateman, I.J. (2015) The Impact of Climate Change on Agriculture: Nonlinear Effects and Aggregation Bias in Ricardian Models of Farmland Values, *Journal of the Association of Environmental and Resource Economists*, 2(1): 57-92, doi/10.1086/680257

45. IPCC. (2006). *Guidelines for National Greenhouse Gas Inventories, Volume 4, Agriculture, Forestry and Other Land Use; Chapter 10*. http://www.ipcc-nggip.iges.or.jp/public/2006gl/vol4.html.

46. Li, C.S , Frolking, S., and Frolking, T. A. (1992). Measured and simulated nitrous oxide emissions from ryegrass- and ryegrass/white clover-based grasslands in a moist temperate climate. *Journal of Geophysical Research-Atmospheres, 97,* 9777-9783.

47. Coleman, K, and Jenkinson, D. S. (1996). RothC-26.3 - A model for the turnover of carbon in soil. In D. S. Powlson, P. Smith & J. U. Smith (Eds.), *Evaluation of soil organic matter models using existing, long-term datasets, NATO ASI Series I* (Vol. 38, pp. 237-246). http://www.rothamsted.ac.uk/ssgs/RothC/RothC.html: Springer.

48. Parton, W. J , Scurlock, W. J., Ojima, D. S., Gilmanov, T. G., Scholes, R. J., Schimel, D. S., Kirchner, T., Menaut, J-C., Seastedt, T., Garcia Moya, E. Kamnalrut, A. and Kinyamario, J.I. (1993) Observations and modelling of biomass and soil organic matter dynamics for the grassland biome worldwide, *Global Biogeochem. Cycles*, 7: 785-809.

49. CFA (2013) *Cool Farm Tool*, The Cool Farm Alliance, available at https://coolfarmtool.org/

50. Bouwman, A. F, Boumans, L. J. M., and Batjes, N. H. (2002). Modeling global annual N_2O and NO emissions from fertilized fields. *Global Biogeochemical Cycles*, *16*(4), 1080. doi: 10.1029/2001GB001812

51. FAO/IFA/IIASA/ISRIC-WSI/ISSCAS/JRC. (2013). *Harmonized World Soil Database (version 1.2)*. Retrieved from: http://webarchive.iiasa.ac.at/Research/LUC/External-World-soil-database/HTML/index.html?sb=1

52. WRI and WBCSD (2004) *The Greenhouse Gas Protocol, A Corporate Accounting and Reporting Standard*, *Revised Edition*, World Resources Institute and World Business Council for Sustainable Development, Washington DC and Geneva Switzerland.

53. Jenkinson, D.S. (1990) The turnover of organic carbon and nitrogen in soil, *Philosophical Transactions of the Royal Society B*, 329: 361-368

54. Hillier, J, Walter, C., Malin, D., Garcia-Suarez, T., Mila-i-Canals, L., & Smith, P. (2011). A farm-focused calculator for emissions from crop and livestock production. *Environmental Modelling and Software*, *26,* 1070-1078.

55. FAO/IFA. (2001). *Global Estimates of Gaseous Emissions of* NH_3*, NO and* N_2O *from Agricultural Land. First version*. Rome (http://faostat.fao.org/) FAO and IFA.

56. Audsley, E. (1997) *Harmonisation of environmental life cycle assessment for agriculture: Final Report,* Concerted Action AIR3-CT94-2028, European Commission, DG VI Agriculture, 139, http://www.ragusashwa.it/CD_2008/lavori/TOPIC2/orale/SPUGNOLI.pdf:

57. Whittaker, C, McManus, M. C., and Smith, P. (2013). A comparison of carbon accounting tools for arable crops in the United Kingdom. *Environmental Modelling and Software, 46*, 228-239.

58. DEFRA (2011a) *The British survey of fertiliser practice. Fertiliser use on farm crops for crop year 2011*, Department for Environment, Food & Rural Affairs, London.

59. IPCC. (2007b). *Climate Change 2007: The Physical Science Basis. Contribution of the Working Group I to the Forth Assessment Report of the Intergovernmental Panel on Climate Change,* IPCC, Geneva, Switzerland. http://www.ipcc.ch/publications_and_data/publications_ipcc_fourth_assessment_report_wg1_report_the_physical_science_basis.htm.

60. DEFRA (2011b) Greenhouse Gas Emission Projections for UK Agriculture to 2030. Economics Group, Department for Environment, Food & Rural Affairs, London.

61. Rogelj, J. et al., (2023) Credibility gap in net-zero climate targets leaves world at high risk. *Science* 380, 1014-1016.

62. IPCC (2019) *Climate Change and Land*, IPCC, Geneva.

63. Committee on Climate Change (2020) *Land use: Policies for a Net Zero UK*, Committee on Climate Change, London.

64. DeFries, R. et al., (2022) Land management can contribute to net zero. *Science* 376, 1163-1165.

65. Royal Society and Royal Academy of Engineering (2018) *Greenhouse Gas Removal*, Royal Society, London.

66. Woodland Carbon Code (2013) *Woodland Carbon Code. Requirements for voluntary carbon sequestration projects*, Forestry Commission, Edinburgh, http://www.forestry.gov.uk/pdf/WoodlandCarbonCode_Version_1.2.pdf/$FILE/WoodlandCarbonCode_Version_1.2.pdf

67. Matthews, R. W. and Broadmeadow, M. S. J. (2009) The potential of UK forestry to contribute to Government's emissions reduction commitments. In Read, D.J., Freer-Smith, P.H., Morison, J.I.L., Hanley, N., West, C.C. and Snowdon, P. (eds.) *Combating climate change – a role for UK forests. An assessment of the potential of the UK's trees and woodlands to mitigate and adapt to climate change*, The Stationery Office, Edinburgh, (pp. 139-161), https://www.tsoshop.co.uk/bookstore.asp?FO=1159966&Action=Book&ProductID=9780114973513

68. Bateman, I.J. and Lovett, A.A., (1998) Using geographical information systems (GIS) and large area databases to predict yield class: a study of Sitka spruce in Wales, *Forestry*, 71(2): 147-168.

69. Arora, V. K., Peng, Y., Kurz, W. A., Fyfe, J. C., Hawkins, B. and Werner, A. T. (2016). Potential near-future carbon uptake overcomes losses from a large insect outbreak in British Columbia. Canada, *Geophysical Research Letters*, 43(6), 2590–2598. https://doi.org/10.1002/2015GL067532.

70. Ciais, P., Sabine, C., Bala, G., Bopp, L., Brovkin, V., Canadell, J., Chhabra, A., DeFries, R., Galloway, J., Heimann, M., Jones, C., Le Quéré, C., Myneni, R.B., Piao, S. and Thornton, P. (2014). Carbon and other biogeochemical cycles. In: *Climate Change 2013: The Physical Science Basis*. Contribution of Working Group I to the Fifth Assessment Report of the

Intergovernmental Panel on Climate Change, Eds. Stocker, T.F., Qin, D., Plattner, G.-K., Tignor, M., Allen, S.K., Boschung, J., Nauels, A., Xia, Y., Bex V. and Midgley, P.M., Cambridge University Press, Cambridge, United Kingdom and New York, NY, USA.

71. Jones, A. G., Scullion, J., Ostle, N., Levy, P. E. and Gwynn-Jones, D. (2014). Completing the FACE of elevated CO2 research. *Environment International*, 73, 252–258. https://doi.org/10.1016/j.envint.2014.07.021.

72. Hararuk, O., Campbell, E.M., Antos, J.A. and Parish, R. (2019) Tree rings provide no evidence of a CO2 fertilization effect in old-growth subalpine forests of western Canada. *Glob Change Biol*. 25: 1222– 1234. https://doi.org/10.1111/gcb.14561

73. Boyd, I.L., Freer-Smith, P. H. Gilligan, C. A. and Godfray, H. C. J. (2013) The Consequence of Tree Pests and Diseases for Ecosystem Services, *Science*, 342: 1235773, DOI: 10.1126/science.1235773

74. Matthews, R. W. and Duckworth, R. R. (2005). BSORT: a Model of Tree and Stand Biomass Development and Production in Great Britain. In M. S. Imbabi & C. P. Mitchell (Eds.), *Proceedings of World Renewable Energy Congress (WREC 2005), 22-27 May 2005, Aberdeen, UK* (pp. 404-409), Elsevier. Oxford,

75. Matthews, R. W. (1991). Biomass production and carbon storage by British forests. In J. R. Aldhous (Ed.), *Wood for energy: the implications for harvesting, utilisation and marketing. Proceedings of a discussion meeting, Heriot-Watt University, Edinburgh, 5-7 April 1991* (pp. 162-177), Institute of Chartered Foresters, Edinburgh, UK.

76. Matthews, R. (2011) *Biomass Value Chain Model*, Forestry Commission, Alice Holt Lodge, Farnham.

77. Rollinson, T. J. D. and Gay, J. M. (1983) An Assortment Forecasting Service, *Forestry Commission Research Information Note 77/83/MENS*, Forestry Commission, Edinburgh, http://130.203.133.150/showciting;jsessionid=48986A5C6C40E9D7B8A5F45BBBDC7835?cid=10460328

78. Matthews, R. W. (1993). Towards a Methodology for the Evaluation of the Carbon Budget of Forests. Mimeo. Mensuration Branch, Forestry Commission, Alice Holt Lodge Research Station, Farnham, Surrey.

79. Matthews, R.W. and Mackie, E. D. (2006) *Forest Mensuration: a handbook for practitioners*. Forestry Commission, Edinburgh, UK.

80. McFadden D (1974) *Conditional logit analysis of qualitative choice behavior*, in Zarembka P. (ed.) *Frontiers in Econometrics*, Academic Press, New York, pp 105‑142.

81. Natural England (2019) *Monitor of Engagement with the Natural Environment: The national survey on people and the natural environment*, Technical Report, www.gov.uk/government/statistics/monitor-of-engagement-with-the-natural-environment-headline-report-andtechnical-reports-2018-to-2019

82. Forestry Commission (2021) *Forestry Statistics 2021: A compendium of statistics about woodland, forestry and primary wood processing in the United Kingdom*, Forest Research, Edinburgh, www.forestresearch.gov.uk/statistics/

83. OpenStreetMap Foundation (2022) *OpenStreetMap*, https://www.openstreetmap.org

84. Eaton, M.A., Brown, A.F., Noble, D.G., Musgrave, A.J., Hearn, R.D. Aebischer, N.J., Gibbons, D.W., Evans, A. and Gregory, R.D. (2009) Birds of Conservation Concern 3: the

population status of birds in the United Kingdom, Channel Islands and the Isle of Man, *British Birds*, 102: 296–341.

85. Risely, K., Massimino, D., Johnston, A., Newson, S. E., Eaton, M. A., Musgrove, A. J., Noble, D. G., Procter, D. and Baillie, S. R. (2012) The Breeding Bird Survey 2011, *BTO Research Report 624,* British Trust for Ornithology, Thetford.

86. Simpson, E.H. (1949) Measurement of diversity, *Nature*, 163: 688, doi:10.1038/163688a0

87. Gibbons, D.W., Reid, J.B. and Chapman, R.A. (1993) *The New Atlas of Breeding Birds in Britain and Ireland: 1988-1991*, Poyser, London.

88. SAS Institute Inc. (2013) The GENMOD Procedure, *SAS/STAT 13.1 User's Guide*, SAS Institute Inc., Cary, NC, USA

89. Burnham, K.P. and Anderson, D.R. (2002) *Model Selection and Multimodel Inference: A Practical Information-Theoretical Approach. 2d ed.*, Springer-Verlag, New York.

90. Burnham, K.P. and Anderson, D.R. (2004) Multimodel Inference: understanding AIC and BIC in model selection, *Sociological Methods and Research*, 33(2):261-304, doi:10.1177/0049124104268644

91. UKCP09 (2009a) *UK Climate Projections 2009*, data available from https://catalogue.ceda.ac.uk/uuid/094d9c9b9dda42c0aa1a1848af9fb56b

92. UKCP09 (2009b) *Climate Change Scenarios for the United Kingdom*, Tyndall Centre for Climate Change Research, School of Environmental Sciences, University of East Anglia, Norwich, UK.

93. Liedekerke, M. Van, Panagos, P. (2005) *ESDBv2 Raster Archive*, European Commission and the European Soil Bureau Network, Brussels.

94. Cormen, T.H., Leiserson, C.E., Rivest, R.L. and Clifford, S. Greedy Algorithms, *Introduction to Algorithms*, Fourth Edition, MIT Press, Cambridge, MA., pp.417-440, (2020).

95. Drezner, Z. and Hamacher, H.W. *Facility Location: Applications and Theory*, Springer-Verlag, New York (2002).

96. Bateman, I.J. et al. Bringing ecosystem services into economic decision making, *Science*, **341**, 45-50, (2013).

97. Andersen, P.D., Hansen, M. and Selin, C. (2021) Stakeholder inclusion in scenario planning—A review of European projects, *Technological Forecasting and Social Change*, 169: 120802, https://doi.org/10.1016/j.techfore.2021.120802.

98. Creedy, J., Doran, H., Duffield, S., George, N. and Kass, G. (2009) *England's natural environment in 2060 – issues, implications and scenarios*, Natural England, Sheffield.

99. Environment Agency (2009) *Water for people and the environment: Water resources strategy for England and Wales.* [online]. Available at: https://assets.publishing.service.gov.uk/government/uploads/system/uploads/attachment_data/file/289918/geho1209brlc-e-e.pdf

100. FEFAR (The Future of European Fisheries and Aquaculture Research) (2008) *Global review of horizon scanning and foresight exercises in the marine sector*. 1–42.

101. Foresight Land Use Futures (2010) *Making the most of land in the 21st century: Final project report*. The Government Office for Science, London.

102. Hulme, M., Jenkins, G.J., Lu, X., Turnpenny, J.R., Mitchell, T.D., Jones, R.G., Lowe, J., Murphy, J.M., Hassell, D., Boorman, P., McDonald, R. and Hill, S. (2002) *Climate Change Scenarios for the United Kingdom: The UKCIP02 Scientific Report*, Tyndall Centre for Climate Change Research, School of Environmental Sciences, University of East Anglia, Norwich, UK. 120pp.

103. MEA (Millennium Ecosystem Assessment) (2005) *Ecosystems and Human Wellbeing: Synthesis*, Island Press, Washington, DC.

104. Pinnegar, J., Viner, D., Hadley, D., Dye, S., Harris, M. and Simpson, F. (2006) *Alternative future scenarios for marine ecosystems: Technical report*. Centre for Environment, Fisheries and Aquaculture Science, Lowestoft.

105. Soste, L., Wang, Q.J., Robertson, D., Chaffe, R., Handley, S. and Wei, Y. (2015) Engendering stakeholder ownership in scenario planning, *Technological Forecasting and Social Change*, 91: 250-263. https://doi.org/10.1016/j.techfore.2014.03.002.

106. UK-NEA (2011) UK National Ecosystem Assessment: Technical Report. UNEP-WCMC, Cambridge, UK. Available via: http://uknea.unep-wcmc.org/

107. Moore, K., Haines-Young, R., Paterson, J., Potschin, M. and Silfwerbrand, G. (2010): Assessing User Needs Of the NEA Scenarios through Focal Questions, *CEM Working Paper No 4*, Centre for Environmental Management, University of Nottingham, UK.

108. Haines-Young, R. (2011) Exploring ecosystem service issues across diverse knowledge domains using Bayesian belief networks. *Progress in Physical Geography*. 35, 681–699, doi:10.1177/0309133311422977

109. Haines-Young, R., Paterson, J., Potschin, M., Wilson, A. and Kass, G. (2011) The UK NEA Scenarios: Development of Storylines and Analysis of Outcomes, in *The UK National Ecosystem Assessment: Technical Report*, UNEP-WCMC, Cambridge, pp. 1195-1264.
